# Mutualist- and antagonist-mediated selection contribute to trait diversification of flowers

**DOI:** 10.7717/peerj.14107

**Published:** 2022-09-29

**Authors:** Luyao Huang, Yang Liu, Liwen Dou, Shaobin Pan, Zhuangzhuang Li, Jin Zhang, Jia Li

**Affiliations:** 1 Shandong University of Traditional Chinese Medicine, Jinan, China; 2 Ocean University of China, Qingdao, China

**Keywords:** Antagonists, Mutualists, Evolution, Floral traits, Olfactory guides, Visual guides, Phenology

## Abstract

Flowers are generally short-lived, and they all face a multidimensional challenge because they have to attract mutualists, compel them to vector pollen with minimal investment in rewards, and repel floral enemies during this short time window. Their displays are under complex selection, either consistent or conflicting, to maximize reproductive fitness under heterogeneous environments. The phenological or morphological mismatches between flowers and visitors will influence interspecific competition, resource access, mating success and, ultimately, population and community dynamics. To better understand the effects of the plant visitors on floral traits, it is necessary to determine the functional significance of specific floral traits for the visitors; how plants respond to both mutualists and antagonists through adaptive changes; and to evaluate the net fitness effects of biological mutualisms and antagonism on plants. In this review, we bring together insights from fields as diverse as floral biology, insect behavioral responses, and evolutionary biology to explain the processes and patterns of floral diversity evolution. Then, we discuss the ecological significance of plant responses to mutualists and antagonists from a community perspective, and propose a set of research questions that can guide the research field to integrate studies of plant defense and reproduction.

## Introduction

Considering that flowers are the main component of the evolutionary innovation of flowering plants, exploring the phenotypic trait diversification of flowers has special significance for understanding the adaptability and diversity of populations (Schiestl & Johnson, 2013; Soltis & Soltis, 2014). Many studies have elucidated that the diversity of floral traits is largely a result of evolution alongside an even more diverse flower-associated community (Strauss & Whittall, 2006; Schiestl & Johnson, 2013). The community associated with flowers is extremely complex and varied, consists of mutualists such as pollinators, carnivores (including predators and parasitoids), and beneficial microorganisms (*e.g.*, nectar yeasts), as well as antagonists such as florivores, seed predators, nectar robbers and thieves, and pathogenic microorganisms (Aleklett, Hart & Shade, 2014; Rusman et al., 2019a; Boaventura et al., 2021). Visitors to flowers use a variety of floral traits to locate food sources, spawning, and predatory sites, and the behavior, growth, and innate and learned preferences of visitors lead to the continued evolution of floral traits (Rusman et al., 2019a).

Flowers have evolved diverse traits to attract animal mutualists and/or to deter antagonists to ensure reproductive success. These functions are maintained by a combination of flowering phenology, floral shape, floral display, floral color, floral scent and resources (Rusman et al., 2019a). It is generally believed that pollination mediated selection is the main factor affecting flower evolution (Benoit & Kalisz, 2020). The selection of non-pollinator agents can strengthen or oppose the selection of pollinators, so it will also affect the variation of floral traits (Roddy et al., 2020). The trade-off between reproduction and defense has fundamental impacts on the evolution of floral traits, as well as plant defense mechanisms and pollination systems (Schiestl et al., 2014). Different from the previous view that pollinators are the main evolutionary source of flowers, many studies have now adopted a pluralistic approach in which multiple sources of selection are considered in studies of floral evolution (Caruso et al., 2019; Moreira et al., 2019; Roddy et al., 2020; Kuppler & Kotowska, 2021). Therefore, to explain the processes and patterns of floral signal evolution, it is needed to structure in such a way that the effects of mutualists and antagonists were compared and contrasted to illustrate the potential fitness consequences of change in the community interactions.

The evolutionary diversity of floral traits is a means for plants to cope with environmental heterogeneity and seek survival, but evolution is diffuse when the selection on a given trait is dependent upon the broader community context in which a species exists. This means that shed light on the evolutionary forces of floral signals requires additional insights from the community dynamics. This review adopts a community perspective to understand the selection pressure and ecological implications of floral traits in multiple linked interaction units, which has practical significance for the continuation and protection of species. Beyond attempting a synthesis of current knowledge, we also point to areas in need of further research.

## Selection agents

### Mutualists

Mutualisms, defined as interspecific interactions that are beneficial to all the involved partners, have been a source of major evolutionary innovations (Pereira & Kjellberg, 2021). Mutualists of flowers are usually grouped according to the types of resources and services exchanged: transportation, protection, or nutrition, including pollinators, predators, parasitoids and beneficial microorganisms (Bronstein, Wilson & Morris, 2003; Rafferty, CaraDonna & Bronstein, 2015).

Pollination is the transfer of pollen from the anthers to the stigma of the carpel by the pollinator to fertilize the ovule and is typical of mutualism in plants (Bronstein, Alarcon & Geber, 2006). Pollinators evolved strategies to increase the exploitation of floral resources to their advantage, and play the role of pollen transporters in cross-pollinated plants (Bronstein, Alarcon & Geber, 2006; van der Kooi, Vallejo-Marin & Leonhardt, 2021). Certain plant-pollinator interactions are relatively specialized, but most are widely generalized (Rusman et al., 2018; Rusman et al., 2019b; Guimarães, 2020). In several highly specialized plant-insect interactions, scent-mediated specificity of pollinator attraction is directed by the emission and detection of volatile organic compounds (VOCs) (Proffit et al., 2020), such as the pollination mutualism between figs (*Ficus seymicordata*) and fig wasps (*Ceratosolen gravelyi*) (Chen et al., 2009). Schiestl and colleagues summarized three main types of pollinators’ responses to floral signals: receiver bias, adaptive innate preferences, and associative learning, which constitute the key selection environment for flower traits (Schiestl & Johnson, 2013; Schiestl, 2017). The preference of pollinators that have not evolved in the context of flower visitation is receiver bias. For example, receiver biases of some bees for radiating stripes, dark centers and peripheral dots may have evolved in the context of sexual selection rather than flower visitation (Van Kleunen et al., 2007). Both adaptive innate preferences and associative learning are related to pollinators’ pursuit of more rewards and appropriate oviposition site. Sensory preferences can be rapidly altered by associative learning, increasing foraging rewards (Schiestl & Johnson, 2013; Schiestl, 2017). But the relative benefit for plants *vs* pollinators ranges from solely beneficial for flower visitors (*e.g*., reward robbing), to more balanced interactions, to solely beneficial for plants (*e.g*., pollination by deception) (van der Kooi, Vallejo-Marin & Leonhardt, 2021).

The quantity or quality of rewards (*e.g.*, oil, pollen and nectar) and appropriate oviposition site offered by a flower have essential consequences for pollinator-mediated selection on floral signals. For instance, bumblebees learned and discriminated between different pollen types and casein using olfactory cues. When they touched the substances with their antennae, using chemotactile cues, they could also discriminate between different concentrations (Hagler, 1990; Ruedenauer, Spaethe & Leonhardt, 2015). But in some cases, due to the restriction of flower structure, pollinators cannot directly infer the reward status of a flower, nor can they directly infer the specific foraging cost associated with the flower (Von Arx et al., 2012; Haverkamp et al., 2016). For this reason, pollinators must rely on indirect floral signals, such as floral color and scents to predict the reward amounts of flowers (Hempel de Ibarra, Langridge & Vorobyev, 2015; Ollerton, 2017). *Ficus* produce urn-shaped inflorescences called figs that limit direct pollination behavior. Floral scent may provide an effective olfactory signal to fig (*Ficus semicordata*) pollinating wasps (*Ceratosolen gravelyi*) enabling them to infer the reward status (Chen et al., 2009). However, if the production of rewards causes a significant cost to plants and a decreased intra-individual correlation between floral signals and rewards allows them to reduce this cost, selection may favor plants with deceptive strategies (Benitez-Vieyra et al., 2010). Roddy et al. (2020) used an economics spectrum framework (‘economics’ refers to the cost-payback relationship that selection should favor) to identify the selective pressures shaping floral phenotypic variation, as the production and maintenance of flowers are energetically costly for plants (Roddy et al., 2020; Kuppler & Kotowska, 2021). But genetic constraint of plants, resource limitation and associative learning of pollinators may contribute to the maintenance of signal accuracy (Benitez-Vieyra et al., 2010). On the one hand, the existence of genetically based differences in signal accuracy among plants is a necessary condition for an evolutionary response mediated by pollinator selection (Ashman & Majetic, 2006). On the other hand, pollinators could be favoring the maintenance and reinforcement of the signal-reward correlation through a preference for those plants displaying more accurate signals, but the behavior increase foraging costs (Benitez-Vieyra et al., 2010).

Pollinator-mediated selection on floral signals is traditionally regarded as a common mechanism of adaptation and speciation in plants (Koski, 2020). Some findings indicated that the flower traits crucially depend on pollinator specialization and syndrome (Fenster et al., 2004; Ollerton, Winfree & Tarrant, 2011; Rosas-Guerrero et al., 2014; Dellinger, 2020). At first, animal pollinators will promote the evolution of floral traits, leading to the development of ‘pollination syndromes’, where the phylogenetically unrelated flowers match the preference of pollinators in shape, color, scent and size (Haverkamp et al., 2016). But the existence of pollinator syndromes to explain floral evolution and diversity is controversial, it just represents a specific hypothesis regarding the nature of floral variation and its ultimate causal roots (Ollerton et al., 2009). On the other hand, plants that are either too rare or insufficiently rewarding to their pollinators by imitating signals of other rewarding plants, including the signals of key food plants, animal mating partners, or oviposition sites, which would lead to advergent floral signal evolution (Gigord et al., 2002; Schiestl & Johnson, 2013). However, there are still some published studies proposed that the different pollinators all selected for a common floral trait, while some implied that a trait change that increases the fitness contribution of one pollinator will decrease the fitness of another (Sahli & Conner, 2011; Joly et al., 2018; Reich et al., 2020). Therefore, understanding the strength and shape of pollinator-mediated selection on flower traits is the first step to update the cognition of flower adaptive evolution (Schiestl & Johnson, 2013).

Carnivores (including predators and parasitoids) can benefit plant fitness by feeding on herbivores, which are indirect defense strategies of flowering plants against herbivores. Indirect defenses of flowers include the induced production of extra nectar that is exploited as a food source by carnivores and the induced emission of floral volatiles that attract carnivores (Dicke, 2000; Lucas-Barbosa, 2016). Tri-trophic interactions mediated by plant compounds are a classical approach to understanding plant indirect defense and have been described in many excellent reviews (Agrawal, 2000; Abdala-Roberts et al., 2019). Ant-plant protection mutualisms are model systems for examining the evolution and maintenance of plant defense strategies, species coexistence and multitrophic interactions (Trager et al., 2010). It has been shown that ants forage preferentially on plants with extrafloral nectaries. The visit of ants reduces the number of herbivorous insects on plants possessing extrafloral nectaries and thus lessens the damage by herbivores (Heil et al., 2001).

### Antagonists

Aside from pollinators, whose relationships with their host plants are by definition mutualisms, all other visitors may have antagonistic effects on plant reproduction (Galen, 1999). Antagonists range from large mammals to tiny insects, as well as microscopic bacteria and viruses, but most research in trait coevolution between antagonists and flowers generally focused on herbivores, florivores, and pathogens (Rusman et al., 2019a, 2019b). To survive, animal antagonists not only need to select high-quality food but also consider avoiding the damage caused by predators and parasitoids (Lucas-Barbosa et al., 2014). In this context, antagonists can alter the evolution of floral signals, either through direct fitness effects *via* the destruction of reproductive structures (*i.e.*, carpels and stamens) or indirectly through resource allocation trade-offs of plants between survival and reproduction (Schiestl & Johnson, 2013; Lucas-Barbosa, 2016).

As much as 18% of terrestrial plant biomass and 7% of flower biomass are consumed by herbivores, which makes herbivores an important driving force for the evolution of flower signals (McCall & Irwin, 2006; Boaventura et al., 2021). Foliar herbivory influences floral traits by changing plant resource allocation or by presenting secondary metabolites in flower signals and rewards (Kessler et al., 2013; Schiestl et al., 2014; Silveira et al., 2018). Herbivores might prefer to feed on flowers that could provide more resources to grow faster rather than on leaves; their larvae or adults can consume specific organs or the complete flower before seed coat formation (McCall & Irwin, 2006; Agerbirk et al., 2010; Silveira et al., 2018). Florivores can feed exclusively on flowers, but can also start feeding on leaves and then move to the flowers later in development, or switch diet when flowers become available (Lucas-Barbosa et al., 2014). Florivory generally causes varying degrees of damage to bracts, sepals, petals, stamens, pistils, as well as pollen and ovules (Inouye, 1980; Galen & Butchart, 2003; McCall & Irwin, 2006; Smallegange et al., 2007; Boaventura et al., 2021). The defense of plants is considered to be costly, not only because of the direct metabolic cost but also because of the indirect ecological cost (Cozzolino et al., 2015). For example, disruption of plant-pollinator interactions associated with inducible plant defense can be indirectly detrimental to plant fitness, conferring potential ecological costs, *e.g.*, ant-pollinator conflict (Kessler & Halitschke, 2009; Trager et al., 2010; Agrawal, 2011a; Lucas-Barbosa, 2016).

Antagonist-induced changes in floral signals also have an impact on several other plant-insect interactions, including a tendency to cause altered pollinator community composition and pollinator behavior, increased subsequent florivory and predators, indicating broad community-wide effects of floral damage for whole-plant interactions (Gorden & Adler, 2016; Rusman et al., 2018; Rusman et al., 2019b; Rusman et al., 2020). For example, flowers of cowpea emit volatiles attracting parasitoids (*Apanteles taragamae*) when attacked by herbivores (*Maruca vitrata*) (Dannon et al., 2010). Floral herbivores (caterpillars) in *Ruellia nudiflora* had significant detrimental effects on floral traits and plant attractiveness to pollinators (bees) (Moreira et al., 2019). But Cozzolino et al. (2015) found that the higher fruit set in foliar herbivore-infested *Silene latifolia* was caused by increased nocturnal pollinator attraction, mediated by the increased emission of pollinator-attracting floral volatiles. Thus, the effects of antagonists on plant reproductive fitness are not always negative, because plants can compensate herbivory with increased investment in pollinator attraction. In certain situations, mutualistic partners can also affect flowering plants indirectly by interfering with floral visitation, becoming antagonistic partners (Altshuler, 1999; Ness, 2006). For example, ant (*Ectatomma* ruidum and *Ectatomma* tuberculatum) attendance strongly improved fruit set of *Psychotria limonensis* by increasing the rate of pollinators’ visitation and preventing fruit loss to herbivorous insects. But carries costs to plants during fruit removal because bodyguard ants had a negative effect on the removal of ripe fruits by avian frugivores (Altshuler, 1999).

Plants have to induce direct and/or indirect defense responses to avoid a variety of damage from antagonists (Lucas-Barbosa et al., 2014). The concepts of resistance and tolerance have been used to understand how plants defend against antagonists directly (Strauss & Agrawal, 1999; McCall & Irwin, 2006; Irwin et al., 2010). Resistance is the ability to reduce the frequency of damage, including chemical deterrents, escape in space and time, physical barriers, and certain indirect resistance processes (Irwin, Adler & Brody, 2004; McCall & Irwin, 2006). The tolerance reflects the ability of plants to maintain fitness after damage, including compensatory flowers, resource reallocation, and improvement fecundity of individual flowers (Strauss & Agrawal, 1999; Ashman, 2002; Irwin et al., 2010). The indirect defenses that affect the antagonists through its natural enemies, carnivores, can be classified as mutualisms (Martinez-Bauer et al., 2015; Knauer, Bakhtiari & Schiestl, 2018; Rusman et al., 2019a). For example, ant-plants recruit ants by providing nesting sites and/or food resources and benefit from the ant-mediated reduction in damage by herbivores and pathogens (Trager et al., 2010). Although the fitness benefits through the attraction of carnivores are intuitive, the net effect of indirect defenses remains largely elusive on flowering plants, as it depends on the relative abundance of carnivores and herbivores in a plant population (Romero & Vasconcellos-Neto, 2004; Knauer, Bakhtiari & Schiestl, 2018; Villamil, Boege & Stone, 2019; Benoit & Kalisz, 2020). Also, the effect of carnivores on the host plant’s reproductive fitness by interfering with pollinators or directly damaging reproductive organs cannot be ignored (Martinez-Bauer et al., 2015; Knauer, Bakhtiari & Schiestl, 2018; Villamil, Boege & Stone, 2019; Benoit & Kalisz, 2020).

## Survey methodology

In this article, we reviewed the available academic articles published between 1980 and 2021 in the National Center for Biotechnology Information (NCBI), Web of Science, and the University’s databases (Ocean University of China) for books and journals.

We used “floral/flower mutualist” or “floral/flower antagonist” as a basic query and added terms “flowering phenology”, “floral/flower color”, “floral/flower display”, “floral/flower shape”, “floral/flower scent”, “floral/flower volatile organic compounds”, “pollinator”, “herbivore”, “florivore” or “predator” to search for information about the effect of mutualistic or antagonistic interactions on floral traits. We used “flower/ flowering”, “select/selection”, and “evolution” queries to search for information about the biotic factor that promotes the evolution of flowers. Reference lists of the included studies were hand-searched to identify any additional relevant studies.

The inclusion criteria were as follows: only English articles were included, and duplicates were removed *via* Endnote (X9). The title and abstract of the related studies were then screened in duplicate by two independent reviewers. After full-text screening for studies relevant to the evolution of floral diversity selected by insect mediators, we reduced the number of papers to 541. The publication dates of these studies ranged from 1980 to 2021, with a marked increase in the number from 2012–2021. Since there were excellent reviews describing the evolution of flowers in the early years, we mainly focus on the works from the past 10 years. Of 541 identified studies, 145 were included in the review. The title and abstract of the included studies were added to the Supplemental Material.

### Flowering phenology

For plants that live in seasonally changing environments, timing is critical (Blackman, 2017). The timing of flowering onset reflects the environmental conditions experienced during the development of pollen, ovules, and seeds, as well as the nature of interactions with mutualists and antagonists (Austen et al., 2017). Therefore, flowering phenology should be under the strong selective pressure of mutualists and antagonists, which combine together to signal the optimal time for reproduction (Körner & Basler, 2010; Block, Alexander & Levine, 2020).

Pollinators and predators are the main biotic selective agents acting on flowering phenology (Sercu et al., 2020). Decades of studies on natural plant populations have revealed a pervasive phenotypic selection of early flowering in the year, especially for plants that rely on pollinators (Munguia-Rosas et al., 2011; Austen et al., 2017; Sercu et al., 2020). Adamidis et al. (2019) elucidated that insect pollination increases the reproductive output of canola by advancing flower phenology, promoting a higher number of flowers at the peak of flowering. In general, the flowering time and the flowering duration are positively correlated, individuals that flower early often flower longer than those that flower late (Hendry & Day, 2005; Austen et al., 2017). However, while advancing phenology can help plants exploit longer reproductive seasons, allow earlier access to pollinators and resources, and avoid harsh environmental conditions later, can also result in mismatches with the timing of activity of mutualists and increase interspecific competition (Rafferty, CaraDonna & Bronstein, 2015; Austen et al., 2017; Block, Alexander & Levine, 2020). Moreover, delayed flowering is generally correlated with large, highly fecund flowers, whereas rapid flowering is correlated with small flowers that set fewer seeds (Kudoh et al., 2002; Austen et al., 2017). A study on *Mimulus guttatus* indicated that alternative alleles control trade-offs between “large size and slow flowering” and “small size and rapid flowering” (Troth et al., 2018). But Bemmels & Anderson (2019) showed that rapid flowering was genetically correlated with greater size at reproduction in *Boechera stricta*. However, the above two studies only reveal the selection of ‘time-size’ genetic variation by climate change, not ecological interactions. Considering the phenological synchrony within and between communities, the net impact of early flowering onset on individual reproduction and population dynamics is so far unclear.

Although natural selection for earlier flowering plants seems to be widespread, the significant directional selection was found to favor later flowering plants in wild sunflowers to avoid damage by a high abundance of herbivores (Pilson, 2000; Munguia-Rosas et al., 2011). In a review study, Elzing et al. (2007) found that in more than 80% of the tested species, pre-dispersal seed predators acting as selective agents in flower phenology favor off-peak or late flowering. This strategy of flowering plants to avoid antagonist-induced damage can be classified as phenology avoidance. There is growing evidence that antagonists visiting early in the season can also induce compensatory flowering *via* the production of a higher proportion of flowers within the same growing season (Brody & Irwin, 2012). In *Brassica rapa*, herbivore-infested plants produced more flowers during early flowering, effectively compensating for the lower olfactory attractiveness of flowers to pollinators (Schiestl et al., 2014). However, Sercu et al. (2020) indicated that there is no indication for within-season compensatory flowering with the effect of pre-dispersal seed predation in *Geum urbanum*, but the predation induces phenological avoidance in the subsequent year. In general, the induced compensatory flowering and phenological avoidance responses to herbivory are expected to be adaptive, and the two defense strategies are not necessarily mutually exclusive. In short, the optimal timing of flowering is a balance between avoiding the harsh environment and maximizing reproductive efficiency in the growing season. But the net consequences of altered biotic interactions will vary across species and ecosystems (Block, Alexander & Levine, 2020), and the results of studies on plant reproduction with early- and late-flowering species are inconsistent, with either positive, neutral, or negative effects, therefore, more research is needed in the future.

### Visual guides

Flowers have evolved multisensory (visual, olfactory) guides to mediate the attraction of pollinators and deterrence of antagonists, resulting in fitness advantages in terms of increased receipt and export of intraspecific pollen, and decreased damage caused by illegitimate visits (Schiestl, 2015; Harder et al., 2019). Visual guides, such as flower shape, display and color, and olfactory guides, such as flower volatiles, play a role in ensuring reproductive efficiency, which is fundamental to plant fitness.

#### Floral shape

The shape of flowers plays an irreplaceable role in the functional fit between the pollinator and the flower, which is related to the handling effectiveness in reward retrieval and pollen placement (Gómez et al., 2008a; Kaczorowski et al., 2012). At first, pollinators may directly promote the selection of flower shapes if the trait covaries with reward (pollen and nectar) (Gómez et al., 2008b; Gómez & Perfectti, 2010). Secondly, flower shape may depend on pollinator specialization (Reich et al., 2020; Niet, 2021). Specialized pollinators may promote the evolutionary transitions of floral shapes (Schemske & Bradshaw, 1999; Moyroud & Glover, 2017). For example, plants pollinated by hawkmoth *Agrius convolvuli* tend to have a very long and narrow flower tube or spur, white flowers and large volumes of dilute nectar (Johnson & Raguso, 2016). In lilies, large trumpet-shaped may have evolved as a response to selection by long-tongued hawkmoths, without excluding the short-tongued ones. This evolutionary pathway leads to a functionally more generalized pollination system instead of an increasingly specialized one (Liu et al., 2019). Furthermore, bilateral symmetry is thought to have evolved independently from radial symmetry as a consequence of strong selection exerted by specialized pollinators because it increases both flower attractiveness to pollinators and pollen transfer efficiency (Gómez, Perfectti & Camacho, 2006; Moyroud & Glover, 2017).

The change of flower shape is not only determined by pollinator-mediated selection. Although plants with specific morphology can be pollinated only by a set of pollinators, they can receive visits from floral visitors that remove pollen and/or nectar and did not pollinate the flower. They are commonly assumed to be detrimental to plant fitness because subsequent beneficial visitors seem likely to be deterred or to make shorter visits to drained flowers (Bronstein, Alarcon & Geber, 2006). Thus, these floral visitors can also exert selection on flower traits. In *Polemonium viscosum*, although bumblebees prefer open flared corollas, the final shape of the flower is a compromise to limit nectar thieving ants’ feeding (Galen & Butchart, 2003). Although floral shape has long been regarded as a factor in floral isolation and evolutionary shifts between pollinator affinities, more research on the fine structure of flowers is needed in the future to clarify the mode of the coevolution between interacting organisms and floral shape (Kaczorowski et al., 2012; Bronstein & Richman, 2015).

#### Floral display

Similar to flower shape, selection on flower display (including floral size, number of flowers and floral longevity) is rather a pluralistic process in which not only pollinators are involved, but also some antagonists (Galen, 1999; Teixido, Barrio & Valladares, 2016). In general, larger floral sizes are more attractive to many pollinators because the quantity and quality of nectar and pollen often positively correlate with corolla size, and larger sizes are more easily detected (Benitez-Vieyra et al., 2010; Venail, Dell’olivo & Kuhlemeier, 2010; Kaczorowski et al., 2012). Teixido, Barrio & Valladares (2016) have documented pollinator-mediated phenotypic selection for larger flowers. In the same way, the larger number of flowers has also been associated with higher pollinator attraction and, as a result, an increase in cross-pollination and reproductive success (Harder & Johnson, 2005; Teixido, Méndez & Valladares, 2011). In *Jasminum fruticans*, short-tongued bees showed a positive relationship between visitation rate and the number of open flowers, hawkmoths and butterflies made more visits to plants with larger flowers (Thompson, 2001). However, Sargent et al. (2007) proposed a negative trade-off between the size and number of flowers and inflorescences because of the resource costs. On the other hand, large floral displays have also to deal with additional costs imposed by antagonists that obtain food and rewards from plants without offering benefits to pollination (Teixido, Barrio & Valladares, 2016; Gélvez-Zúñiga et al., 2018). There is evidence that florivory increases with increasing components of plant attractiveness to pollinators such as the number of flowers displayed and flower size (Galen, 1999; Mosleh Arany, de Jong & van der Meijden, 2008). In the hummingbird-pollinated *Collaea cipoensis*, floral antagonists (ants and bees) exert negative selective pressures on flower size and number, counteracting pollinator-mediated selection on floral attractiveness traits (Gélvez-Zúñiga et al., 2018).

The longevity of a flower determines the probability and the number of times that a flower will be visited by pollinators, affects the total number of flowers open at any one time on the plant, with consequent implications for the level of outcrossing and the effectiveness of the overall floral display in attracting pollinators (Primack, 1985). Longer floral longevity should also increase the risk of florivory. In *Cistus ladanifer*, larger and longer-lived flowers tended to be affected by florivores more frequently, and moderate florivory levels open the possibility of exerting selection towards smaller and shorter-lived flowers (Teixido, Méndez & Valladares, 2011).

Why are the benefits of large, long-lived flowers so obvious, selections still favor tiny or shorter-lived flowers? First and foremost, the production and maintenance of large floral displays are highly costly, especially in areas with limited resources such as the Mediterranean (Teixido, Barrio & Valladares, 2016). Thus, documenting spatial variation of herbivory and pollination is important to understand differences in floral display related traits among populations (Teixido, Méndez & Valladares, 2011). Secondly, reducing the size of flowers is not always synonymous with reducing the floral signaling units. For example, the inflorescence of sunflower, which is composed of many greatly reduced flowers, can still produce dense clusters of flowers resembling large flowers to effectively attract pollinators (Moyroud & Glover, 2017). Thirdly, smaller or shorter-lived flowers may have potential advantages against antagonists (Galen, 1999; Teixido, Barrio & Valladares, 2016; Roguz et al., 2021). In *Trifolium repens*, herbivores weakened selection for increased inflorescence production, suggesting that large displays are costly in the presence of herbivores (Santangelo & Johnson, 2019).

#### Floral color

The traditional concept of pollinator syndrome also includes flower color, as a flower signal associated with particular kinds of pollinators (Campbell et al., 2010; Santangelo & Johnson, 2019). Different flower colors seem to be related to both the reliability of finding high nectar rewards and the average amount of sugar produced by particular flower species (Giurfa, Nunez & Backhaus, 1994; Raine et al., 2006; Raine & Chittka, 2007). Pollinator preference for different colors contributed to floral evolution and reproductive isolation (Schemske & Bradshaw, 1999; Sobral et al., 2015). In the New Zealand alpine, the insect pollinators show preferences based on color, leading to a high preponderance of white flowers in the area (Campbell et al., 2010). However, changes in floral color are not always driven by pollinators: in wild radish (*Raphanus sativus*), the pollinator preferences do not coincide with realized changes in flower color; florivores prefer white flowers to pink ones, which suggests that herbivores could also act as selective forces shaping floral color in nature (McCall et al., 2013).

When two agents are using the same color to select plants, the final display of flower color may be a compromise of plants to maximize reproduction. The opposed selection from pollinators and pre-dispersal seed predators maintains flower color variation in a population of *Gentiana lutea* (Veiga et al., 2015). In *Raphanus sativus*, differential preference and performance of herbivores (generalist and specialist *Lepidoptera*, slugs, aphids, and thrips) for color morphs may counteract selection on flower color exerted by pollinators (Irwin et al., 2003). An alternative hypothesis to pollinator- (or herbivore-) mediated selection is that flower color could be selected due to pleiotropic effects on other traits (Armbruster, 2002). Biochemical pathways that produce floral pigments (or volatiles) often also produce secondary compounds which are believed to protect plants from natural enemies or environmental stress (Brack, 1995; Fineblum & Rausher, 1997). For example, anthocyanins are omnipresent in angiosperms and probably evolved in early land plants long before the evolution of flowers. These pigments may have arisen in vegetative tissues in response to drought stress, heat stress and herbivore pressures, and were then subsequently co-opted by flowers to attract pollinators (Hanley, Lamont & Armbruster, 2009; Arista et al., 2013; Narbona et al., 2018). Thus, researchers could focus on more than one trait with the deepening of research, including color.

### Olfactory guides

Of all plant organs, flowers generally emit the highest amounts and most diverse blends of VOCs, which function as olfactory cues for the attraction of mutualists and/or the deterrence of antagonists to ensure plant reproductive success (Jurgens, Dotterl & Meve, 2006; Dudareva et al., 2013; Kessler et al., 2019). To date, over 1,700 VOCs have been identified from the headspace of flowers, which belong to seven major compound classes (Knudsen et al., 2006; Dudareva et al., 2013). The information transmitted by flower volatiles depends on the composition, content, and context of their emissions, and causes different behavioral responses of their visitors (Muhlemann, Klempien & Dudareva, 2014). Schiestl (2010) found an overlap of 87% in VOCs produced by plants and interacting insects. Analysis of the moth’s naturally attractive flowers shows that all volatiles are converged on a similar chemical profile, which in turn is uniquely reflected in the moth’s antennal lobe (Riffell et al., 2013). Similar to flower color we mentioned in the previous section, it is still unknown whether this similarity in secondary metabolites stems from pleiotropic effects on other traits.

There is evidence that the relatively simple change of flower fragrance can drive pollinator shifts and lead to rapid reproductive isolation of plants within a short period, such as in *Ficus carica* and *Ophrys arachnitiformis* x *O. lupercalis hybrids* (Vereecken, Cozzolino & Schiestl, 2010; Muhlemann, Klempien & Dudareva, 2014). VOCs analysis of pollination mutualism between the *Ficus carica* and its specific pollinator *Blastophaga psenes* revealed that a blend with a particular proportion of four of these VOCs is as attractive as the odor of receptive figs (Proffit et al., 2020). In *Ophrys*, variations in floral scent composition and proportions induced by the hybridization process can drive pollinator shifts and rapid reproductive isolation in highly specific plant-pollinator interactions (Vereecken, Cozzolino & Schiestl, 2010). Olfactory cues are particularly important for nocturnal visitors. Plants pollinated by nocturnal insects often exhibit characteristic odor compositions and temporal patterns (day/night) of emission (Balao et al., 2011). Temporal patterns of scent release can be species-specific and usually match the activity patterns of nocturnal pollinators (Dotterl, Wolfe & Jurgens, 2005). In nocturnally pollinated *Dianthus inoxianus*, the proportion of VOCs that elicited a physiological response differed between day and night. In moth-pollinated flowers (night-blooming), floral scents are often dominated by oxygenated terpenes and aromatic esters (Balao et al., 2011).

However, VOC production may also attract floral enemies beyond pollinators, and the emission of VOCs generates a trade-off to maximize reproduction (Schiestl & Johnson, 2013). If volatile attract both pollinators and antagonists, the fitness benefits of attracting pollinators by increasing volatiles emission may depend on the cost of attracting antagonists. In the wild *Texas gourd*, enhanced floral volatile can increase the attraction of detrimental florivores, rather than pollinators, and decrease plant reproduction (Theis & Adler, 2012). Also, certain volatiles may repel both floral enemies and pollinators. Nectar repellents (nicotine) in tobacco decreased nectaring time of pollinators and visiting frequency of nectar thieves, but increased pollinators’ visitation number, suggesting that there is a high variation of strategies to optimize reproduction (Kessler & Baldwin, 2007). In another research, both repellent (nicotine) and attractant (benzyl acetone) in tobacco were required to maximize pollinator visits and seed productivity (Kessler, Gase & Baldwin, 2008). In *Biscutella laevigata*, both pollinators (bees) and carnivores (crab spiders) were attracted by the floral volatile β-ocimene, the crab spider reduces bee visits to flowers but also benefits plants by feeding on florivores, demonstrating the context-dependence of selection (Knauer, Bakhtiari & Schiestl, 2018). Besides, because the biosynthesis of volatiles competes with the synthesis of defense compounds, antagonists may impose indirect selections on volatiles (Agrawal, 2011b). Antagonist-induced changes in the volatiles of flowers can affect the perception of plants by carnivores looking for prey and host. Flowers of cowpea emit volatiles attracting parasitoids (*Apanteles taragamae*) when attacked by herbivores (*Maruca vitrata*) (Dannon et al., 2010). Likewise, parasitoids of the pollen beetle use a volatile blend released by antagonist-attacked flowering rape to locate their herbivorous host (Jönsson & Anderson, 2007). In this way, flower scent can mediate predator-prey interactions, and both predator and prey are the driving forces on floral VOCs evolution.

Interestingly, pollinators themselves can also induce changes in the volatiles of flowers (Rodriguez-Saona et al., 2011). In *Brassica nigra*, pollination status influenced the profile of volatiles and changed the behavior of later butterflies (Lucas-Barbosa et al., 2015). In highbush blueberries, open-pollinated flowers were detected with less volatile emissions than pollinator-excluded flowers to reduce ecological costs. Otherwise, more volatile emissions may play a vital role in guiding pollinators to visit unpollinated flowers, which cannot only improve plant fitness but also increase the energy return of pollinators when they are foraging (Rodriguez-Saona et al., 2011). The reduction of volatile emission of flowers after pollination may be adaptive, which cannot only save the cost of scent production but also prevent further damage to flowers by later visitors (Muhlemann et al., 2006; Muhlemann, Klempien & Dudareva, 2014). Therefore, flowers can optimize visitors’ behavior through floral volatiles, and volatiles at least evolved under multiple biotic pressured exerted by mutualists and antagonists (Schiestl & Johnson, 2013).

## Conclusions

Ecological interaction and adaptation of flowering plants largely depend on flower traits. The diversity of traits likely results in a highly connected network of interactions within the complete flower-associated community, including flowers, mutualists, and antagonists. Changes in flower traits in response to each interacting visitor will alter multiple linked indirect interaction groups. The evolutionary diversity of flowers cannot be fully explained by a single medium alone, such as pollinators, but is driven by combinatorial selection imposed by associated communities. Likewise, focusing on herbivores alone cannot fully explain the evolution of plant defense traits, which may have originated from plant reproduction. To date, several published studies have adopted a community perspective to understanding the evolution of flowers, but these studies have limitations in demonstrating the adaptive consequences for plants. We still lack detailed knowledge about the relative degree to which these traits are affected by the insect visitors, how these traits contribute to the changes in interactors’ behavior, how these traits respond to sequential induction by different interactors and multiple interactors at the same time, and how much time adaptative/non-adaptative responses take to appear. Thus, except for common garden experiments, fitness consequences of flower responses to pollination, herbivory and parasitization need to be investigated in the field for a long time, where the full related community associated with the flower can be included.

Natural selection cannot possibly produce any modification in a species exclusively for the good of another species (Darwin, 1859). To fully understand the evolutionary process of floral diversity, tracing the evolutionary history of flowering plants and their visitors is as important as studying the adaptive consequences of floral signaling. However, the evolutionary mechanisms underlying the different sensory abilities of flowering plant visitors remain poorly understood. It is generally believed that the innate sensory preferences of the visitors are the result of their unilateral adaptation to flowers or mutual adaptation leading to co-evolution; however, some of these preferences did not evolve in the context of flower visits and are evolutionarily older than the signal itself. For example, most receiver biases of insects are related to animal communication in the context of sexual selection. In future research, it is necessary to take the evolutionary time or the evolutionary history of some groups or interactions into account, which help to explain adaptive evolution of flowering plants. Answers to these questions will facilitate the integration of evolutionary theories on plant survival and reproduction and help to explain floral trait diversity in response to multiple interactors.

## Supplemental Information

10.7717/peerj.14107/supp-1 Supplemental Information 1 The title and abstract of the included studies.

## Additional Information and Declarations

### Data Availability

The following information was supplied regarding data availability: This is a literature review and does not use raw data.
