# Supplementary material for: Mutualist- and antagonist-mediated selection contribute to trait diversification of flowers
Source: PeerJ. 2022 Sep 29;10:e14107. doi: 10.7717/peerj.14107 (PMC9527018; doi:10.7717/peerj.14107)
Supplement: Supplemental Information 1 [file peerj-10-14107-s001.docx]

Abdala-Roberts, L., et al. (2019). "Tri-trophic interactions: bridging species, communities and ecosystems." Ecol Lett **22**(12): 2151-2167.

A vast body of research demonstrates that many ecological and evolutionary processes can only be understood from a tri-trophic viewpoint, that is, one that moves beyond the pairwise interactions of neighbouring trophic levels to consider the emergent features of interactions among multiple trophic levels. Despite its unifying potential, tri-trophic research has been fragmented, following two distinct paths. One has focused on the population biology and evolutionary ecology of simple food chains of interacting species. The other has focused on bottom-up and top-down controls over the distribution of biomass across trophic levels and other ecosystem-level variables. Here, we propose pathways to bridge these two long-standing perspectives. We argue that an expanded theory of tri-trophic interactions (TTIs) can unify our understanding of biological processes across scales and levels of organisation, ranging from species evolution and pairwise interactions to community structure and ecosystem function. To do so requires addressing how community structure and ecosystem function arise as emergent properties of component TTIs, and, in turn, how species traits and TTIs are shaped by the ecosystem processes and the abiotic environment in which they are embedded. We conclude that novel insights will come from applying tri-trophic theory systematically across all levels of biological organisation.

Adamidis, G. C., et al. (2019). "Pollinators enhance crop yield and shorten the growing season by modulating plant functional characteristics: A comparison of 23 canola varieties." Scientific Reports **9**(1): 14208.

Insect pollination of flowers should change the within-season allocation of resources in plants. But the nature of this life-history response, particularly regarding allocation to roots, photosynthetic structures, and flowers, is empirically unresolved. This study uses a greenhouse experiment to investigate the effect of insect pollination on the reproductive output of 23 varieties of a globally important crop-canola (Brassica napus). Overall, insect pollination modified the functional characteristics (flower timing & effort, plant size & shape, seed packaging, root biomass) of canola, increasing seed production and quality, and pollinator dependence. Reproductive output and pollinator dependence were defined by strong trait trade-offs, which ranged from more pollinator-dependent plants favouring early reproductive effort, to less pollinator-dependent plants favouring a prolonged phenology with smaller plant size and lower seed quality. Seed production decreased with pollinator dependence in the absence of pollinators. The agricultural preference for hybrid varieties will increase seed production compared to open-pollinated varieties, but, even so, pollinators typically enhance seed production of both types. Our study elucidates how insect pollination alters the character and function of a globally important crop, supporting optimization of yield via intensification of insect pollination, and highlights the beneficial effects of insect pollination early in the season.

Agerbirk, N., et al. (2010). "Leaf and floral parts feeding by orange tip butterfly larvae depends on larval position but not on glucosinolate profile or nitrogen level." Journal of Chemical Ecology **36**(12): 1335-1345.

In an attempt to identify chemical signals governing the general flower and silique feeding behavior of larvae of the orange tip butterfly, Anthocharis cardamines (L.), we investigated feeding behavior and chemistry of two major host plants: Cardamine pratensis L. and Alliaria petiolata (Bieb.) Cavara & Grande (garlic mustard). Larvae reportedly feed mainly on flowers and siliques rather than leaves in nature, and did so when observed on the original host plants. Behavioral experiments, using detached A. petiolata branches, however, showed that larvae readily accepted leaves and only the final instar showed a tendency for directed movement towards floral parts. To search for semiochemicals that control plant part preference and to assess possible nutritional consequences of floral parts feeding, we determined glucosinolate profiles and total nitrogen levels of floral parts and leaves. There was only moderate difference between glucosinolate profiles of leaves and floral parts within each of two host plant species. In contrast, the profiles of floral parts differed significantly between them. A. petiolata was dominated by 2-propenyl glucosinolate, while C. pratensis was dominated by aromatic glucosinolates and branched aliphatic glucosinolates, with considerable variation among populations. Nitrogen levels tended to be higher in floral parts than in leaves in A. petiolata, but not in C. pratensis, so floral feeding could not generally be attributed to higher N content. With the exception of a tendency of last instar larvae (L5) to move to the apex and ingest flowers and upper stem, we did not find either a plant chemistry basis or larval acceptance/rejection behavior that could explain the usual feeding of floral parts by orange tip larvae of all instars. However, by artificial manipulation of vertical larval position on host plants, we found that the frequency of leaf vs. flower feeding during 24 hr depended significantly on the initial larval position. Hence, we suggest that the placement of eggs on floral parts by ovipositing female butterflies is a major explanation of orange tip feeding habits previously known from field observations.

Agrawal, A. A. (2000). "Mechanisms, ecological consequences and agricultural implications of tri-trophic interactions." Current Opinion in Plant Biology **3**: 329–335.

Recent research bridging mechanistic and ecological approaches demonstrates that plant attributes can affect herbivores, natural enemies of herbivores, and their interaction. Such effects may be genetically variable among plants and/or induced in individual plants by herbivore attack, and are mediated by primary plant attributes (i.e. nutritional quality and physical structure) and defense-related products (i.e. secondary chemicals and plant volatiles), and may be modified by human activity (e.g. by the introduction of Bacillus thuringiensis). The study of tri-trophic interactions is important in order to understand natural species interactions and to manipulate these interactions in pest control.

Agrawal, A. A. (2011). "Current trends in the evolutionary ecology of plant defence." Functional Ecology **25**: 420–432.

1. In this essay I summarize current trends in the evolutionary ecology of plant defence, while advocating for approaches that integrate community ecology with specific tests of classic evolutionary hypotheses. Several conclusions emerge. 2. The microevolution of defence is perhaps best studied by reciprocal transplant experiments of differentiated plant populations while simultaneously manipulating the presence of the herbivore(s) hypothesized to be the agent(s) of natural selection. 3. Although there is continued interest in the costs of defence, I argue that some empirical approaches to estimating costs (e.g. genetic engineering) may provide limited insight into evolutionary processes. 4. Essentially all plants employ several different lines of defence against herbivory. It is thus time to abandon searching for single silver bullet traits and the simple trade-off model (where traits are arbitrarily expected to negatively covary across genotypes or species). We still know very little about which trait combinations are most effective and have repeatedly evolved together. Thus, some of our prominent theories (e.g. a predicted trade-off between direct and indirect defence) need to be revised. 5. Studies of the macroevolution of plant defence are enjoying renewed interest due to available phylogenies and analytical methods. Although general trends are not currently surmisable, we will soon have strong case studies evaluating both biotic and abiotic drivers ofconvergent evolution in defence strategies and the role ofdefence evolution in the adaptive radiation ofplant lineages. 6. The evolution of specificity is proposed as a final frontier in understanding complexity in plant–herbivore interactions. Although it is abundantly clear that plants can deploy highly specific defensive responses that are differentially perceived by herbivore species, how such responses evolve and are physiologically regulated remains an important gap. Relatively straightforward methodologies are now available to close the loop between plant perception of herbivory, hormonal responses, and production ofdefensive end-products across genotype or species.

Agrawal, A. A. (2011). "New Synthesis—Trade-offs in Chemical Ecology." Journal of Chemical Ecology **37**(3): 230-231.

Aleklett, K., et al. (2014). "The microbial ecology of flowers: An emerging frontier in phyllosphere research." Botany.

Though we are learning more about the diversity, dynamics, and importance of phyllosphere microbiota, we have only modest knowledge about the microorganisms that specifically inhabit flowers. Due to their ephemerality and exquisite anatomy, flowers provide unique habitats to microorganisms, including a range of distinct microscale niches. Here, we review the recent literature concerning community composition and diversity in the flower microbiota, the spatial and temporal community dynamics, and the interactions between flower microbes, their plant hosts, and pollinators. We conclude with future directions for improving our understanding of this emerging frontier in phyllosphere microbial ecology.

Altshuler, D. (1999). "Novel interactions of non-pollinating ants with pollinators and fruit consumers in a tropical forest." Oecologia **119**(4): 600-606.

The tropical ants Ectatomma ruidum and E. tuberculatum (Formicidae) regularly patrol leaves, ¯owers, and fruits of the understory shrub, Psychotria limonensis (Rubiaceae), on Barro Colorado Island, Panama. Ant and pollinator exclusion experiments elucidated both positive and negative eects of ant attendance on plant reproductive success, including pollination, fruit set, fruit loss, and fruit removal. Ants did not pollinate ¯owers but did contribute to higher pollination success, probably by increasing the relocation frequency of winged pollinators and thus the rate of ¯ower visitation. Ants also prevented fruit loss to herbivorous insects which were common during the early stages of fruit development. Thus, ant attendance strongly improved both pollination and fruit set whereby plants with ants set more fruit per ¯ower and also lost fewer fruits during fruit maturation. In contrast, ants had a negative eect on the removal of ripe fruits by avian frugivores. Thus, ant attendance has a non-trivial in¯uence on plant reproduction, this interaction being bene®cial at some stages of the plant reproductive cycle and carrying costs at another stage. A tight ecological or co-evolved relationship between these Ectatomma spp. and P. limonensis is unlikely given that ant attendance of plants is detrimental to fruit removal.

Andres, F. and G. Coupland (2012). "The genetic basis of flowering responses to seasonal cues." Nature Reviews Genetics **13**(9): 627-639.

Plants respond to the changing seasons to initiate developmental programmes precisely at particular times of year. Flowering is the best characterized of these seasonal responses, and in temperate climates it often occurs in spring. Genetic approaches in Arabidopsis thaliana have shown how the underlying responses to changes in day length (photoperiod) or winter temperature (vernalization) are conferred and how these converge to create a robust seasonal response. Recent advances in plant genome analysis have demonstrated the diversity in these regulatory systems in many plant species, including several crops and perennials, such as poplar trees. Here, we report progress in defining the diverse genetic mechanisms that enable plants to recognize winter, spring and autumn to initiate flower development.

Arista, M., et al. (2013). "Abiotic factors may explain the geographical distribution of flower colour morphs and the maintenance of colour polymorphism in the scarlet pimpernel." Journal of Ecology **101**(6): 1613-1622.

Flower colour polymorphism is traditionally attributed to pollinator selection although other factors, such as indirect selection on correlated traits, can play an important role.Lysimachia arvensis is a widespread annual species with two colour morphs differing in anthocyanin composition. We explored the hypothesis that colour polymorphism is maintained by selection related to environmental heterogeneity. Morph frequencies and environmental traits were recorded in 51 populations along a wide geographical range. To explore the existence of morph-by-environment interactions, we conducted an experimental study comparing the two morphs under treatments differing in water and light availability.A geographical pattern was found with a negative association between blue frequencies and latitude. The proportion of the blue morph increased with temperature and sunshine hours, but decreased with precipitation. Flowering onset and flower size differed between morphs and scarcely varied across treatments. In contrast, several fitness components such as germination, seedling survival, seedling mass and flower production showed important morph-by-environment interactions. The blue morph showed higher overall male and female fitness in all the treatment combinations excepting in sun-wet conditions where the red morph had higher fitness.Synthesis. Our results indicate that the mechanism of selection on flower colour seems to be related to differences in fitness of both morphs due to abiotic factors. These differences could explain the geographical distribution of flower colour morphs and the maintenance of the colour polymorphism. The marked difference in flowering time between morphs leaves open the potential for assortative mating and speciation in Lysimachia arvensis.

Armbruster, W. S. (2002). "Can indirect selection and genetic context contribute to trait diversification? A transition-probability study of blossom-colour evolution in two genera." Journal of Evolutionary Biology **15**(3): 468-486.

Darwin recognized that biological diversity has accumulated as a result of both adaptive and nonadaptive processes. Very few studies, however, have addressed explicitly the contribution of nonadaptive processes to evolutionary diversification, and no general procedures have been established for distinguishing between adaptive and nonadaptive processes as sources of trait diversity. I use the diversification of flower colour as a model system for attempting to identify adaptive and nonadaptive causes of trait diversification. It is widely accepted that variation in flower colour reflects direct, adaptive response to divergent selective pressures generated by different pollinators. However, diversification of flower colour may also result from the effects of nonadaptive, pleiotropic relationships with vegetative traits. Floral pigments that have pleiotropic relationships to vegetative pigments may evolve and diversify in at least two nonadaptive ways. (1) Indirect response to selection on the pleiotropically related nonfloral traits may occur (indirect selection). (2) Divergent evolution in response to parallel selective pressures (e.g. selection by pollinators for visually obvious flowers) may occur because populations are at different genetic starting points, and each population follows its own genetic ‘line of least resistance.’ A survey of literature suggests that pleiotropic relationships between flower colour and vegetative traits are common. Phylogenetically informed analyses of comparative data from Dalechampia (Euphorbiaceae) and Acer (Aceraceae), based on trait-transition probabilities and maximum likelihood, indicated that floral and vegetative pigments are probably pleiotropically related in these genera, and this relationship better explains the diversification of floral colour than does direct selection by pollinators. In Dalechampia pink/purple floral bract colour may have originated by indirect response to selection on stem and leaf pigments. In Acer selection by pollinators for visually obvious flowers may to have led to the evolution of red or purple flowers in lineages synthesizing and deploying red anthocyanins in leaves, and pale-green or yellow flowers in species not deploying red anthocyanins in vegetative structures. This study illustrates the broader potential of indirect selection and parallel selection on different genetic starting points to contribute to biological diversity, and the value of testing directly for the operation of these nonadaptive diversifying processes.

Ashman, T.-l. (2002). "The role of herbivores in the evolution of separate sexes from hermaphroditism." Ecology **85**(5): 1175-1184.

Evolutionary biologists have repeatedly argued that environmental factorshave influenced the evolution of dioecy (males and females) from hermaphroditism in plants. While many researchers have focused on the role of pollinators and abiotic factors, far fewer have considered the enemy component of the environment. In this paper, I explore how herbivory may impact the evolution of dioecy from hermaphroditism through gynodioecy. This synthesis shows that the widespread occurrence of male-biased enemy attack in sexually dimorphic species is likely to have significant consequences for both the first and second steps in the evolution of dioecy through gynodioecy. I identify several ways that herbivory can influence sexual-system evolution, review the handful of studies that have explored them, and highlight exciting new avenues for research. From this review and synthesis, I conclude that consideration of enemies will provide a fresh dimension to our understanding of plant sexual systems.

Ashman, T. L. and C. J. Majetic (2006). "Genetic constraints on floral evolution: a review and evaluation of patterns." Heredity (Edinb) **96**(5): 343-352.

The characteristics of flowers influence most aspects of angiosperm reproduction, including the agents of pollination and patterns of mating. Thus, a clear view of the forces that mediate floral phenotypic evolution is central to understanding angiosperm diversity. Here, we inform on the capacity for floral phenotype to respond to selection by reviewing published data on heritabilities and genetic correlations for several classes of floral traits (primary sexual, attraction, mating system) in hermaphroditic plants. We find significant heritability for all floral traits but also variation among them, as well as a tendency for heritability to vary with mating system, but not life history. We additionally test predictions stemming from life history theory (eg, negative covariation between male-female traits and flower size-flower number), and ideas concerning the extent and pattern of genetic integration between flowers and leaves, and between the sexes of dioecious and gynodioecious species. We find mixed evidence for life history tradeoffs. We find strong support for floral integration and its relation with floral morphology (actinomorphy vs zygomorphy) and for a decoupling of floral and vegetative traits, but no evidence that modular integration varies with floral morphology. Lastly, we find mixed evidence for a relationship between the level of sexual dimorphism in attraction traits and the between-sex correlation in gender dimorphic plants.

Austen, E. J., et al. (2017). "Explaining the apparent paradox of persistent selection for early flowering." New Phytologist **215**(3): 929-934.

Decades of observation in natural plant populations have revealed pervasive phenotypic selection for early flowering onset. This consistent pattern seems at odds with life-history theory, which predicts stabilizing selection on age and size at reproduction. Why is selection for later flowering rare? Moreover, extensive evidence demonstrates that flowering time can and does evolve. What maintains ongoing directional selection for early flowering? Several non-mutually exclusive processes can help to reconcile the apparent paradox of selection for early flowering. We outline four: selection through other fitness components may counter observed fecundity selection for early flowering; asymmetry in the flowering-time– fitness function may make selection for later flowering hard to detect; floweringtimeandfitnessmaybecondition-dependent;and selection on flowering duration is largely unaccounted for. In this Viewpoint, we develop these four mechanisms, and highlight areas where further study will improve our understanding of floweringtime evolution.

Balao, F., et al. (2011). "Spatial and temporal patterns of floral scent emission in Dianthus inoxianus and electroantennographic responses of its hawkmoth pollinator." Phytochemistry **72**(7): 601-609.

Scent emission is important in nocturnal pollination systems, and plant species pollinated by nocturnal insects often present characteristic odor compositions and temporal patterns of emission. We investigated the temporal (day/night; flower lifetime) and spatial (different flower parts, nectar) pattern of flower scent emission in nocturnally pollinated Dianthusinoxianus, and determined which compounds elicit physiological responses on the antennae of the sphingid pollinator Hyles livornica. The scent of D.inoxianus comprises 68 volatile compounds, but is dominated by aliphatic 2-ketones and sesquiterpenoids, which altogether make up 82% of collected volatiles. Several major and minor compounds elicit electrophysiological responses in the antennae of H. livornica. Total odor emission does not vary along day and night hours, and neither does along the life of the flower. However, the proportion of compounds eliciting physiological responses varies between day and night. All flower parts as well as nectar release volatiles. The scent of isolated flower parts is dominated by fatty acid derivatives, whereas nectar is dominated by benzenoids. Dissection (= damage) of flowers induced a ca. 20-fold increase in the rate of emission of EAD-active volatiles, especially aliphatic 2-ketones. We suggest that aliphatic 2-ketones might contribute to pollinator attraction in D. inoxianus, even though they have been attributed an insect repellent function in other plant species. We also hypothesize that the benzenoids in nectar may act as an honest signal ('nectar guide') for pollinators.

Bartomeus, I., et al. (2011). "Climate-associated phenological advances in bee pollinators and bee-pollinated plants." Proceedings of the National Academy of Sciences, USA **108**(51): 20645-20649.

The phenology of many ecological processes is modulated by temperature, making them potentially sensitive to climate change. Mutualistic interactions may be especially vulnerable because of the potential for phenological mismatching if the species involved do not respond similarly to changes in temperature. Here we present an analysis of climate-associated shifts in the phenology of wild bees, the most important pollinators worldwide, and compare these shifts to published studies of bee-pollinated plants over the same time period. We report that over the past 130 y, the phenology of 10 bee species from northeastern North America has advanced by a mean of 10.4 +/- 1.3 d. Most of this advance has taken place since 1970, paralleling global temperature increases. When the best available data are used to estimate analogous rates of advance for plants, these rates are not distinguishable from those of bees, suggesting that bee emergence is keeping pace with shifts in host-plant flowering, at least among the generalist species that we investigated.

Bemmels, J. B. and J. T. Anderson (2019). "Climate change shifts natural selection and the adaptive potential of the perennial forb Boechera stricta in the Rocky Mountains." Evolution **73**(11): 2247-2262.

Heritable genetic variation is necessary for populations to evolve in response to anthropogenic climate change. However, antagonistic genetic correlations among traits may constrain the rate of adaptation, even if substantial genetic variation exists. We examine potential genetic responses to selection by comparing multivariate genetic variance-covariances of traits and fitness (multivariate Robertson-Price identities) across different environments in a reciprocal transplant experiment of the forb Boechera stricta in the Rocky Mountains. By transplanting populations into four common gardens arrayed along an elevational gradient, and exposing populations to control and snow removal treatments, we simulated future and current climates and snowmelt regimes. Genetic variation in flowering and germination phenology declined in plants moved downslope to warmer, drier sites, suggesting that these traits may have a limited ability to evolve under future climates. Simulated climate change via snow removal altered the strength of selection on flowering traits, but we found little evidence that genetic correlations among traits are likely to affect the rate of adaptation to climate change. Overall, our results suggest that climate change may alter the evolutionary potential of B. stricta, but reduced expression of genetic variation may be a larger impediment to adaptation than constraints imposed by antagonistic genetic correlations.

Benitez-Vieyra, S., et al. (2010). "Selection on signal-reward correlation: limits and opportunities to the evolution of deceit in Turnera ulmifolia L." Journal of Evolutionary Biology **23**(12): 2760-2767.

Because pollinators are unable to directly assess the amount of rewards offered by flowers, they rely on the information provided by advertising floral traits. Thus, having a lower intra-individual correlation between signal and reward (signal accuracy) than other plants in the population provides the opportunity to reduce investment in rewards and cheat pollinators. However, pollinators' cognitive capacities can impose a limit to the evolution of this plant cheating strategy if they can punish those plants with low signal accuracy. In this study, we examined the opportunity for cheating in the perennial weed Turnera ulmifolia L. evaluating the selective value of signal accuracy, floral display and reward production in a natural population. We found that plant reproductive success was positively related to signal accuracy and floral display, but not to nectar production. The intensity of selection on floral display was more than three times higher than on signal accuracy. The pattern of selection indicated that pollinators can select for signal accuracy provided by plants and suggests that learning abilities of pollinators can limit the evolution of deceptive strategies in T. ulmifolia.

Benoit, A. D. and S. Kalisz (2020). "Predator Effects on Plant-Pollinator Interactions, Plant Reproduction, Mating Systems, and Evolution." Annual Review of Ecology, Evolution, and Systematics **51**(1): 319-340.

Plants are the foundation of the food web and therefore interact directly and indirectly with myriad organisms at higher trophic levels. They directly provide nourishment to mutualistic and antagonistic primary consumers (e.g., pollinators and herbivores), which in turn are consumed by predators. These interactions produce cascading indirect effects on plants (either trait-mediated or density-mediated).We review how predators affect plant-pollinator interactions and thus how predators indirectly affect plant reproduction, fitness, mating systems, and trait evolution. Predators can influence pollinator abundance and foraging behavior. In many cases, predators cause pollinators to visit plants less frequently and for shorter durations. This decline in visitation can lead to pollen limitation and decreased seed set. However, alternative outcomes can result due to differences in predator, pollinator, and plant functional traits as well as due to altered interaction networks with plant enemies. Furthermore, predators may indirectly affect the evolution of plant traits and mating systems.

Blackman, B. K. (2017). "Changing Responses to Changing Seasons: Natural Variation in the Plasticity of Flowering Time." Plant Physiology **173**(1): 16-26.

Block, S., et al. (2020). "Phenological plasticity is a poor predictor of subalpine plant population performance following experimental climate change." OIKOS **129**(2): 184-193.

Phenological shifts, changes in the seasonal timing of life cycle events, are among the best documented responses of species to climate change. However, the consequences of these phenological shifts for population dynamics remain unclear. Population growth could be enhanced if species that advance their phenology benefit from longer growing seasons and gain a pre-emptive advantage in resource competition. However, it might also be reduced if phenological advances increase exposure to stresses, such as herbivores and, in colder climates, harsh abiotic conditions early in the growing season. We exposed subalpine grasslands to ~ 3 K of warming by transplanting intact turfs from 2000 m to 1400 m elevation in the eastern Swiss Alps, with turfs transplanted within the 2000 m site acting as a control. In the first growing season after transplantation, we recorded species' flowering phenology at both elevations. We also measured species' cover change for three consecutive years as a measure of plant performance. We used models to estimate species' phenological plasticity (the response of flowering time to the change in climate) and analysed its relationship with cover changes following climate change. The phenological plasticity of the 18 species in our study varied widely but was unrelated to their changes in cover. Moreover, early- and late-flowering species did not differ in their cover response to warming, nor in the relationship between cover changes and phenological plasticity. These results were replicated in a similar transplant experiment within the same subalpine community, established one year earlier and using larger turfs. We discuss the various ecological processes that can be affected by phenological shifts, and argue why the population-level consequences of these shifts are likely to be species- and context-specific. Our results highlight the importance of testing assumptions about how warming-induced changes in phenotypic traits, like phenology, impact population dynamics.

Boaventura, M. G., et al. (2021). "Revisiting florivory: an integrative review and global patterns of a neglected interaction." New Phytol.

Florivory is an ancient interaction which has rarely been quantified due to a lack of standardized protocols, thus impairing biogeographical and phylogenetic comparisons. We created a global, continuously updated, open-access database comprising 180 species and 64 families to compare floral damage between tropical and temperate plants, to examine the effects of plant traits on floral damage, and to explore the eco-evolutionary dynamics of flower-florivore interactions. Flower damage is widespread across angiosperms, but was two-fold higher in tropical vs temperate species, suggesting stronger fitness impacts in the tropics. Flowers were mostly damaged by chewers, but neither flower color nor symmetry explained differences in florivory. Herbivory and florivory levels were positively correlated within species, even though the richness of the florivore community does not affect florivory levels. We show that florivory impacts plant fitness via multiple pathways and that ignoring this interaction makes it more difficult to obtain a broad understanding of the ecology and evolution of angiosperms. Finally, we propose a standardized protocol for florivory measurements, and identify key research avenues that will help fill persistent knowledge gaps. Florivory is expected to be a central research topic in an epoch characterized by widespread decreases in insect populations that comprise both pollinators and florivores.

Brack, D. L. E. (1995). "Natural selection against white petals in Phlox." Evolution **49**(5): 1017-1022.

Brody, A. K. and R. E. Irwin (2012). "When resources don't rescue: flowering phenology and species interactions affect compensation to herbivory in Ipomopsis aggregata." OIKOS **121**(9): 1424-1434.

The ability of plants to tolerate, or compensate for, herbivore damage is highly variable and has been the subject of much research. Although many plants can compensate for herbivore damage, and some even overcompensate, we cannot yet generalize about the conditions that promote a positive response to damage. Here, we asked how abiotic resources (i.e. plant nutrient status) coupled with biotic interactions – i.e. subsequent interactions with pollinators, seed predators and nectar robbing bumble bees – aff ect the compensatory ability of Ipomopsis aggregata , a monocarpic herb that has been the subject of much previous debate. We hypothesized that compensation to herbivore damage in I. aggregata (Polemoniaceae) would depend fi rst on plants having an ample supply of resources and, second, on the outcome of subsequent interactions with mutualist pollinators and enemy pre-dispersal seed predators and nectar robbing bumble bees. We used a fully-factorial experiment in which plants were watered, fertilized or left as unmanipulated controls, crossed with clipping to simulate herbivore damage to the apical meristem. Resource addition enhanced both male and female components of fi tness, but resource enhancement did not provide the means for plants to fully compensate for simulated herbivory. Clipped plants produced signifi cantly more infl orescences, but at the expense of a delay in fl owering and fewer total fl owers. Clipping signifi cantly reduced losses to dipteran pre-dispersal seed predators by delaying fl owering time, but early fl owering plants produced higher numbers of seeds despite incurring higher rates of predation. Clipped plants incurred a higher risk to nectar robbers in one of two years. Overall, clipped plants suff ered severe reductions (a nearly 50% reduction in total seed set) in female success, but clipping combined with nutrient addition enhanced male function through increases in per-fl ower pollen production. However, because clipped plants produced signifi cantly fewer fl owers than unclipped plants, whole-plant pollen production was signifi cantly reduced by clipping. Pollinator visitation and nectar robbing were variable between clipping treatments and between years and (nectar robbing) among sites. Our results demonstrate that the variability in plant response to herbivory can, at least in part, be driven by plant interactions with mutualists and enemies. Th us, accounting for such interactions and their variability is important to fully understanding plant compensation for herbivore damage and will likely go far to explain variation in plant response that appears to be independent of resources.

Bronstein, J. L., et al. (2006). "The evolution of plant-insect mutualisms." New Phytol **172**(3): 412-428.

Mutualisms (cooperative interactions between species) have had a central role in the generation and maintenance of life on earth. Insects and plants are involved in diverse forms of mutualism. Here we review evolutionary features of three prominent insect-plant mutualisms: pollination, protection and seed dispersal. We focus on addressing five central phenomena: evolutionary origins and maintenance of mutualism; the evolution of mutualistic traits; the evolution of specialization and generalization; coevolutionary processes; and the existence of cheating. Several features uniting very diverse insect-plant mutualisms are identified and their evolutionary implications are discussed: the involvement of one mobile and one sedentary partner; natural selection on plant rewards; the existence of a continuum from specialization to generalization; and the ubiquity of cheating, particularly on the part of insects. Plant-insect mutualisms have apparently both arisen and been lost repeatedly. Many adaptive hypotheses have been proposed to explain these transitions, and it is unlikely that any one of them dominates across interactions differing so widely in natural history. Evolutionary theory has a potentially important, but as yet largely unfilled, role to play in explaining the origins, maintenance, breakdown and evolution of insect-plant mutualisms.

Bronstein, J. L. and S. K. Richman (2015). "Active pollinator choice by Heliconia 'fits the bill'." Trends Plant Sci **20**(7): 403-404.

A new study documents that a tropical plant only reproduces when pollen has been deposited by a visitor capable of extracting nectar from its deep flowers. Large, long-billed hummingbirds generally carry greater quantities of, and more genetically diverse, pollen. Thus, plants can exert more active partner choice than previously considered possible.

Bronstein, J. L., et al. (2003). "Ecological Dynamics of Mutualist/Antagonist Communities." The American Naturalist **162**: S24-S39.

One approach to understanding how mutualisms function in community settings is to model well-studied pairwise interactions in the presence of the few species with which they interact most strongly. In nature, such species are often specialized antagonists of one or both mutualists. Hence, these models can also shed light on the problem of when and how mutualisms are able to persist in the face of exploitation. We used spatial stochastic simulations to model the ecological dynamics of obligate, species-specific mutualisms between plants and pollinating seed parasite insects (e.g., yuccas and yucca moths) in the presence of one of two obligate antagonist species: flower-feeding insects (florivores) or insects that parasitize seeds but fail to pollinate (exploiters). Our results suggest that mutualisms can persist surprisingly well in the presence of highly specialized antagonists but that they exhibit distinctly different temporal and spatial dynamics when antagonists are present. In our models, antagonists tend to induce oscillations in the mutualist populations. As the number of per capita visits by antagonists increase, the system’s oscillatory dynamics become more extreme, finally leading to the extinction of one or more of the three species. When the antagonists exhibit high per capita visitation frequencies and long dispersal distances, significant spatial patchiness emerges within these tripartite interactions. We found surprisingly little difference between the ecological effects of florivores and exploiters, although in general florivores tended to drive themselves (and sometimes the mutualists) to extinction at parameter values at which the exploiters were able to persist. These theoretical results suggest several testable hypotheses regarding the ecological and evolutionary persistence ofmutualisms. More broadly, they point to the critical importance of studying the dynamics of pairwise interactions in community contexts.

Campbell, D. R., et al. (2010). "Flower color influences insect visitation in alpine New Zealand." Ecology **91**(9): 2638–2649.

Despite a long-standing belief that insect pollinators can select for certainflower colors, there are few experimental demonstrations that free-flying insects choose between natural flowers based on color. We investigated responses of insect visitors to experimental manipulations of flower color in the New Zealand alpine. Native syrphid flies (Allograpta and Platycheirus) and solitary bees (Hylaeus and Leioproctus) showed distinct preferences for visiting certain flower species. These responses were determined, in part, by flower color, as insects also responded to experimental manipulations of visible petal color in 7 out of 11 tests with different combinations of flower species and insect type. When preferences were detected, syrphid flies chose yellow over white petals regardless of flower species, whereas Hylaeus chose white over yellow Ourisia glandulosa. In some cases, the strength and direction of color preference depended on the context of other floral traits, in which case the response usually favored the familiar, normal combination of traits. Syrphid flies also visited in response to floral morphological traits but did not show preference based on UV reflectance. The unusually high preponderance of white flowers in the New Zealand alpine is not explained by complete generalization of flower color choice. Instead, the insect visitors show preferences based on color, including colors other than white, along with other floral traits. Furthermore, they can respond in complex ways to combinations of floral cues, suggesting that traits may act in nonadditive ways in determining pollinator visitation.

Caruso, C. M., et al. (2019). "A meta-analysis of the agents of selection on floral traits." Evolution **73**(1): 4-14.

Floral traits are hypothesized to evolve primarily in response to selection by pollinators. However, selection can also be mediated by other environmental factors. To understand the relative importance of pollinator-mediated selection and its variation among trait and pollinator types, we analyzed directional selection gradients on floral traits from experiments that manipulated the environment to identify agents of selection. Pollinator-mediated selection was stronger than selection by other biotic factors (e.g., herbivores), but similar in strength to selection by abiotic factors (e.g., soil water), providing partial support for the hypothesis that floral traits evolve primarily in response to pollinators. Pollinator-mediated selection was stronger on pollination efficiency traits than on other trait types, as expected if efficiency traits affect fitness via interactions with pollinators, but other trait types also affect fitness via other environmental factors. In addition to varying among trait types, pollinator-mediated selection varied among pollinator taxa: selection was stronger when bees, long-tongued flies, or birds were the primary visitors than when the primary visitors were Lepidoptera or multiple animal taxa. Finally, reducing pollinator access to flowers had a relatively small effect on selection on floral traits, suggesting that anthropogenic declines in pollinator populations would initially have modest effects on floral evolution.

Chen, C., et al. (2009). "Private channel: a single unusual compound assures specific pollinator attraction inFicus semicordata." Functional Ecology **23**(5): 941-950.

1. Floral scents have been suggested to play a key role in the obligate pollination mutualism between figs and fig wasps. However, few studies have determined whether pollinator-attractive compounds could alone assure species-specificity (‘private channel’), or whether specificity is mediated by more complex ‘floral filters’, of which scent is only one component. 2. We examined changes in the floral volatile compounds of Ficus semicordata, a dioecious fig species, during and after pollination using headspace collection and compound identification by Gas Chromatography ⁄Mass Spectrometry (GC ⁄MS). One benzenoid compound, 4.methylanisole, was strongly predominant (94–98%) among the volatile compounds emitted by both male and female receptive figs of F. semicordata, whereas it was totally absent in the volatiles emitted by figs 4 days after pollination, as well as in receptive-stage volatiles emitted by two other sympatric fig species, Ficus racemosa and Ficus hispida. 3. Bioassays using the specific pollinator of F. semicordata, Ceratosolen gravelyi, in a Y-tube olfactometer showed that 4-methylanisole was attractive to C. gravelyi in a wide range of concentrations (from 1Æ22 · 10)2 ng ⁄100 lLto1Æ22 · 106ng ⁄100lL). Moreover, chemical blends lacking 4-methylanisole were unattractive to C. gravelyi. These non-active odour sources included volatile compounds emitted by receptive figs of the two other sympatric fig species and volatiles of F. semicordata post-pollination figs. 4. All these results suggest that 4-methylanisole is the main signal compound in the floral scent of F. semicordata that attracts its obligate pollinator to the host figs at the precise stage required for pollination and oviposition. Furthermore, the high proportion of 4-methylanisole in the odours of receptive figs of both sexes was consistent with the hypothesis of chemical mimicry in dioecious figs. 5. A simple signal comprised of one compound that is unusual among Ficus and that is an infrequent, usually minor, component of other floral odours, may thus function as a private channel in this specialized obligate mutualism.

Cozzolino, S., et al. (2015). "Herbivory Increases Fruit Set in Silene latifolia: A Consequence of Induced Pollinator-Attracting Floral Volatiles?" Journal of Chemical Ecology **41**(7): 622-630.

Although the effect of herbivory on plant reproduction has been investigated in some detail, little is known about how herbivores affect floral signalling. Here, we investigated the effect of foliar herbivory by the African Cotton Leafworm (Spodoptera littoralis) on floral signalling and fruit set in the White Campion (Silene latifolia). We found no effects of herbivory on floral traits involved in visual signalling (flower number, corolla diameter, calyx length, petal length) or in amount of nectar produced. However, Spodoptera-infested plants emitted higher amounts of the two floral volatiles, (Z)-3-hexenyl acetate and beta-ocimene, than control plants. Open pollinated, infested plants also were found to produce more fruits than control plants, but only with nocturnal pollinators. Experimental addition of the two induced floral volatiles to non-infested Silene flowers also led to the production of more fruits with nocturnal pollination. This suggests that higher fruit production in herbivore-infested plants was caused by increased nocturnal pollinator attraction, mediated by the induced floral emission of these two volatiles. Our results show that the effects of herbivory on plant reproductive success are not necessarily detrimental, as plants can compensate herbivory with increased investment in pollinator attraction.

Dannon, E. A., et al. (2010). "Effects of volatiles from Maruca vitrata larvae and caterpillar-infested flowers of their host plant Vigna unguiculata on the foraging behavior of the parasitoid Apanteles taragamae." Journal of Chemical Ecology **36**(10): 1083-1091.

The parasitoid wasp Apanteles taragamae is a promising candidate for the biological control of the legume pod borer Maruca vitrata, which recently has been introduced into Benin. The effects of volatiles from cowpea and peabush flowers and Maruca vitrata larvae on host selection behavior of the parasitoid Apanteles taragamae were investigated under laboratory conditions by using a Y-tube olfactometer. Naive and oviposition-experienced female wasps were given a choice between several odor sources that included (1) uninfested, (2) Maruca vitrata-infested, and (3) mechanically damaged cowpea flowers, as well as (4) stem portions of peabush plants carrying leaves and flowers, (5) healthy M. vitrata larvae, and moribund (6), and live (7) virus-infected M. vitrata larvae. Responses of naive and oviposition-experienced female wasps did not differ for any of the odor source combinations. Wasps were significantly attracted to floral volatiles produced by cowpea flowers that had been infested with M. vitrata larvae and from which the larvae had been removed. Apanteles taragamae females also were attracted to Maruca vitrata-infested flowers after removal of both the larvae and their feces. Female wasps discriminated between volatiles from previously infested flowers and mechanically damaged flowers. Uninfested cowpea flowers attracted only oviposition-experienced wasps that had received a rewarding experience (i.e. the parasitization of two M. vitrata larvae feeding on cowpea flowers) before the olfactometer test. Wasps also were attracted to uninfested leaves and flowers of peabush. Moreover, they were also attracted to healthy and live virus-infected M. vitrata larvae, but not when the latter were moribund. Our data show that, similarly to what has been extensively been reported for foliar volatiles, flowers of plants also emit parasitoid-attracting volatiles in response to being infested with an herbivore.

Dellinger, A. S. (2020). "Pollination syndromes in the 21(st) century: where do we stand and where may we go?" New Phytol **228**(4): 1193-1213.

Pollination syndromes, recurring suites of floral traits appearing in connection with specific functional pollinator groups, have served for decades to organise floral diversity under a functional-ecological perspective. Some potential caveats, such as over-simplification of complex plant-animal interactions or lack of empirical observations, have been identified and discussed in recent years. Which of these caveats do indeed cause problems, which have been solved and where do future possibilities lie? I address these questions in a review of the pollination-syndrome literature of 2010 to 2019. I show that the majority of studies was based on detailed empirical pollinator observations and could reliably predict pollinators based on a few floral traits such as colour, shape or reward. Some traits (i.e. colour) were less reliable in predicting pollinators than others (i.e. reward, corolla width), however. I stress that future studies should consider floral traits beyond those traditionally recorded to expand our understanding of mechanisms of floral evolution. I discuss statistical methods suitable for objectively analysing the interplay of system-specific evolutionary constraints, pollinator-mediated selection and adaptive trade-offs at microecological and macroecological scales. I exemplify my arguments on an empirical dataset of floral traits of a neotropical plant radiation in the family Melastomataceae.

Dicke, M. (2000). "Chemical ecology of host-plant selection by herbivorous arthropods: a multitrophic perspective." Biochemical Systematics and Ecology **28**(7): 601-617.

Most herbivorous arthropods are specialists that feed on one or a few related plant species.To understand why this is so, both mechanistic and functional studies have been carried out, predominantly restricted to bitrophic aspects. Host-selection behaviour of herbivorous arthropods has been intensively studied and this has provided ample evidence for the role of secondary plant chemicals as source of information in behavioural decisions of herbivores. Many evolutionary studies have regarded co-evolution between plants and herbivores to explain the diversity of secondary plant chemicals and host specialisation of herbivores. However, many cases remain unexplained where herbivores select host plants that are suboptimal in terms of"tness returns. A stimulating paper by Bernays and Graham [(1988) Ecology 69, 886}892)] has initiated a discussion on the need of a multitrophic perspective to understand the evolution of host-plant specialisation by herbivorous arthropods. However, this has hardly resulted in ecological studies on host-selection behaviour that take a multitrophic perspective. Yet, evidence is accumulating that constitutive and induced infochemicals from natural enemies and competitors can a!ect herbivore behaviour. These cues may constitute important information on "tness prospects, just as plant cues can do. In this paper I selectively review how information from organisms at di!erent trophic levels varies in space and time and how herbivores can integratively exploit this information during host selection. In doing so, research areas are identi"ed that are likely to provide important new insights to explain several of the questions in herbivore host selection that remain unanswered so far. These research areas are at the interface of evolutionary ecology, behavioural ecology and chemical ecology.

Dotterl, S., et al. (2005). "Qualitative and quantitative analyses of flower scent in Silene latifolia." Phytochemistry **66**(2): 203-213.

The quantitative and qualitative variability in floral scent of 98 specimens of the dioecious species Silene latifolia belonging to 15 European and 19 North American populations was determined. Floral scent was collected from single flowers using dynamic headspace methods, and analysed by Micro-SPE and GC-MS methods. The flowers showed a nocturnal rhythm, and scent was emitted only at night. The amount of emitted volatiles varied greatly during the season, from 400 ng/flower/2 min in June to 50 ng/flower/2 min in August and September. The qualitative variability in the floral scent was high and different chemotypes, characterised by specific scent compounds, were found. Female and male flowers emitted the same type and amount of volatiles. The differences in floral scent composition between European and North American populations were small. Typical compounds were isoprenoids like lilac aldehyde isomers, or trans-beta-ocimene, and benzenoids like benzaldehyde, phenyl acetaldehyde, or veratrole. Some of these compounds are known to attract nocturnal Lepidoptera species. The high qualitative variability is discussed in relation to the pollination biology of S. latifolia, and the results are compared with other studies investigating intraspecific variability of flower scent.

Dudareva, N., et al. (2013). "Biosynthesis, function and metabolic engineering of plant volatile organic compounds." New Phytologist **198**(1): 16-32.

Plants synthesize an amazing diversity of volatile organic compounds (VOCs) that facilitate interactions with their environment, from attracting pollinators and seed dispersers to protecting themselves from pathogens, parasites and herbivores. Recent progress in -omics technologies resulted in the isolation of genes encoding enzymes responsible for the biosynthesis of many volatiles and contributed to our understanding of regulatory mechanisms involved in VOC formation. In this review, we largely focus on the biosynthesis and regulation of plant volatiles, the involvement of floral volatiles in plant reproduction as well as their contribution to plant biodiversity and applications in agriculture via crop-pollinator interactions. In addition, metabolic engineering approaches for both the improvement of plant defense and pollinator attraction are discussed in light of methodological constraints and ecological complications that limit the transition of crops with modified volatile profiles from research laboratories to real-world implementation.

Elzing, J. A., et al. (2007). "Time after time: flowering phenology and biotic interactions." Trends in Ecology & Evolution **22**(8): 432-439.

Fenster, C. B., et al. (2004). "Pollination Syndromes and Floral Specialization." Annual Review of Ecology, Evolution, and Systematics **35**(1): 375-403.

Floral evolution has often been associated with differences in pollination syndromes. Recently, this conceptual structure has been criticized on the grounds that flowers attract a broader spectrum of visitors than one might expect based on their syndromes and that flowers often diverge without excluding one type of pollinator in favorof another. Despite these criticisms, we show that pollination syndromes provide great utility in understanding the mechanisms of floral diversification. Our conclusions are based on the importance of organizing pollinators into functional groups according to presumed similarities in the selection pressures they exert. Furthermore, functional groups vary widely in their effectiveness as pollinators for particular plant species. Thus, although a plant may be visited by several functional groups, the relative selective pressures they exert will likely be very different. We discuss various methods of documenting selection on floral traits. Our review of the literature indicates overwhelming evidence that functional groups exert different selection pressures on floral traits. We also discuss the gaps in our knowledge of the mechanisms that underlie the evolution of pollination syndromes. In particular, we need more information about the relative importance of specific traits in pollination shifts, about what selective factors favor shifts between functional groups, about whether selection acts on traits independently or in combination, and about the role of history in pollination-syndrome evolution.

Fineblum, W. L. and M. D. Rausher (1997). "Do Floral Pigmentation Genes Also Influence Resistance to Enemies? The W Locus in Ipomoea Purpurea." Ecology **78**(6): 1646-1654.

Biochemical pathways that produce floral pigments often also produce secondary compounds that are believed to protect plants from natural enemies. Mutations that affect floral pigment patterns are thus also expected to influence the production of compounds conferring resistance to natural enemies, suggesting that the evolution of floral pigment patterns may be guided not just by interactions with pollinators, but also by indirect selection exerted by enemies on resistance. In particular, mutations that block production of floral pigments may be expected either to block production of defensive compounds or, alternatively, to lead to increased production of defenses, depending on the position of the blockage in the pathway. This expectation was examined in the morning glory Ipomoea purpurea by assessing whether white-flowered genotypes are more or less susceptible to attack by natural enemies than pigmented genotypes. No such difference in susceptibility was detected, although resistance to capsule-feeding insects exhibited a pigment genotype X background genotype interaction. While this negative result indicates that natural enemies probably do not influence the evolution of floral pigment pattern in L purpurea, this remains a possibility in other systems.

Galen, C. (1999). "Why Do Flowers Vary?The functional ecology of variation in flower size and form within natural plant populations." Bioscience **49**(8): 631-640.

Galen, C. and B. Butchart (2003). "Ants in your plants: effects of nectar-thieves on pollen fertility and seed-siring capacity in the alpine wildflower, Polemonium Uiscosum." OIKOS **101**(3): 521-528.

Flowers of the alpine skypilot, Polemonium Tiscosum, are attacked by nectar thieving ants of Formica neorufibarbus gelida. Ants exert selection on flower scent, size and shape in skypilots by damaging the pistils. Here, I report on the frequency and nature of contact between ants and pollen-bearing anthers and determine the consequences of such contact for pollen performance and pollen donor paternity. In laboratory trials, ants entered flowers with full intact anthers and emasculated (female) flowers equivalently. Similarly, flower visitation rates of ants foraging naturally were not affected by the frequency of male phase flowers per plant. Ants actively interacted with the pollen-bearing anthers during 21% of flower visits, on average. The rate at which such interactions occurred was predicted by the proportion of flowers in the male phase, under a random foraging model. The effect of ants on pollen fertility was tested experimentally by enclosing ants in male-phase flowers on intact inflorescences. Adjacent control flowers were left un-occupied. Pollen from flowers with a history of ant occupancy had significantly lower germination on virgin recipient stigmas than pollen from unoccupied control flowers. With hand-pollination, sufficient pollen was transferred from ant-occupied flowers to saturate seed set. However, a model based on the relationship between seed set and compatible pollen delivery by natural pollinators indicated that ant damage to pollen should reduce paternity accruing per flower visit by 20–26% on average, in nature. Results support the hypothesis that in P. Tiscosum, selection on floral traits by nectar-thieving ants operates through male as well as female function.

Gaudinier, A. and B. K. Blackman (2020). "Evolutionary processes from the perspective of flowering time diversity." New Phytologist **225**(5): 1883-1898.

Although it is well appreciated that genetic studies of flowering time regulation have led to fundamental advances in the fields of molecular and developmental biology, the ways in which genetic studies of flowering time diversity have enriched the field of evolutionary biology have received less attention despite often being equally profound. Because flowering time is a complex, environmentally responsive trait that has critical impacts on plant fitness, crop yield, and reproductive isolation, research into the genetic architecture and molecular basis of its evolution continues to yield novel insights into our understanding of domestication, adaptation, and speciation. For instance, recent studies of flowering time variation have reconstructed how, when, and where polygenic evolution of phenotypic plasticity proceeded from standing variation and de novo mutations; shown how antagonistic pleiotropy and temporally varying selection maintain polymorphisms in natural populations; and provided important case studies of how assortative mating can evolve and facilitate speciation with gene flow. In addition, functional studies have built detailed regulatory networks for this trait in diverse taxa, leading to new knowledge about how and why developmental pathways are rewired and elaborated through evolutionary time.

Gélvez-Zúñiga, I., et al. (2018). "Floral antagonists counteract pollinator-mediated selection on attractiveness traits in the hummingbird-pollinated Collaea cipoensis (Fabaceae)." Biotropica.

Pollinator-mediated selection toward larger and abundant flowers is common in naturally pollen-limited populations. However, floral antagonists may counteract this effect, maintaining smaller- and few-flowered individuals within populations. We quantified pollinator and antagonist visit rates and determined a multiplicative female fitness component from attacked and non-attacked flowers of the Brazilian hummingbird-pollinated shrub Collaea cipoensis to determine the selective effects of pollinators and floral antagonists on flower size and number. We predicted that floral antagonists reduce the female fitness component and thus exert negative selective pressures on flower size and number, counteracting the positive effects of pollinators. Pollinators, mainly hummingbirds, comprised 4% of total floral visitation, whereas antagonist ants and bees accounted for 90% of visitation. Nectar-robbers involved about 99% of floral antagonist visit rates, whereas florivores comprised the remaining 1%. Larger and abundant flowers increased both pollinator and antagonist visit rates and the female fitness component significantly decreased in flowers attacked by nectar-robbers and florivores in comparison to non-attacked flowers. We detected that pollinators favored larger- and many-flowered individuals, whereas floral antagonists exerted negative selection on flower size and number. This study confirms that floral antagonists reduce female plant fitness and this pattern directly exerts negative selective pressures on flower size and number, counteracting pollinator-mediated selection on floral attractiveness traits.

Gigord, L. D., et al. (2002). "The potential for floral mimicry in rewardless orchids: an experimental study." Proceedings of the Royal Society B: Biological Sciences **269**(1498): 1389-1395.

More than one-third of orchid species do not provide their pollinators with either pollen or nectar rewards. Floral mimicry could explain the maintenance of these rewardless orchid species, but most rewardless orchids do not appear to have a rewarding plant that they mimic specifically. We tested the hypothesis that floral mimicry can occur through similarity based on corolla colour alone, using naive bumble-bees foraging on arrays of plants with one rewarding model species, and one rewardless putative mimic species (Dactylorhiza sambucina) which had two colour morphs. We found that when bees were inexperienced, they visited both rewardless morphs randomly. However, after bees had gained experience with the rewarding model, and it was removed from the experiment, bees resampled preferentially the rewardless morph most similar to it in corolla colour. This is the first clear evidence, to our knowledge, that pollinators could select for floral mimicry. We suggest that floral mimicry can be a selective force acting on rewardless orchids, but only under some ecological conditions. In particular, we argue that selection on early-flowering rewardless orchids that receive visits from a large pool of naive pollinators will be weakly influenced by mimicry.

Giurfa, M., et al. (1994). "Odour and colour information in the foraging choice behaviour of the honeybee." Journal of Comparative Physiology A **175**(6).

Flower-naive honeybees Apis mellifera L. flying in an enclosure were tested for their colour preferences. Bees were rewarded once on an achromatic (grey, aluminium or hardboard), or on a chromatic (ultraviolet) disk. Since naive bees never alighted on colour stimuli alone, a scent was given in combination with colour. Their landings on twelve colour stimuli were recorded. Results after one reward ("first test") were analysed separately from those obtained after few rewards ("late tests"). 1) After pre-training to achromatic signals, bees preferred, in the first test, bee-uv-blue and bee-green colours. With increasing experience, the original preference pattern persisted but the choice of bee-blue and bee-green colours increased. 2) Neither colour distance of the test stimuli to the background or to the pre-training signal, nor their intensity, nor their green contrast, accounted for the colour choice of bees. Choices reflected innate preferences and were only associated with stimulus hue. 3) Bees learned very quickly the pre-trained chromatic stimulus, the original colour preferences being thus erased. 4) Colour preferences were strongly correlated with flower colour and its associated nectar reward, as measured in 154 flower species. 5) Colour preferences also resemble the wavelength dependence of colour learning demonstrated in experienced bees.

Gómez, J. M., et al. (2008). "Association between floral traits and rewards in Erysimum mediohispanicum (Brassicaceae)." Annals of Botany **101**(9): 1413-1420.

BACKGROUND AND AIMS: Floral rewards may be associated with certain morphological floral traits and thus act as underlying factors promoting selection on these traits. This study investigates whether some traits that are under pollinator-mediated selection (flower number, stalk height, corolla diameter, corolla tube length and corolla tube width) in the Mediterranean herb E. mediohispanicum (Brassicaceae) are associated with rewards (pollen and nectar). METHODS: During 2005 the phenotypic traits and the visitation rate of the main pollinator functional groups were quantified in 720 plants belonging to eight populations in south-east Spain, and during 2006 the same phenotypic traits and the reward production were quantified in 400 additional plants from the same populations. KEY RESULTS: A significant correlation was found between nectar production rate and corolla tube length, and between pollen production and corolla diameter. Visitation rates of large bees and butterflies were significantly higher in plants exhibiting larger flowers with longer corolla tubes. CONCLUSIONS: The association between reward production and floral traits may be a factor underlying the pattern of visitation rate displayed by some pollinators.

Gómez, J. M., et al. (2008). "Spatial variation in selection on corolla shape in a generalist plant is promoted by the preference patterns of its local pollinators." Proceedings of the Royal Society B: Biological Sciences **275**(1648): 2241-2249.

An adaptive role of corolla shape has been often asserted without an empirical demonstration of how natural selection acts on this trait. In generalist plants, in which flowers are visited by diverse pollinator fauna that commonly vary spatially, detecting pollinator-mediated selection on corolla shape is even more difficult. In this study, we explore the mechanisms promoting selection on corolla shape in the generalist crucifer Erysimum mediohispanicum Polatschek (Brassicaceae). We found that the main pollinators of E. mediohispanicum (large bees, small bees and bee flies) discriminate between different corolla shapes when offered artificial flowers without reward. Importantly, different pollinators prefer different shapes: bees prefer flowers with narrow petals, whereas bee flies prefer flowers with rounded overlapping petals. We also found that flowers with narrow petals (those preferred by bees) produce both more pollen and nectar than those with rounded petals. Finally, different plant populations were visited by different faunas. As a result, we found spatial variation in the selection acting on corolla shape. Selection favoured flowers with narrow petals in the populations where large or small bees are the most abundant pollinator groups. Our study suggests that pollinators, by preferring flowers with high reward, exert strong selection on the E. mediohispanicum corolla shape. The geographical variation in the pollinator-mediated selection on E. mediohispanicum corolla shape suggests that phenotypic evolution and diversification can occur in this complex floral trait even without specialization.

Gómez, J. M., et al. (2006). "Natural Selection on Erysimum mediohispanicum Flower Shape: Insights into the Evolution of Zygomorphy." American Naturalist **168**(4): 531-545.

Paleontological and phylogenetic studies have shown that floral zygomorphy (bilateral symmetry) has evolved independently in several plant groups from actinomorphic (radially symmetric) ancestors as a consequence of strong selection exerted by specialized pollinators. Most studies focused on unraveling the developmental genetics of flower symmetry, but little is known about the adaptive significance ofintraspecific flower shape variation under natural conditions. We provide the first evidence for natural selection favoring zygomorphy in a wild population of Erysimum mediohispanicum (Brassicaceae), a plant showing extensive continuous variation in flower shape, ranging from actinomorphic to zygomorphic flowers. By using geometric morphometric tools to describe flower shape, we demonstrate that plants bearing zygomorphic flowers received more pollinator visits and had the highest fitness, measured not only by the number of seeds produced per plant but also by the number of seeds surviving to the juvenile stage. This study provides strong evidence for the existence of significant fitness differences associated with floral shape variation in E. mediohispanicum, thus illuminating a pathway for the evolution of zygomorphy in natural populations.

Gómez, J. M. a. and F. Perfectti (2010). "Evolution of complex traits: the case of Erysimum corolla shape." International Journal of Plant Sciences **171**(9): 987–998.

The evolution of flower shape has attracted the attention of biologists for at least two hundred years. Although much information is accumulating on the genetic architecture of flower shape, information on its adaptive significance is much scarcer. Using geometric morphometrics, we have explored the microevolution of corolla shape in Erysimum mediohispanicum during the past decade.We have found that, by contrast with conventional wisdom, corolla shape shows great variation even between co-occurring individuals. This variation can have strong fitness consequences, with reproductive success being associated with specific corolla shapes. Corolla shape seems to act in E. mediohispanicum as an honest signal, since it is associated with reward (nectar and pollen) and determines the preference pattern of important pollinators. Finally, since pollinator fauna varies geographically in this generalist plant species, we have detected a geographic mosaic of selection on E. mediohispanicum corolla shape in southeastern Spain that has resulted in a pattern of local adaptation.We hope that this review will encourage other evolutionary biologists to explore corolla shape microevolution, helping to unravel Darwin’s ‘‘abominable mystery.’’

Gorden, N. L. S. and L. S. Adler (2016). "Florivory shapes both leaf and floral interactions." Ecosphere **7**(6).

Florivory, or the consumption of flowers, is a ubiquitous interaction that can reduce plant reproduction directly by damaging reproductive tissues and indirectly by deterring pollinators. However, we know surprisingly little about how florivory alters plant traits or the larger community of species interactions. Although leaf damage is known to affect floral traits and interactions in many systems, the consequences of floral damage for leaf traits and interactions are unknown. We manipulated floral damage in Impatiens capensis and measured effects on floral attractive traits and secondary chemicals, leaf secondary chemicals, floral interactions, leaf herbivory, and plant reproduction. We also examined relationships between early season floral traits and floral interactions, to explore which traits structure floral interactions. Moderate but not high florivory significantly increased relative selfed reproduction, leading to a shift in mating system away from outcrossing. Florivory increased leaf secondary compounds and decreased leaf herbivory, although mechanisms other than leaf chemistry may be responsible for some of the reduced leaf damage. Florivory altered four of seven measured interactions, including increased subsequent florivory and reduced flower spiders, although only leaf damage effects were significant after correcting for multiple tests. Pretreatment concentrations of floral anthocyanins and condensed tannins were associated with reduced levels of many floral antagonisms, including florivory, nectar larceny, and flower spider abundance, suggesting these traits play a role in floral resistance. Overall, our results indicate a broad range of community and potential evolutionary consequences of florivory through structuring subsequent floral interactions, altering leaf secondary chemicals, and shaping leaf herbivory.

Guimarães, P. R. (2020). "The Structure of Ecological Networks Across Levels of Organization." Annual Review of Ecology, Evolution, and Systematics **51**(1): 433-460.

Interactions connect the units of ecological systems, forming networks. Individual-based networks characterize variation in niches among individuals within populations. These individual-based networks merge with each other, forming species-based networks and food webs that describe the architecture of ecological communities. Networks at broader spatiotemporal scales portray the structure of ecological interactions across landscapes and over macroevolutionary time. Here, I review the patterns observed in ecological networks across multiple levels of biological organization. A fundamental challenge is to understand the amount of interdependence as we move from individual-based networks to species-based networks and beyond. Despite the uneven distribution of studies, regularities in network structure emerge across scales due to the fundamental architectural patterns shared by complex networks and the interplay between traits and numerical effects. I illustrate the integration ofthese organizational scales by exploring the consequences ofthe emergence ofhighly connected species for network structures across scales.

Hagler (1990). "Honey bee (Apis mellifera L) response to simulated onion nectars containing variable sugar and potassium concentrations." Apidologie **21**(2): 115-121.

Potassium concentrations in nectars of several common onion (Allium cepa L) cultivars were analyzed by atomic absorption spectrophotometry. Concentrations ranged from 5 347 to6 914 ppm. Honey bee (Apis mellifera L) collection of artificial nectars containing potassium, glucose, fructose, and sucrose concentrations, similar to several common onion cultivars, was evaluated quantitatively. Bees ingested greater volumes of nectar containing low potassium content. When simulated nectars had approximately equal concentrations of potassium, bees preferred nectar with higher carbohydrate content.

Hanley, M. E., et al. (2009). "Pollination and plant defence traits co-vary in Western Australian Hakeas." New Phytol **182**(1): 251-260.

* Despite the conflicting demands imposed by mutualistic (pollination) and antagonistic (florivory) processes, the relative importance of the key selective pressures influencing floral evolution are not readily apparent. In this study we quantified a range of floral and foliar traits within the genus Hakea to investigate how pollinator and herbivore selection might influence the evolution of floral attraction and defence attributes. * Plant material was collected from populations of 51 Australian Hakea species native to southwestern Australia, and measurements were taken of foliage and inflorescence morphology, inflorescence colour and floral chemical defence. Hakeas were separated into bird- vs insect-pollinated species on the basis of stigma-nectary distance. * Our results show how the evolution of insect vs bird pollination is closely linked to whether inflorescences are protected by physical (leaf spines, dense foliage) or chemical (floral cyanide) defences, respectively. * Rather than being constrained by the necessity to attract pollinators, we suggest that pre-existing adaptations to combat florivore and herbivore attack directed the evolution of floral characteristics employed to attract pollinators and deter florivores. The inter-correlation among bird pollination, red flower colour and floral cyanide indicates floral coloration may signal to vertebrate florivores that the inflorescences are unpalatable despite their high accessibility.

Harder, L. D. and S. D. Johnson (2005). "Adaptive plasticity of floral display size in animal-pollinated plants." Proc Biol Sci **272**(1581): 2651-2657.

Plants need not participate passively in their own mating, despite their immobility and reliance on pollen vectors. Instead, plants may respond to their recent pollination experience by adjusting the number of flowers that they display simultaneously. Such responsiveness could arise from the dependence of floral display size on the longevity of individual flowers, which varies with pollination rate in many plant species. By hand-pollinating some inflorescences, but not others, we demonstrate plasticity in display size of the orchid Satyrium longicauda. Pollination induced flower wilting, but did not affect the opening of new flowers, so that within a few days pollinated inflorescences displayed fewer flowers than unpollinated inflorescences. During subsequent exposure to intensive natural pollination, pollen removal and receipt increased proportionally with increasing display size, whereas pollen-removal failure and self-pollination accelerated. Such benefit-cost relations allow plants that adjust display size in response to the prevailing pollination rate to increase their attractiveness when pollinators are rare (large displays), or to limit mating costs when pollinators are abundant (small displays). Seen from this perspective, pollination-induced flower wilting serves the entire plant by allowing it to display the number of flowers that is appropriate for the current pollination environment.

Harder, L. D., et al. (2019). "The dynamic mosaic phenotypes of flowering plants." New Phytologist **224**: 1021–1034.

Ecological interaction and adaptation both depend on phenotypic characteristics. In contrast with the common conception of the ‘adult’ phenotype, plant bodies develop continuously during their lives. Furthermore, the different units (metamers) that comprise plant bodies are not identical copies, but vary extensively within individuals. These characteristics foster recognition of plant phenotypes as dynamic mosaics. We elaborate this conception based largely on a wide-ranging review of developmental, ecological and evolutionary studies of plant reproduction, and identify its utility in the analysis of plant form, function and diversification.Anexpanded phenotypic conception is warranted because dynamic mosaic features affect plant performance and evolve. Evidence demonstrates that dynamic mosaic phenotypes enable functional ontogeny, division of labour, resource and mating efficiency. In addition, dynamic mosaic features differ between individuals and experience phenotypic selection. Investigation of the characteristics and roles of dynamic and mosaic features of plant phenotypes benefits from considering within-individual variation as a functionvalued trait that can be analysed with functional data methods. Phenotypic dynamics and within-individual variation arise despite an individual’s genetic uniformity, and develop largely by heterogeneous gene expression and associated hormonal control. These characteristics can be heritable, so that dynamic mosaic phenotypes can evolve and diversify by natural selection.

Haverkamp, A., et al. (2016). "Innate olfactory preferences for flowers matching proboscis length ensure optimal energy gain in a hawkmoth." Nature Communications **7**: 11644.

Cost efficient foraging is of especial importance for animals like hawkmoths or hummingbirds that are feeding 'on the wing', making their foraging energetically demanding. The economic decisions made by these animals have a strong influence on the plants they pollinate and floral volatiles are often guiding these decisions. Here we show that the hawkmoth Manduca sexta exhibits an innate preference for volatiles of those Nicotiana flowers, which match the length of the moth's proboscis. This preference becomes apparent already at the initial inflight encounter, with the odour plume. Free-flight respiration analyses combined with nectar calorimetry revealed a significant caloric gain per invested flight energy only for preferred-matching-flowers. Our data therefore support Darwin's initial hypothesis on the coevolution of flower length and moth proboscis. We demonstrate that this interaction is mediated by an adaptive and hardwired olfactory preference of the moth for flowers offering the highest net-energy reward.

Hegland, S. J., et al. (2009). "How does climate warming affect plant-pollinator interactions?" Ecology Letters **12**(2): 184-195.

Climate warming affects the phenology, local abundance and large-scale distribution of plants and pollinators. Despite this, there is still limited knowledge of how elevated temperatures affect plant-pollinator mutualisms and how changed availability of mutualistic partners influences the persistence of interacting species. Here we review the evidence of climate warming effects on plants and pollinators and discuss how their interactions may be affected by increased temperatures. The onset of flowering in plants and first appearance dates of pollinators in several cases appear to advance linearly in response to recent temperature increases. Phenological responses to climate warming may therefore occur at parallel magnitudes in plants and pollinators, although considerable variation in responses across species should be expected. Despite the overall similarities in responses, a few studies have shown that climate warming may generate temporal mismatches among the mutualistic partners. Mismatches in pollination interactions are still rarely explored and their demographic consequences are largely unknown. Studies on multi-species plant-pollinator assemblages indicate that the overall structure of pollination networks probably are robust against perturbations caused by climate warming. We suggest potential ways of studying warming-caused mismatches and their consequences for plant-pollinator interactions, and highlight the strengths and limitations of such approaches.

Heil, M., et al. (2001). "Extrafloral nectar production of the ant-associated plant, Macaranga tanarius, is an induced, indirect, defensive response elicited by jasmonic acid." PNAS **98**(3): 1083–1088.

Plant species in at least 66 families produce extrafloral nectar (EFN) on their leaves or shoots and therewith attract predators and parasitoids, such as ants and wasps, which in turn defend them against herbivores. We investigated whether EFN secretion is induced by herbivory and兾or artificial damage, and thus can be regarded as an induced defensive response. In addition, we studied the underlying signaling pathway. EFN secretion by field-grown Macaranga tanarius increased after herbivory, artificial leaf damage, and exogenous jasmonic acid (JA) application. Artificial damage strongly enhanced endogenous JA concentrations. The response in EFN production to artificial damage was much less pronounced in those leaves that were treated with phenidone to inhibit endogenous JA synthesis. Quantitative dose–response relations were found between the increase in nectar production and both the intensity of leaf damage and the amounts of exogenously applied JA. The amount of endogenously produced JA was positively correlated with the intensity of leaf damage. Increased numbers of defending insects and decreased numbers of herbivores were observed on leaves after inducing EFN production by exogenous JA treatment. Over 6 weeks, repeatedly applied JA or artificial damage resulted in a ten-fold reduction in herbivory. These results demonstrate that EFN production represents an alternative mechanism for induced, indirect defensive plant responses that are mediated via the octadecanoid signal transduction cascade.

Hempel de Ibarra, N., et al. (2015). "More than colour attraction: behavioural functions of flower patterns." Current Opinion In Insect Science **12**: 64-70.

Flower patterns are thought to influence foraging decisions of insect pollinators. However, the resolution of insect compound eyes is poor. Insects perceive flower patterns only from short distances when they initiate landings or search for reward on the flower. From further away flower displays jointly form larger-sized patterns within the visual scene that will guide the insect's flight. Chromatic and achromatic cues in such patterns may help insects to find, approach and learn rewarded locations in a flower patch, bringing them close enough to individual flowers. Flight trajectories and the spatial resolution of chromatic and achromatic vision in insects determine the effectiveness of floral displays, and both need to be considered in studies of plant-pollinator communication.

Hendry, A. P. and T. Day (2005). "Population structure attributable to reproductive time: isolation by time and adaptation by time." Molecular Ecology **14**(4): 901-916.

Many populations are composed of a mixture of individuals that reproduce at different times, and these times are often heritable. Under these conditions, gene flow should be limited between early and late reproducers, even within populations having a unimodal temporal distribution of reproductive activity. This temporal restriction on gene flow might be called "isolation by time" (IBT) to acknowledge its analogy with isolation by distance (IBD). IBD and IBT are not exactly equivalent, however, owing to differences between dispersal in space and dispersal in time. We review empirical studies of natural populations that provide evidence for IBT based on heritabilities of reproductive time and on molecular genetic differences associated with reproductive time. When IBT is present, variation in selection through the reproductive season may lead to adaptive temporal variation in phenotypic traits [adaptation by time (ABT)]. We introduce a novel theoretical model that shows how ABT increases as (i) selection on the trait increases; (ii) environmental influences on reproductive time decrease; (iii) the heritability of reproductive time increases; and (iv) the temporal distribution of reproductive activity becomes increasingly uniform. We then review empirical studies of natural populations that provide evidence for ABT by documenting adaptive temporal clines in phenotypic traits. The best evidence for IBT and ABT currently comes from salmonid fishes and flowering plants, but we expect that future work will show these processes are more widespread.

Inouye, D. W. (1980). "The Terminology of Floral Larceny." Ecology **61**(5): 1251-1253.

Irwin, R. E., et al. (2004). "The Dual Role of Floral Traits: Pollinator Attraction and Plant Defense." Ecology **85**(6): 1503–1511.

Plants are under siege from a diversity of enemies that consume both leaf and floral parts. Plants resist damage to leaves in a variety of ways, and we now have a rich literature documenting how plants defend themselves against herbivore attack. In contrast, the mechanisms by which plants resist enemies that consume floral parts or resources are much less known, even though damage to floral tissue usually has tighter links to plant fitness than damage to leaf tissue. Many plants experience nectar robbing, whereby floral visitors remove nectar from flowers, often without pollinating. Nectar robbers can reduce plant fitness to degrees comparable to, or even surpassing, reduction by herbivores. However, because nectar attracts both pollinators and nectar robbers, plants face a dilemma in defending against nectar robbers without also deterring pollinators. Here, we extend the conceptual framework of resistance to herbivores to include resistance to nectar robbers, focusing on nectar traits. We review published data and find that an array of nectar traits may deter robbers without deterring pollinators. Although resistance traits against robbers have been broadly identified, the costs and benefits of these traits in terms of plant fitness remain poorly understood. We present data showing that a nectar trait (dilute nectar) might directly, as well as indirectly, benefit plant fitness by deterring nectar-robbing bumble bees of Ipomopsis aggregata without deterring hummingbird pollinators. However, the magnitude of any plant fitness benefit will depend on the degree to which plants are pollen- vs. resource-limited in a given year. The results of our work offer both conceptual and empirical insight into how plants cope with attack by nonpollinating floral visitors through a relatively unexplored trait, nectar.

Irwin, R. E., et al. (2010). "Nectar Robbing: Ecological and Evolutionary Perspectives." Annual Review of Ecology, Evolution, and Systematics **41**(1): 271-292.

Not all floral visitors attracted to flowers are pollinators. Instead, some visitors circumvent the floral opening, usually removing nectar without contacting the anthers and/or stigma. Here we review the evolutionary ecology of nectar robbing from both the plant and animal perspective. Effects of robbing on female and male components of plant reproduction range from negative to positive. Their underlying mechanisms are diverse, including direct effects and indirect effects mediated through changes in pollination. We detail how plants may be able to deter robbers through morphological and chemical traits. For the evolutionary ecology of robbing to move beyond a phytocentric perspective, studies must also address the causes of robbing and the consequences for both robbers and pollinators. We use an energetics approach to evaluate these causes and consequences. Finally, we highlight unanswered questions in need of further research.

Johnson, S. D. and R. A. Raguso (2016). "The long-tongued hawkmoth pollinator niche for native and invasive plants in Africa." Ann Bot **117**(1): 25-36.

BACKGROUND AND AIMS: Unrelated organisms that share similar niches often exhibit patterns of convergent evolution in functional traits. Based on bimodal distributions of hawkmoth tongue lengths and tubular white flowers in Africa, this study hypothesized that long-tongued hawkmoths comprise a pollination niche (ecological opportunity) that is distinct from that of shorter-tongued hawkmoths. METHODS: Field observations, light trapping, camera surveillance and pollen load analysis were used to identify pollinators of plant species with very long-tubed (>8 cm) flowers. The nectar properties and spectral reflectance of these flowers were also measured. The frequency distributions of proboscis length for all captured hawkmoths and floral tube length for a representative sample of night-blooming plant species were determined. The geographical distributions of both native and introduced plant species with very long floral tubes were mapped. KEY RESULTS: The convolvulus hawkmoth Agrius convolvuli is identified as the most important pollinator of African plants with very long-tubed flowers. Plants pollinated by this hawkmoth species tend to have a very long (approx. 10 cm) and narrow flower tube or spur, white flowers and large volumes of dilute nectar. It is estimated that >70 grassland and savanna plant species in Africa belong to the Agrius pollination guild. In South Africa, at least 23 native species have very long floral tubes, and pollination by A. convolvuli or, rarely, by the closely related hawkmoth Coelonia fulvinotata, has been confirmed for 11 of these species. The guild is strikingly absent from the species-rich Cape floral region and now includes at least four non-native invasive species with long-tubed flowers that are pre-adapted for pollination by A. convolvuli. CONCLUSIONS: This study highlights the value of a niche perspective on pollination, which provides a framework for making predictions about the ecological importance of keystone pollinators, and for understanding patterns of convergent evolution and the role of floral traits in plant colonization.

Joly, S., et al. (2018). "Greater pollination generalization is not associated with reduced constraints on corolla shape in Antillean plants." Evolution **72**(2): 244-260.

Flowers show important structural variation as reproductive organs but the evolutionary forces underlying this diversity are still poorly understood. In animal-pollinated species, flower shape is strongly fashioned by selection imposed by pollinators, which is expected to vary according to guilds of effective pollinators. Using the Antillean subtribe Gesneriinae (Gesneriaceae), we tested the hypothesis that pollination specialists pollinated by one functional type of pollinator have maintained more similar corolla shapes through time due to more constant and stronger selection constraints compared to species with more generalist pollination strategies. Using geometric morphometrics and evolutionary models, we showed that the corolla of hummingbird specialists, bat specialists, and species with a mixed-pollination strategy (pollinated by hummingbirds and bats; thus a more generalist strategy) have distinct shapes and that these shapes have evolved under evolutionary constraints. However, we did not find support for greater disparity in corolla shape of more generalist species. This could be because the corolla shape of more generalist species in subtribe Gesneriinae, which has evolved multiple times, is finely adapted to be effectively pollinated by both bats and hummingbirds. These results suggest that ecological generalization is not necessarily associated with relaxed selection constraints.

Jönsson, M. and P. Anderson (2007). "Emission of oilseed rape volatiles after pollen beetle infestation; behavioural and electrophysiological responses in the parasitoid Phradis morionellus." Chemoecology **17**(4): 201-207.

The pollen beetle, Meligethes aeneus, is an important pest of oilseed rape, Brassica napus. Larvae of this species feed only in the buds and flowers of Brassicaceae. One important natural enemy of this beetle is the parasitoid Phradis morionellus that attacks larvae in buds and flowers and also feeds on the flowers. The preferences for odours of non-infested and infested rape were tested for both starved and fed parasitoids in Y-tube olfactometer experiments. The volatile blend released from pollen beetle-infested and non-infested flowering rape and from pollen beetle larvae was identified and quantified. Gas chromatography-electroantennodetection analyses were performed with female P. morionellus. Parasitoids in both treatment groups preferred infested rape, but the proportion of responding female P. morionellus was significantly lower for the group that was starved. Six of the 20 volatiles identified were released at higher rates from infested rape than from non-infested. None of these compounds was found in pollen beetle larvae headspace. P. morionellus antennae detected both major and minor components in the volatile blend. The volatiles released at a significantly higher rate from infested rape and detected by P. morionellus antennae were (Z)-3-hexenylacetate, (Z)-3-hexenol, 3-butenyl isothiocyanate and (E,E)-α-farnesene.

Jurgens, A., et al. (2006). "The chemical nature of fetid floral odours in stapeliads (Apocynaceae-Asclepiadoideae-Ceropegieae)." New Phytologist **172**(3): 452-468.

By emitting strong fetid scents, sapromyiophilous flowers mimic brood and food sites of flies to attract them as pollinators. To date, intensive comparative scent analyses have been restricted to sapromyiophilous Araceae. Here, we analysed flower volatiles of fetid stapeliads to improve our understanding of the floral biology of fly pollinated species, and to learn whether mimicry types comparable to those found in Araceae exist. Floral volatiles of 15 species out of 11 genera within the Asclepiadoideae-Ceropegieae-Stapeliinae were collected via headspace adsorption and thermal desorption and analysed by gas chromatography-mass spectometry (GC-MS). Data were analysed using CNESS-NMDS statistics. Sapromyiophilous stapeliads are highly diverse in their scent composition, in which sulphur compounds, benzenoids, fatty acid derivatives or nitrogen-containing compounds dominate. Four groups are evident: species with high p-cresol content but low amounts of polysulphides (herbivore faeces mimicry); species with mainly polysulphides and low amounts of p-cresol (carnivore/omnivore faeces or carcass mimicry); species with high amounts of heptanal and octanal (carnivore/omnivore faeces or carcass mimicry); and species with hexanoic acid (urine mimicry). Considering the findings in the unrelated Araceae, our results support the universality of different mimicry types that are obviously subsumed under the sapromyiophilous syndrome.

Kaczorowski, R. L., et al. (2012). "Corolla shape vs. size in flower choice by a nocturnal hawkmoth pollinator." Functional Ecology **26**(3): 577-587.

1. Corolla shape is one of many floral traits used by pollinators when making foraging decisions. Corolla shape has been implicated as a factor in floral isolation and evolutionary shifts between pollinator affinities, yet few studies have investigated how quantifiable differences in corolla shape affect pollinator behaviour. 2. This study measured variation in corolla shape (primarily corolla limb dissectedness) across eight species of Nicotiana (section Alatae) and its influence on flower choice by nocturnal hawkmoths (Manduca sexta). Behavioural assays performed under simulated moonlight tested for hawkmoth preference and performance differences between the average floral shape of sphingophilous species (CHawkmoth), the average of all Nicotiana species in this study (CAll), and ⁄or the average ofhummingbird-pollinated species (CHummingbird). 3. In mixed arrays, hawkmoths preferred CHawkmoth over CAll flowers, but only when shapes were not standardized for surface area. In homogeneous arrays, hawkmoths emptied more flowers with CHawkmoth shapes, but performance did not differ when shapes were standardized by surface area. However, hawkmoths visited fewer CHummingbird flowers than other shapes when surface area was standardized. 4. Our study shows that hawkmoth behaviour can be affected by differences in corolla shape, but that floral size may be more important than true shape differences in floral discrimination by hawkmoths.

Kessler, A. and R. Halitschke (2009). "Testing the potential for conflicting selection on floral chemical traits by pollinators and herbivores: predictions and case study." Functional Ecology **23**: 901-912.

1. There are myriad ways in which pollinators and herbivores can interact via the evolutionary and behavioural responses of their host plants. 2. Given that both herbivores and pollinators consume and are dependent upon plant-derived nutrients and secondary metabolites, and utilize plant signals, plant chemistry should be one of the major factors mediating these interactions. 3. Here we build upon a conceptual framework for understanding plant-mediated interactions of pollinators and herbivores. We focus on plant chemistry, in particular plant volatiles and aim to unify hypotheses for plant defence and pollination. We make predictions for the evolutionary outcomes of these interactions by hypothesizing that conflicting selection pressures from herbivores and pollinators arise from the constraints imposed by plant chemistry. 4. We further hypothesize that plants could avoid conflicts between pollinator attraction and herbivore defence through tissue-specific regulation of pollinator reward chemistry, as well as herbivore-induced changes in flower chemistry and morphology. 5. Finally, we test aspects of our predictions in a case study using a wild tomato species, Solanum peruvianum, to illustrate the diversity of tissue-specific and herbivore-induced differences in plant chemistry that could influence herbivore and pollinator behaviour, and plant fitness.

Kessler, D. and I. T. Baldwin (2007). "Making sense of nectar scents: the effects of nectar secondary metabolites on floral visitors of Nicotiana attenuata." Plant Journal **49**(5): 840-854.

Flowers produce a plethora of secondary metabolites but only nectar sugars, floral pigments and headspace volatiles have been examined in the context of pollinator behavior. We identify secondary metabolites in the headspace and nectar of glasshouse- and field-grown Nicotiana attenuata plants, infer within-flower origins by analyzing six flower parts, and compare the attractiveness of 16 constituents in standardized choice tests with two guilds of natural pollinators (Manducasexta moths and Archilochus alexandri and Selasphorus rufus hummingbirds) and one nectar thief (Solenopsis xyloni ants) to determine whether nectar metabolites can 'filter' flower visitors: only two could. Moths responded more strongly than did hummingbirds to headspace presentation of nicotine and benzylacetone, the most abundant repellent and attractant compounds, respectively. For both pollinators, nectar repellents decreased nectaring time and nectar volume removed, but increased visitation number, particularly for hummingbirds. Fewer ants visited if the nectar contained repellents. To determine whether nicotine reduced nectar removal rates in nature, we planted transformed, nicotine-silenced plants into native populations in Utah over 2 years. Plants completely lacking nicotine in their nectar had 68-70% more nectar removed per night by the native community of floral visitors than did wild-type plants. We hypothesize that nectar repellents optimize the number of flower visitors per volume of nectar produced, allowing plants to keep their nectar volumes small.

Kessler, D., et al. (2019). "The defensive function of a pollinator‐attracting floral volatile." Functional Ecology **33**(7): 1223-1232.

1. Benzyl acetone (4‐phenylbutan‐2‐one; BA), the dominant floral fragrance of the wild tobacco Nicotiana attenuata, is known to attract night‐time pollinators, such as Manduca sexta hawkmoths. For this pollinator, BA is not only essential for the pollen transport between conspecific plants, but also for the moth’s short‐distance handling of flowers at night. The emission of BA from the corolla limb, however, starts up to 4 hr before dusk, peaks at dusk and continues throughout the first half of the night. While the function of the nocturnal part of the emission is well studied, the function of the early part of the peak emission remained unknown.2. By using plants silenced in the expression of the biosynthetic gene of BA (Nachal1), we investigated the function of this early peak emission of BA in the plants´ native habitat in Southwestern Utah.3. By emitting BA before dusk, N. attenuata is able to prevent the establishment and resulting floral damage by a florivore, the cucumber beetle (Diabrotica undecimpunctata). Plants lacking BA emissions (CHAL) were not only more often colonized by florivores, but also suffered significantly more damage, than control empty vector plants (EV). Choice assays revealed that D. undecimpunctata feeding required the absence of BA. While feeding damage to CHAL flowers was higher than to EV flowers in the early night hours, the effect disappeared during the second half of the night, when flowers stop emitting BA. The close proximity of BA‐emitting flowers to non‐emitting CHAL flowers prevented D. undecimpunctata feeding. In the field, the emission of BA at dusk was sufficient to protect plants for the entire night, as beetles search for new hosts during the early evening hours and remain for the duration of the night, once a host is chosen.4. Floral BA emission before dusk may have evolved as a consequence of antagonistic interactions with florivores. A single floral volatile can thus simultaneously function as an important floral attractant for pollinators and as effective feeding deterrent against florivores in the same plant species.

Kessler, D., et al. (2013). "Petunia flowers solve the defence/apparency dilemma of pollinator attraction by deploying complex floral blends." Ecology Letters **16**(3): 299–306.

Flowers recruit floral visitors for pollination services by emitting fragrances. These scent signals can be intercepted by antagonists such as florivores to locate host plants. Hence, as a consequence of interactions with both mutualists and antagonists, floral bouquets likely consist of both attractive and defensive components. While the attractive functions of floral bouquets have been studied, their defensive function has not, and field-based evidence for the deterrence of floral-scent constituents is lacking. In field and glasshouse experiments with five lines of transgenic Petunia x hybrida plants specifically silenced in their ability to release particular components of their floral volatile bouquet, we demonstrate that the emission of single floral-scent compounds can dramatically decrease damage from generalist florivores. While some compounds are used in host location, others prevent florivory. We conclude that the complex blends that comprise floral scents are likely sculpted by the selective pressures of both pollinators and herbivores.

Kessler, D., et al. (2008). "Field experiments with transformed plants reveal the sense of floral scents." Science **321**(5893): 1200-1202.

Plants use many means to attract pollinators, including visual cues and odor. We investigated how nonpigment floral chemistry influences nectar removal, floral visitation, florivory, rates of outcrossing, and fitness through both male and female functions. We blocked expression of biosynthetic genes of the dominant floral attractant [benzyl acetone (Nachal1)] and nectar repellent [nicotine (Napmt1/2)] in all combinations in the native tobacco Nicotiana attenuata and measured their effects on plants in their native habitat. Both repellent and attractant were required to maximize capsule production and seed siring in emasculated flowers and flower visitation by native pollinators, whereas nicotine reduced florivory and nectar robbing.

Klein, A. M., et al. (2007). "Importance of pollinators in changing landscapes for world crops." Proceedings of the Royal Society B: Biological Sciences **274**(1608): 303-313.

The extent of our reliance on animal pollination for world crop production for human food has not previously been evaluated and the previous estimates for countries or continents have seldom used primary data. In this review, we expand the previous estimates using novel primary data from 200 countries and found that fruit, vegetable or seed production from 87 of the leading global food crops is dependent upon animal pollination, while 28 crops do not rely upon animal pollination. However, global production volumes give a contrasting perspective, since 60% of global production comes from crops that do not depend on animal pollination, 35% from crops that depend on pollinators, and 5% are unevaluated. Using all crops traded on the world market and setting aside crops that are solely passively self-pollinated, wind-pollinated or parthenocarpic, we then evaluated the level of dependence on animal-mediated pollination for crops that are directly consumed by humans. We found that pollinators are essential for 13 crops, production is highly pollinator dependent for 30, moderately for 27, slightly for 21, unimportant for 7, and is of unknown significance for the remaining 9. We further evaluated whether local and landscape-wide management for natural pollination services could help to sustain crop diversity and production. Case studies for nine crops on four continents revealed that agricultural intensification jeopardizes wild bee communities and their stabilizing effect on pollination services at the landscape scale.

Knauer, A. C., et al. (2018). "Crab spiders impact floral-signal evolution indirectly through removal of florivores." Nature Communications **9**(1): 1367.

The puzzling diversity of flowers is primarily shaped by selection and evolutionary change caused by the plant's interaction with animals. The contribution of individual animal species to net selection, however, may vary depending on the network of interacting organisms. Here we document that in the buckler mustard, Biscutella laevigata, the crab spider Thomisus onustus reduces bee visits to flowers but also benefits plants by feeding on florivores. Uninfested plants experience a trade-off between pollinator and spider attraction as both bees and crab spiders are attracted by the floral volatile beta-ocimene. This trade-off is reduced by the induced emission of beta-ocimene after florivore infestation, which is stronger in plant populations where crab spiders are present than where they are absent, suggesting that plants are locally adapted to the presence of crab spiders. Our study demonstrates the context-dependence of selection and shows how crab spiders impact on floral evolution.

Knudsen, J. T., et al. (2006). "Diversity and Distribution of Floral Scent." The Botanical Review **72**(1): 1-120.

A list of 1719 chemical compounds identified from headspace samples of floral scent is presented. The list has been compiled from some 270 published papers, including analyses of 991 species of flowering plants and a few gymnosperms, a sample including seed plants from 90 families and 38 orders. The compounds belong to seven major compound classes, of which the aliphatics, the benzenoids and phenylpropanoids, and, among the terpenes, the mono- and sesquiterpenes, occur in most orders of seeds plants. C5-branched compounds, irregular terpenes, nitrogen-containing compounds, and a class of miscellaneous cyclic compounds have been recorded in about two-thirds of the orders. Sulfur-containing compounds occur in a third of the orders, whereas diterpenes have been reported from three orders only. The most common single compounds in floral scent are the monoterpenes limonene, (E)-1-ocimene, myrcene, linalool, oc-and 3-pinene, and the benzenoids benzaldehyde, methyl 2-hydroxybenzoate (methyl salicylate), benzyl alcohol, and 2-phenyl ethanol, which occur in 54-71% of the families investigated so far. The sesquiterpene caryophyllene and the irregular terpene 6-methyl-5-hepten-2-one are also common and occur in more than 50% of the families. Orchidaceae are by far the best investigated family, followed by several families known to have many species with strongly scented flowers, such as Araceae, Arecaceae, Magnoliaceae, and Rosaceae. However, the majority of angiosperm families are still poorly investigated. Relationships between floral scent and pollination, chemistry, evolution, and phylogeny are briefly discussed. It is concluded that floral scent chemistry is of little use for phylogenetic estimates above the genus level, whereas the distribution and combinations of floral scent compounds at species and subspecific levels is a promising field of investigation for the understanding of adaptations and evolutionary processes in angiosperms.

Körner, C. and D. Basler (2010). "Phenology Under Global Warming." Science **327**: 1461-1462.

In most temperate tree species, phenological events such as fl owering and autumnal cessation of growth are not primarily controlled by temperature.

Koski, M. H. (2020). "The role of sensory drive in floral evolution." New Phytologist **227**(4).

Sensory drive theory posits that the evolution of communication signals is shaped by the sensory systems of receivers and the habitat conditions under which signals are received. It has inspired an enormous body of research, advancing our understanding of signal evolution and speciation in animals. In plants, the extreme diversification of floral signals has fascinated biologists for over a century. While processes involved in sensory drive probably play out in plant-pollinator communication, the theory has not been formally synthesized in this context. However, it has untapped potential to explain mechanisms underlying variation in pollinator preferences across populations, and how environmental conditions impact floral signal transmission and perception. Here I develop a framework of sensory drive for plant-pollinator interactions, identifying similarities and differences from its original conception. I then summarize studies that shed light on how the primary processes of sensory drive - habitat transmission, perceptual tuning, and signal matching - apply to the evolution of floral color and scent. Throughout, I propose research avenues and approaches to assess how sensory drive shapes floral diversity. This framework will be important for explaining patterns of extant floral diversity and examining how altered signaling conditions under global change will impact the evolutionary trajectory of floral traits.

Kudo, G. and T. Y. Ida (2013). "Early onset of spring increases the phenological mismatch between plants and pollinators." Ecology **94**(10): 2311–2320.

Climate warming accelerates the timing of flowering and insect pollinator emergence, especially in spring. If these phenological shifts progress independently between species, features of plant–pollinator mutualisms may be modified. However, evidence of phenological mismatch in pollination systems is limited. We investigated the phenologies of a spring ephemeral, Corydalis ambigua, and its pollinators (bumble bees), and seed-set success over 10–14 years in three populations. Although both flowering onset and first detection of overwintered queen bees in the C. ambigua populations were closely related to snowmelt time and/or spring temperature, flowering tended to be ahead of first pollinator detection when spring came early, resulting in lower seed production owing to low pollination service. Relationships between flowering onset time, phenological mismatch, and seed-set success strongly suggest that phenological mismatch is a major limiting factor for reproduction of spring ephemerals. This report demonstrates the mechanism of phenological mismatch and its ecological impact on plant–pollinator interactions based on long-term monitoring. Frequent occurrence of mismatch can decrease seed production and may affect the population dynamics of spring ephemerals.

Kudoh, H., et al. (2002). "Intrinsic cost of delayed flowering in annual plants: negative correlation between flowering time and reproductive effort." Plant Species Biology **17**(7): 101–107.

It has been considered that optimal flowering time evolves in annual plants because there are costs of delayed flowering that counteract the benefit of size increment by longer vegetative growth. The cost of delayed flowering in annual plants has been studied mostly in relation to external conditions, such as probability of survival. Several recent studies suggest that there are negative correlations between flowering time (FT) and reproductive effort (RE) in some annual plant species. In the present paper, we point out that a negative FT–RE relationship can be an intrinsic cost of delayed flowering in annual plants. The variation in optimal FT based on a negative FT–RE relationship was analyzed graphically and predicted patterns were compared with published empirical studies. Possible effects of negative FT–RE relationships on the ecology and evolution of FT in annual plants are discussed.

Kuppler, J. and M. M. Kotowska (2021). "A meta-analysis of responses in floral traits and flower-visitor interactions to water deficit." Glob Chang Biol **27**(13): 3095-3108.

Alterations in water availability and drought events as predicted by climate change scenarios will increasingly impact natural communities with effects already emerging at present. Water deficit leads to increasing physiological stress in plants, likely affecting floral development and causing changes in floral morphology, nectar and pollen production or scent. Understanding how these floral traits are altered by water deficit is necessary to predict changes in plant-pollinator interactions and how communities are impacted in the future. Here we employ a meta-analysis approach to synthesize the current evidence of experimental water deficit on floral traits and plant-pollinator interactions. Furthermore, we explore experimental factors potentially increasing heterogeneity between studies and provide ideas how to enhance comparability between studies. In the end, we highlight future directions and knowledge gaps for floral traits and plant-pollinator interactions under water deficit. Our analysis showed consistent decreases in floral size, number of flowers and nectar volume to reduced water availability. Other floral traits such as the start of flowering or herkogamy showed no consistent pattern. This indicates that effects of reduced water availability differ between specific traits that are potentially involved in different functions such as pollinator attraction or efficiency. We found no general decreasing visitation rates with water deficit for flower-visitor interactions. Furthermore, the comparison of available studies suggests that increased reporting of plant stress severity and including more hydraulic and physiological measurements will improve the comparability across experiments and aid a more mechanistic understanding of plant-pollinator interactions under altered environmental conditions. Overall, our results show that water deficit has the potential to strongly affect plant-pollinator interactions via changes in specific floral traits. Linking these changes to pollination services and pollinator performance is one crucial step for understanding how changing water availability and drought events under climate change will alter plant and pollinator communities.

Liu, C. Q., et al. (2019). "Floral adaptations of two lilies: implications for the evolution and pollination ecology of huge trumpet-shaped flowers." Am J Bot **106**(5): 622-632.

PREMISE: Evolutionary transitions among floral morphologies, many of which provide evidence for adaptation to novel pollinators, are common. Some trumpet-shaped flowers are among the largest flowers in angiosperms, occurring in different lineages. Our goal was to investigate the role of pollinators in the evolution of these flowers using Lilium. METHODS: We investigated floral traits and pollinators of L. primulinum var. ochraceum and L. brownii var. viridulum and reviewed reports of visitors to huge trumpet-shaped flowers. Using a published phylogeny of Lilium, we reconstructed ancestral floral morphological states in Lilium to elucidate the origins of trumpet-shaped lilies. RESULTS: Both lilies are largely self-incompatible and show floral syndromes indicative of hawkmoth pollination. The short trumpet-shaped lily can be pollinated by short-tongued (<40 mm) but not long-tongued hawkmoths (>65 mm), while the huge trumpet-shaped lily can be pollinated by both. A literature review including 22 species of trumpet-shaped flowers suggests that their pollinator guilds commonly include both short- and long-tongued moths. A phylogenetic reconstruction indicates that trumpet-shaped lilies possibly have multiple origins from tepal-reflexed ancestors, at least six of which have evolved huge flowers (>50 mm). CONCLUSIONS: Adaptation to short-tongued hawkmoths may have initiated the evolution of trumpet-shaped lilies. Huge trumpet-shaped lilies may have evolved as a response to selection by long-tongued hawkmoths, without excluding the short-tongued ones. This evolutionary pathway leads to a functionally more generalized pollination system instead of an increasingly specialized one and is not necessarily associated with pollinator shifts.

Lucas-Barbosa, D. (2016). "Integrating Studies on Plant-Pollinator and Plant-Herbivore Interactions." Trends in Plant Science **21**(2): 125-133.

Research on herbivore-induced plant defence and research on pollination ecology have had a long history of separation. Plant reproduction of most angiosperm species is mediated by pollinators, and the effects of herbivore-induced plant defences on pollinator behaviour have been largely neglected. Moreover, there is expected to be a trade-off between plant reproductive strategies and defence mechanisms. To investigate this trade-off, it is essential to study herbivore-induced plant resistance and allocation of resources by plants, within the same system, and to test if indirect plant resistance can conflict with pollinator attraction. Here, I review the key literature highlighting connection between plant defence and reproduction, and propose to exploit natural variation among plant species to assess the ecological costs of plant responses to herbivores and pollinators.

Lucas-Barbosa, D., et al. (2014). "Caught between parasitoids and predators - survival of a specialist herbivore on leaves and flowers of mustard plants." Journal of Chemical Ecology **40**(6): 621-631.

The survival of insect herbivores typically is constrained by food choice and predation risk. Here, we explored whether movement from leaves to flowers increases survival of herbivores that prefer to feed on floral tissues. Combining field and greenhouse experiments, we investigated whether flowering influences the behavior of Pieris brassicae butterflies and caterpillars and, consequently, herbivore survival in the field. In this context, we investigated also if flowers of Brassica nigra can provide caterpillars refuge from the specialist parasitoid Cotesia glomerata and from predatory social wasps. By moving to flowers, caterpillars escaped from the parasitoid. Flowers are nutritionally superior when compared with leaves, and caterpillars develop faster when feeding on flowers. However, late-stage caterpillars can be preyed upon intensively by social wasps, irrespective of whether they feed on leaves or flowers. We conclude that flower preference by P. brassicae is more likely driven by nutritional advantages and reduced parasitism on flowers, than by risks of being killed by generalist predators.

Lucas‐Barbosa, D., et al. (2015). "Visual and odour cues: plant responses to pollination and herbivory affect the behaviour of flower visitors." Functional Ecology **30**(3): 431-441.

1. Plants evolved strategies to attract pollinators that are essential for reproduction. However, plant defence against herbivores may trade off with pollinator attraction. 2. Here, we investigated the role of inducible plant secondary metabolites in such a trade-off. Our objective was to reveal the mechanisms underlying the effects of induced plant responses to pollination and herbivory. We assessed how responses of plants to pollination and insect herbivory affect the behaviour of flower visitors. Subsequently, we investigated how the production of volatile and non-volatile compounds changes after pollination and herbivory. 3. Both herbivores and pollinators induced important phenotypic changes in flowers. Brassica nigra plants respond to pollination and herbivory with changes in the profile of volatiles and non-volatiles of their flowers. Our results show that butterflies use different cues when searching for an oviposition site or a nectar source. Pollination status influenced the behaviour of butterflies, but not that of syrphid flies. 4. We discuss the results in the context of the trade-off between defence and reproduction in plants and suggest that systemic responses to herbivores can interfere with local responses to pollination. Therefore, these responses must be addressed in an integrated way because, in nature, plants are simultaneously exposed to herbivores and pollinators.

Martinez-Bauer, A. E., et al. (2015). "Multitasking in a plant-ant interaction: how does Acacia myrtifolia manage both ants and pollinators?" Oecologia **178**(2): 461-471.

Plant associations with protective ants are widespread among angiosperms, but carry the risk that ants will deter pollinators as well as herbivores. Such conflict, and adaptations to ameliorate or prevent the conflict, have been documented in African and neotropical acacias. Ant-acacia associations occur in Australia, but little is known of their ecology. Moreover, recent phylogenetic evidence indicates that Australian acacias are only distantly related to African and American acacias, providing an intercontinental natural experiment in the management of ant-pollinator conflict. We examined four populations of Acacia myrtifolia over a 400-km environmental gradient in southeastern Australia using ant and pollinator exclusion as well as direct observation of ants and pollinators to assess the potential for ant-pollinator conflict to affect seed set. Native bees were the only group of floral visitors whose visitation rates were a significant predictor of fruiting success, although beetles and wasps may play an important role as "insurance" pollinators. We found no increase in pollinator visitation or fruiting success following ant exclusion, even with large sample sizes and effective exclusion. Because ants are facultative visitors to A. myrtifolia plants, their presence may be insufficient to interfere greatly with floral visitors. It is also likely that the morphological location of extrafloral nectaries tends to draw ants away from reproductive parts, although we commonly observed ants on inflorescences, so the spatial separation is not strict. A. myrtifolia appears to maintain a generalized mutualism over a wide geographic range without the need for elaborate adaptations to resolve ant-pollinator conflict.

McCall, A. C. and R. E. Irwin (2006). "Florivory: the intersection of pollination and herbivory." Ecology Letters **9**(12): 1351-1365.

Plants interact with many visitors who consume a variety of plant tissues. While the consequences of herbivory to leaves and shoots are well known, the implications of florivory, the consumption of flowers prior to seed coat formation, have received less attention. Herbivory and florivory can yield different plant, population and community outcomes; thus, it is critical to distinguish between these two types of consumption. Here, we consider the ecological and evolutionary consequences of florivory. A growing number of studies recognize that florivory is common in natural systems and in some cases surpasses leaf herbivory in magnitude and impact. Florivores can affect male and female plant fitness via direct trophic effects and through altered pathways of species interactions. In particular, florivory can affect pollination and have consequences for plant mating and floral sexual system evolution. Plants are not defenceless against florivore damage. Concepts of resistance and tolerance can be applied to plant-florivore interactions. Moreover, extant theories of plant chemical defence, including optimal defence theory, growth rate hypothesis and growth differentiation-balance hypothesis, can be used to make testable predictions about when and how plants should defend flowers against florivores. The majority of the predictions remain untested, but they provide a theoretical foundation on which to base future experiments. The approaches to studying florivory that we outline may yield novel insights into floral and defence traits not illuminated by studies of pollination or herbivory alone.

McCall, A. C., et al. (2013). "Florivores prefer white versus pink petal color morphs in wild radish, Raphanus sativus." Oecologia **172**(1): 189-195.

Many hypotheses suggest that pollinators act to maintain or change floral color morph frequencies in nature, although pollinator preferences do not always match color morph frequencies in the field. Therefore, non-pollinating agents may also be responsible for color morph frequencies. To test this hypothesis, we examined whether Raphanus sativus plants with white flowers received different amounts of florivory than plants with pink flowers, and whether florivores preferred one floral color over the other. We found that white-flowered plants received significantly more floral damage than pink-flowered plants in eight populations over 4 years in northern California. Both generalists and specialists on Brassicaceae preferred white petals in choice and short-term no choice tests. In performance tests, generalists gained more weight on white versus pink petals whereas specialists gained similar amounts of weight on pink and white morphs. Because our results suggest that florivores prefer and perform better on white versus pink flowers, these insects may have the opportunity to affect the frequency of color morphs in the field.

Moreira, X., et al. (2019). "A meta-analysis of herbivore effects on plant attractiveness to pollinators." Ecology **100**(6): e02707.

Herbivores may directly or indirectly affect plant attractiveness to pollinators. Although several studies have reported on these effects, there is yet no general consensus on the strength and sign of such interactions or their contingency on herbivory features such as the plant tissue attacked. We performed a meta-analysis of studies testing for effects of herbivores on floral traits, plant attractiveness to pollinators, and plant reproductive success. We also assessed whether herbivore effects depended on the plant tissue attacked by herbivores and if real or simulated herbivory was used. We found an overall significant negative effect of herbivores on floral traits, plant attractiveness to pollinators, and plant reproductive success. These effects were, however, contingent on the plant tissue attacked and on whether real or simulated damage was used. Real floral and leaf, but not root, herbivores showed detrimental effects on floral traits and plant attractiveness to pollinators. In addition, real leaf, but not floral or root herbivory, lowered plant reproductive success. Contrastingly, simulated leaf and floral herbivory showed no effect on any of the response variables. These findings help move forward our understanding of the strength and directionality of herbivore effects on plant attractiveness to pollinators and their underlying mechanisms.

Mosleh Arany, A., et al. (2008). "Herbivory and local genetic differentiation in natural populations of Arabidopsis thaliana (Brassicaceae)." Plant Ecology **201**(2): 651-659.

To explore genetic variation in defence against the natural herbivores of Arabidopsis thaliana, we transplanted genotypes between a dune habitat and inland habitat in both of which A. thaliana occurred naturally. In previous years we had observed that the specialist weevils Ceutorhynchus atomus and C. contractus (Curculionidae) fed conspicuously on flowers and fruits of A. thaliana in the dunes, while these weevils were always rare in inland habitats. Taking all plants together, total fruit damage was indeed much higher in our experimental plots in the dune habitat (59.7%) relative to the inland garden habitat (18.9%). Within a habitat, additional differences existed between plants of different origins, pointing to genetic differences in ecologically relevant characters; plants of inland origin flowered a week earlier, grew better and produced more fruits than plants of dune origin. However, plants of inland origin experienced more total fruit damage by the specialist weevils (75.4%) than plants of dune origin (44.0%) when the two types grew side by side in the dune habitat. Escape from herbivory gives dune genotypes an advantage in their native habitat, whereas stronger growth and higher survival gives inland genotypes an edge under garden conditions.

Moyroud, E. and B. J. Glover (2017). "The Evolution of Diverse Floral Morphologies." Current Biology **27**(17): R941-R951.

The angiosperm flower develops through a modular programme which, although ancient and conserved, provides the flexibility that has allowed an almost infinite variety of floral forms to emerge. In this review, we explore the evolution of floral diversity, focusing on our recent understanding of the mechanistic basis of evolutionary change. We discuss the various ways in which flower size and floral organ size can be modified, the means by which flower shape and symmetry can change, and the ways in which floral organ position can be varied. We conclude that many challenges remain before we fully understand the ecological and molecular processes that facilitate the diversification of flower structure.

Muhlemann, J. K., et al. (2014). "Floral volatiles: from biosynthesis to function." Plant, Cell & Environment **37**(8): 1936-1949.

Floral volatiles have attracted humans' attention since antiquity and have since then permeated many aspects of our lives. Indeed, they are heavily used in perfumes, cosmetics, flavourings and medicinal applications. However, their primary function is to mediate ecological interactions between flowers and a diverse array of visitors, including pollinators, florivores and pathogens. As such, they ultimately ensure the plants' reproductive and evolutionary success. To date, over 1700 floral volatile organic compounds (VOCs) have been identified. Interestingly, they are derived from only a few biochemical networks, which include the terpenoid, phenylpropanoid/benzenoid and fatty acid biosynthetic pathways. These pathways are intricately regulated by endogenous and external factors to enable spatially and temporally controlled emission of floral volatiles, thereby fine-tuning the ecological interactions facilitated by floral volatiles. In this review, we will focus on describing the biosynthetic pathways leading to floral VOCs, the regulation of floral volatile emission, as well as biological functions of emitted volatiles.

Muhlemann, J. K., et al. (2006). "Postpollination Changes in Floral Odor in Silene latifolia: Adaptive Mechanisms for Seed-Predator Avoidance?" Journal of Chemical Ecology **32**: 1855–1860.

Floral odor is a key trait for pollinator attraction in many plants, but may also direct antagonists like herbivores to flowers. In this study, we examined how floral scent changes after pollination in Silene latifolia, which has a specialized relationship with the seed predator Hadena bicruris. We found an overall decrease in total scent emission and considerable changes in relative amounts of scent compounds after pollination. Lilac aldehydes A and B as well as veratrole contributed most to the decrease in scent emission. These three compounds are known to be key signals for the attraction of H. bicruris to the flowers. A specific downregulation of these compounds may increase the reproductive success of the plant by reducing seed predation after pollination.

Munguia-Rosas, M. A., et al. (2011). "Meta-analysis of phenotypic selection on flowering phenology suggests that early flowering plants are favoured." Ecology Letters **14**(5): 511-521.

Flowering times of plants are important life-history components and it has previously been hypothesized that flowering phenologies may be currently subject to natural selection or be selectively neutral. In this study we reviewed the evidence for phenotypic selection acting on flowering phenology using ordinary and phylogenetic meta-analysis. Phenotypic selection exists when a phenotypic trait co-varies with fitness; therefore, we looked for studies reporting an association between two components of flowering phenology (flowering time or flowering synchrony) with fitness. Data sets comprising 87 and 18 plant species were then used to assess the incidence and strength of phenotypic selection on flowering time and flowering synchrony, respectively. The influence of dependence on pollinators, the duration of the reproductive event, latitude and plant longevity as moderators of selection were also explored. Our results suggest that selection favours early flowering plants, but the strength of selection is influenced by latitude, with selection being stronger in temperate environments. However, there is no consistent pattern of selection on flowering synchrony. Our study demonstrates that phenotypic selection on flowering time is consistent and relatively strong, in contrast to previous hypotheses of selective neutrality, and has implications for the evolution of temperate floras under global climate change.

Narbona, E., et al. (2018). "Flower colour polymorphism in the Mediterranean Basin: occurrence, maintenance and implications for speciation." Plant Biol (Stuttg) **20 Suppl 1**: 8-20.

Flower colour polymorphism (FCP) is the occurrence of at least two discrete flower colour variants in the same population. Despite a vast body of research concerning the maintenance and evolutionary consequences of FCP, only recently has the spatial variation in morph frequencies among populations been explored. Here we summarise the biochemical and genetic basis of FCP, the factors that have been proposed to explain their maintenance, and the importance of FCP and its geographic variation in the speciation process. We also review the incidence of FCP in the environmentally heterogeneous Mediterranean Basin. Nearly 88% of Mediterranean FCP species showed anthocyanin-based polymorphisms. Concerning the evolutionary mechanisms that contribute to maintain FCP, selection by pollinators is suggested in some species, but in others, selection by non-pollinator agents, genetic drift or gene flow are also found; in some cases different processes interact in the maintenance of FCP. We emphasise the role of both autonomous selfing and clonal reproduction in FCP maintenance. Mediterranean polymorphic species show mainly monomorphic populations with only a few polymorphic ones, which generate clinal or mosaic patterns of variation in FCP. No cases of species with only polymorphic populations were found. We posit that different evolutionary processes maintaining polymorphism the Mediterranean Basin will result in a continuum of geographic patterns in morph compositions and relative frequencies of FCP species.

Niet, T. v. d. (2021). "Paucity of natural history data impedes phylogenetic analyses of pollinator-driven evolution." New Phytologist **229**(3): 1201-1205.

Ollerton, J. (2017). "Pollinator Diversity: Distribution, Ecological Function, and Conservation." Annual Review of Ecology, Evolution, and Systematics **48**(1): 353-376.

By facilitating plant reproduction, pollinators perform a crucial ecological function that supports the majority of the world’s plant diversity, and associated organisms, and a significant fraction of global agriculture. Thus, pollinators are simultaneously vital to supporting both natural ecosystems and human food security, which is a unique position for such a diverse group oforganisms. The past two decades have seen unprecedented interest in pollinators and pollination ecology, stimulated in part by concerns about the decline of pollinator abundance and diversity in some parts of the world. This review synthesizes what is currently understood about the taxonomic diversity oforganisms that are known to act as pollinators; their distribution in both deep time and present space; the importance of their diversity for ecological function (including agro-ecology); changes to diversity and abundance over more recent timescales, including introduction of non-native species; and a discussion of arguments for conserving their diversity.

Ollerton, J., et al. (2009). "A global test of the pollination syndrome hypothesis." Ann Bot **103**(9): 1471-1480.

BACKGROUND AND AIMS: 'Pollination syndromes' are suites of phenotypic traits hypothesized to reflect convergent adaptations of flowers for pollination by specific types of animals. They were first developed in the 1870s and honed during the mid 20th Century. In spite of this long history and their central role in organizing research on plant-pollinator interactions, the pollination syndromes have rarely been subjected to test. The syndromes were tested here by asking whether they successfully capture patterns of covariance of floral traits and predict the most common pollinators of flowers. METHODS: Flowers in six communities from three continents were scored for expression of floral traits used in published descriptions of the pollination syndromes, and simultaneously the pollinators of as many species as possible were characterized. KEY RESULTS: Ordination of flowers in a multivariate 'phenotype space' defined by the syndromes showed that almost no plant species fall within the discrete syndrome clusters. Furthermore, in approximately two-thirds of plant species, the most common pollinator could not be successfully predicted by assuming that each plant species belongs to the syndrome closest to it in phenotype space. CONCLUSIONS: The pollination syndrome hypothesis as usually articulated does not successfully describe the diversity of floral phenotypes or predict the pollinators of most plant species. Caution is suggested when using pollination syndromes for organizing floral diversity, or for inferring agents of floral adaptation. A fresh look at how traits of flowers and pollinators relate to visitation and pollen transfer is recommended, in order to determine whether axes can be identified that describe floral functional diversity more successfully than the traditional syndromes.

Ollerton, J., et al. (2011). "How many flowering plants are pollinated by animals?" OIKOS **120**(3): 321-326.

It is clear that the majority of fl owering plants are pollinated by insects and other animals, with a minority utilising abiotic pollen vectors, mainly wind. However there is no accurate published calculation of the proportion of the ca 352 000 species of angiosperms that interact with pollinators. Widely cited fi gures range from 67% to 96% but these have not been based on fi rm data. We estimated the number and proportion of fl owering plants that are pollinated by animals using published and unpublished community-level surveys of plant pollination systems that recorded whether each species present was pollinated by animals or wind. Th e proportion of animal-pollinated species rises from a mean of 78% in temperate-zone communities to 94% in tropical communities. By correcting for the latitudinal diversity trend in fl owering plants, we estimate the global number and proportion of animal pollinated angiosperms as 308 006, which is 87.5% of the estimated species-level diversity of fl owering plants. Given current concerns about the decline in pollinators and the possible resulting impacts on both natural communities and agricultural crops, such estimates are vital to both ecologists and policy makers. Further research is required to assess in detail the absolute dependency of these plants on their pollinators, and how this varies with latitude and community type, but there is no doubt that plant – pollinator interactions play a signifi cant role in maintaining the functional integrity of most terrestrial ecosystems.

Pereira, R. A. S. and F. Kjellberg (2021). Mutualism as a Source of Evolutionary Innovation: Insights from Insect-Plant Interactions. Plant-Animal Interactions**:** 307-332.

Pilson, D. (2000). "Herbivory and natural selection on flowering phenology in wild sunflower, Helianthus annuus." Oecologia **122**(1): 72-82.

Plant fitness is strongly affected by flowering phenology, and there are several ecological factors that are thought to shape the distribution of flowering times. One relatively underexamined factor is the timing and intensity of attack by herbivores that feed on flowers or developing seeds. This study tests the hypothesis that herbivores that feed on developing seeds of wild sunflower, Helianthus annuus (Asteraceae), impose selection on flowering phenology. First, the study population was found to contain genetic variation for mean date of flowering, so this trait could evolve if natural selection were operating. Next, the phenological pattern of abundance of five seed-feeding herbivores was documented. Damage by three herbivores, Haplorhynchites aeneus (Cucurlionidae), the head-clipping weevil, Homoeosoma electellum (Lepidoptera: Pyralidae), the sunflower moth, and Suleima helianthana (Lepidoptera: Tortricidae), the sunflower bud moth, was highest early in the flowering season, and declined as the season progressed. Damage by one herbivore, the seed fly Gymnocarena diffusa (Diptera: Tephrididae), was lowest early in the flowering season and increased as the season progressed. Finally, damage by two seed weevils, Smicronyx fulvus and S. sordidus (Curculionidae), whose damage was not distinguished, was constant through the flowering period. Third, damage by Haplorhynchites, Homoeosoma, and Suleima was found to be detrimental to plant fitness, suggesting that plants that flower when these herbivores are not abundant should have higher fitness. Finally, two phenotypic selection analyses were performed. The first included damage by Homoeosoma and Suleima, as well as flowering date, leaf area, and inflorescence diameter, as characters predicting plant fitness. In this analysis directional selection was found to act to decrease damage by the two herbivores, but did not act on flowering date. The second selection analysis was identical except that damage by the two herbivores was not included. In this analysis significant directional selection was found to favor later-flowering plants. Comparison of these two analyses suggests that all selection on flowering phenology is attributable to damage by Homoeosoma and Suleima: plants that flower later avoid damage by these two herbivores. While other influences on flowering phenology, such as pollination, mate availability, and seasonality, have been well documented, this study is one of few to demonstrate natural selection on flowering phenology that is a direct consequence of insect attack.

Primack, R. B. (1985). "Longevity of Individual Flowers." Annual Review of Ecology & Systematics **16**(16): 15-37.

Proffit, M., et al. (2020). "Chemical signal is in the blend: bases of plant-pollinator encounter in a highly specialized interaction." Sci Rep **10**(1): 10071.

In several highly specialized plant-insect interactions, scent-mediated specificity of pollinator attraction is directed by the emission and detection of volatile organic compounds (VOCs). Although some plants engaged in such interactions emit singular compounds, others emit mixtures of VOCs commonly emitted by plants. We investigated the chemical ecological bases of host plant recognition in the nursery pollination mutualism between the dioecious Ficus carica and its specific pollinator Blastophaga psenes. Using Y-tube olfactometer tests, we show that B. psenes females are attracted by VOCs of receptive figs of both sexes and do not exhibit preference for VOCs of either male or female figs. Electrophysiological tests and chemical analysis revealed that of all the VOCs emitted by receptive figs, only five were found to be active on female antennae. Behavioural tests show that, in contrast to VOCs presented alone, only a blend with a particular proportion of four of these VOCs is as attractive as the odour of receptive figs, and that if there is a very small change in this blend proportion, the pollinator is no longer attracted. This study revealed that in highly specialized mutualistic interactions specificity could be mediated by a particular blend of common compounds emitted by plants.

Rafferty, N. E., et al. (2015). "Phenological shifts and the fate of mutualisms." OIKOS **124**(1): 14-21.

Climate change is altering the timing of life history events in a wide array of species, many of which are involved in mutualistic interactions. Because many mutualisms can form only if partner species are able to locate each other in time, differential phenological shifts are likely to influence their strength, duration and outcome. At the extreme, climate change-driven shifts in phenology may result in phenological mismatch: the partial or complete loss of temporal overlap of mutualistic species. We have a growing understanding of how, when, and why phenological change can alter one type of mutualism-pollination. However, as we show here, there has been a surprising lack of attention to other types of mutualism. We generate a set of predictions about the characteristics that may predispose mutualisms in general to phenological mismatches. We focus not on the consequences of such mismatches but rather on the likelihood that mismatches will develop. We explore the influence of three key characteristics of mutualism: 1) intimacy, 2) seasonality and duration, and 3) obligacy and specificity. We predict that the following characteristics of mutualism may increase the likelihood of phenological mismatch: 1) a non-symbiotic life history in which co-dispersal is absent; 2) brief, seasonal interactions; and 3) facultative, generalized interactions. We then review the limited available data in light of our a priori predictions and point to mutualisms that are more and less likely to be at risk of becoming phenologically mismatched, emphasizing the need for research on mutualisms other than plant-pollinator interactions. Future studies should explicitly focus on mutualism characteristics to determine whether and how changing phenologies will affect mutualistic interactions.

Raine, N. E. and L. Chittka (2007). "The adaptive significance of sensory bias in a foraging context: floral colour preferences in the bumblebee Bombus terrestris." PLoS One **2**(6): e556.

Innate sensory biases could play an important role in helping naive animals to find food. As inexperienced bees are known to have strong innate colour biases we investigated whether bumblebee (Bombus terrestris) colonies with stronger biases for the most rewarding flower colour (violet) foraged more successfully in their local flora. To test the adaptive significance of variation in innate colour bias, we compared the performance of colour-naive bees, from nine bumblebee colonies raised from local wild-caught queens, in a laboratory colour bias paradigm using violet (bee UV-blue) and blue (bee blue) artificial flowers. The foraging performance of the same colonies was assessed under field conditions. Colonies with a stronger innate bias for violet over blue flowers in the laboratory harvested more nectar per unit time under field conditions. In fact, the colony with the strongest bias for violet (over blue) brought in 41% more nectar than the colony with the least strong bias. As violet flowers in the local area produce more nectar than blue flowers (the next most rewarding flower colour), these data are consistent with the hypothesis that local variation in flower traits could drive selection for innate colour biases.

Raine, N. E., et al. (2006). Adaptation, Genetic Drift, Pleiotropy, and History in the Evolution of Bee Foraging Behavior**:** 305-354.

Rebecca E. Irwin, S. Y. S., Shonna Storz, Aimee Emerson, Genevieve Guibert (2003). "The role of herbivores in the maintenance of a flower color polymorphism in wild radish." Ecology **84**(7): 1733-1743.

Plant species exhibiting polymorphisms with respect to flower color are widespread. Our understanding of the selection pressures that may maintain these color polymorphisms has primarily been confined to one set of organisms—pollinators. Yet, selection on flower color may also be driven by other agents, such as herbivores, especially in cases where pollinators and herbivores are using the same or correlated traits to select plants. A wealth ofstudies have documented pollinator preference for anthocyanin-recessive color morphs (A⫺; yellow and white flowers) of wild radish, Raphanus sativus, over anthocyanin-dominant morphs (A⫹; pink and bronze flowers); yet, differences in pollination alone do not explain the maintenance of the flower color polymorphism. Here, we ask whether variation in flower color in R. sativus influences the preference and performance of herbivores for A⫺ flower color morphs vs. A⫹ flower color morphs in four types of herbivores (generalist and specialist Lepidoptera, slugs, aphids, and thrips). We found that all herbivores except for aphids and thrips preferred flowering A⫺ color morphs compared to A⫹ morphs of R. sativus. Furthermore, all herbivores except larvae of specialist and generalist Lepidoptera performed better on A⫺ color morphs. Differences in plant secondary chemistry may play a role in differential herbivore preference and performance on the anthocyanin flower color morphs. Chemical analyses of leaf secondary compounds (indole glucosinolates) revealed that A⫹ color morphs produced higher concentrations of indole glucosinolates than A⫺ morphs in the presence of herbivore damage. Therefore, herbivores may exhibit lower preference for A⫹ color morphs, and these morphs may support lower herbivore performance because they are heavily defended once damaged. This is the first study, to our knowledge, to document differential preference and performance ofherbivores for different flower color morphs. Previous studies have shown that increased herbivore damage can have profound negative direct and indirect effects on the reproduction of R. sativus. The data presented here suggest that differential preference and performance of herbivores for R. sativus color morphs may counter selection on flower color exerted by pollinators

Reich, D., et al. (2020). "Modularity and evolution of flower shape: the role of function, development, and spandrels in Erica." New Phytologist **226**(1): 267-280.

Flowers have been hypothesized to contain either modules of attraction and reproduction, functional modules (pollination-effecting parts) or developmental modules (organ-specific). Do pollination specialization and syndromes influence floral modularity? In order to test these hypotheses and answer this question, we focused on the genus Erica: we gathered 3D data from flowers of 19 species with diverse syndromes via computed tomography, and for the first time tested the above-mentioned hypotheses via 3D geometric morphometrics. To provide an evolutionary framework for our results, we tested the evolutionary mode of floral shape, size and integration under the syndromes regime, and - for the first time - reconstructed the high-dimensional floral shape of their most recent common ancestor. We demonstrate that the modularity of the 3D shape of generalist flowers depends on development and that of specialists is linked to function: modules of pollen deposition and receipt in bird syndrome, and access-restriction to the floral reward in long-proboscid fly syndrome. Only size and shape principal component 1 showed multiple-optima selection, suggesting that they were co-opted during evolution to adapt flowers to novel pollinators. Whole floral shape followed an Ornstein-Uhlenbeck (selection-driven) evolutionary model, and differentiated relatively late. Flower shape modularity thus crucially depends on pollinator specialization and syndrome.

Riffell, J. A., et al. (2013). "Neural basis of a pollinator's buffet: olfactory specialization and learning in Manduca sexta." Science **339**(6116): 200-204.

Pollinators exhibit a range of innate and learned behaviors that mediate interactions with flowers, but the olfactory bases of these responses in a naturalistic context remain poorly understood. The hawkmoth Manduca sexta is an important pollinator for many night-blooming flowers but can learn--through olfactory conditioning--to visit other nectar resources. Analysis of the flowers that are innately attractive to moths shows that the scents all have converged on a similar chemical profile that, in turn, is uniquely represented in the moth's antennal (olfactory) lobe. Flexibility in visitation to nonattractive flowers, however, is mediated by octopamine-associated modulation of antennal-lobe neurons during learning. Furthermore, this flexibility does not extinguish the innate preferences. Such processing of stimuli through two olfactory channels, one involving an innate bias and the other a learned association, allows the moths to exist within a dynamic floral environment while maintaining specialized associations.

Roddy, A. B., et al. (2020). "Towards the flower economics spectrum." New Phytologist.

Understanding how floral traits affect reproduction is key for understanding genetic diversity, speciation, and trait evolution in the face of global changes and pollinator decline. However, there has not yet been a unified framework to characterize the major trade-offs and axes of floral trait variation. Here, we propose the development of a floral economics spectrum (FES) that incorporates the multiple pathways by which floral traits can be shaped by multiple agents of selection acting on multiple flower functions. For example, while pollinator-mediated selection has been considered the primary factor affecting flower evolution, selection by nonpollinator agents can reinforce or oppose pollinator selection, and, therefore, affect floral trait variation. In addition to pollinators, the FES should consider nonpollinator biotic agents and floral physiological costs, broadening the drivers of floral traits beyond pollinators. We discuss how coordinated evolution and trade-offs among floral traits and between floral and vegetative traits may influence the distribution of floral traits across biomes and lineages, thereby influencing organismal evolution and community assembly.

Rodriguez-Saona, C., et al. (2011). "Variation in highbush blueberry floral volatile profiles as a function of pollination status, cultivar, time of day and flower part: implications for flower visitation by bees." Annals of Botany **107**(8): 1377-1390.

BACKGROUND AND AIMS: Studies of the effects of pollination on floral scent and bee visitation remain rare, particularly in agricultural crops. To fill this gap, the hypothesis that bee visitation to flowers decreases after pollination through reduced floral volatile emissions in highbush blueberries, Vaccinium corymbosum, was tested. Other sources of variation in floral emissions and the role of floral volatiles in bee attraction were also examined. METHODS: Pollinator visitation to blueberry flowers was manipulated by bagging all flowers within a bush (pollinator excluded) or leaving them unbagged (open pollinated), and then the effect on floral volatile emissions and future bee visitation were measured. Floral volatiles were also measured from different blueberry cultivars, times of the day and flower parts, and a study was conducted to test the attraction of bees to floral volatiles. KEY RESULTS: Open-pollinated blueberry flowers had 32 % lower volatile emissions than pollinator-excluded flowers. In particular, cinnamyl alcohol, a major component of the floral blend that is emitted exclusively from petals, was emitted in lower quantities from open-pollinated flowers. Although, no differences in cinnamyl alcohol emissions were detected among three blueberry cultivars or at different times of day, some components of the blueberry floral blend were emitted in higher amounts from certain cultivars and at mid-day. Field observations showed that more bees visited bushes with pollinator-excluded flowers. Also, more honey bees were caught in traps baited with a synthetic blueberry floral blend than in unbaited traps. CONCLUSIONS: Greater volatile emissions may help guide bees to unpollinated flowers, and thus increase plant fitness and bee energetic return when foraging in blueberries. Furthermore, the variation in volatile emissions from blueberry flowers depending on pollination status, plant cultivar and time of day suggests an adaptive role of floral signals in increasing pollination of flowers.

Roguz, K., et al. (2021). "Visibility and attractiveness of Fritillaria (Liliaceae) flowers to potential pollinators." Sci Rep **11**(1): 11006.

Visual floral characters play an important role in shaping plant-pollinator interactions. The genus Fritillaria L. (Liliaceae), comprising approximately 140 species, is described as displaying a remarkable variety of flower colours and sizes. Despite this variation in visual floral traits of fritillaries, little is known about the potential role of these features in shaping plant-pollinator interactions. Here, we seek to clarify the role of visual attraction in species offering a robust food reward for pollinators early in the spring, which is the case for Fritillaria. We also searched for potential tendencies in the evolution of floral traits crucial for plant-pollinator communication. The generality of species with green and purple flowers may indicate an influence of environmental factors other than pollinators. The flowers of the studied species seem to be visible but not very visually attractive to potential pollinators. The food rewards are hidden within the nodding perianth, and both traits are conserved among fritillaries. Additionally, visual floral traits are not good predictors of nectar properties. When in the flowers, pollinators are navigated by nectar guides in the form of contrasting nectary area colouration. Flower colour does not serve as a phenotypic filter against illegitimate pollinators-red and orange bird-pollinated fritillaries are visible to bees.

Romero, G. Q. and J. Vasconcellos-Neto (2004). "Beneficial effects of flower-dwelling predators on their host plant." Ecology **85**(2): 446–457.

We examined the effects of the sit-and-wait spider Misumenops argenteus (Thomisidae) on the herbivore assemblage and fitness of the perennial woody shrub Trichogoniopsis adenantha (Asteraceae). Because crab spiders prey on both pollinators and phytophagous insects, they can have potentially negative and positive effects on plants. In a manipulative experiment using paired plants, spiders decreased the density of sucking and some endophagous herbivores on the leaves and capitula and reduced the number of damaged achenes produced by the plants. Damaged capitula had a higher proportion of fertilized achenes in plants with spiders than without spiders, but not undamaged capitula. These results indicate that M. argenteus exerted a double positive effect on seed production in T. adenantha. The effect of M. argenteus on herbivores may be taxon specific and vary among years with different herbivore abundances.

Rosas-Guerrero, V., et al. (2014). "A quantitative review of pollination syndromes: do floral traits predict effective pollinators?" Ecol Lett **17**(3): 388-400.

The idea of pollination syndromes has been largely discussed but no formal quantitative evaluation has yet been conducted across angiosperms. We present the first systematic review of pollination syndromes that quantitatively tests whether the most effective pollinators for a species can be inferred from suites of floral traits for 417 plant species. Our results support the syndrome concept, indicating that convergent floral evolution is driven by adaptation to the most effective pollinator group. The predictability of pollination syndromes is greater in pollinator-dependent species and in plants from tropical regions. Many plant species also have secondary pollinators that generally correspond to the ancestral pollinators documented in evolutionary studies. We discuss the utility and limitations of pollination syndromes and the role of secondary pollinators to understand floral ecology and evolution.

Ruedenauer, F. A., et al. (2015). "How to know which food is good for you: bumblebees use taste to discriminate between different concentrations of food differing in nutrient content." J Exp Biol **218**(Pt 14): 2233-2240.

In view of the ongoing pollinator decline, the role of nutrition in bee health has received increasing attention. Bees obtain fat, carbohydrates and protein from pollen and nectar. As both excessive and deficient amounts of these macronutrients are detrimental, bees would benefit from assessing food quality to guarantee an optimal nutrient supply. While bees can detect sucrose and use it to assess nectar quality, it is unknown whether they can assess the macronutrient content of pollen. Previous studies have shown that bees preferentially collect pollen of higher protein content, suggesting that differences in pollen quality can be detected either by individual bees or via feedback from larvae. In this study, we examined whether and, if so, how individuals of the buff-tailed bumblebee (Bombus terrestris) discriminate between different concentrations of pollen and casein mixtures and thus nutrients. Bumblebees were trained using absolute and differential conditioning of the proboscis extension response (PER). As cues related to nutrient concentration could theoretically be perceived by either smell or taste, bees were tested on both olfactory and, for the first time, chemotactile perception. Using olfactory cues, bumblebees learned and discriminated between different pollen types and casein, but were unable to discriminate between different concentrations of these substances. However, when they touched the substances with their antennae, using chemotactile cues, they could also discriminate between different concentrations. Bumblebees are therefore able to discriminate between foods of different concentrations using contact chemosensory perception (taste). This ability may enable them to individually regulate the nutrient intake of their colonies.

Rusman, Q., et al. (2020). "Plant ontogeny determines strength and associated plant fitness consequences of plant-mediated interactions between herbivores and flower visitors." J Ecol **108**(3): 1046-1060.

Plants show ontogenetic variation in growth-defence strategies to maximize reproductive output within a community context. Most work on plant ontogenetic variation in growth-defence trade-offs has focussed on interactions with antagonistic insect herbivores. Plants respond to herbivore attack with phenotypic changes. Despite the knowledge that plant responses to herbivory affect plant mutualistic interactions with pollinators required for reproduction, indirect interactions between herbivores and pollinators have not been included in the evaluation of how ontogenetic growth-defence trajectories affect plant fitness.In a common garden experiment with the annual Brassica nigra, we investigated whether exposure to various herbivore species on different plant ontogenetic stages (vegetative, bud or flowering stage) affects plant flowering traits, interactions with flower visitors and results in fitness consequences for the plant.Effects of herbivory on flowering plant traits and interactions with flower visitors depended on plant ontogeny. Plant exposure in the vegetative stage to the caterpillar Pieris brassicae and aphid Brevicoryne brassicae led to reduced flowering time and flower production, and resulted in reduced pollinator attraction, pollen beetle colonization, total seed production and seed weight. When plants had buds, infestation by most herbivore species tested reduced flower production and pollen beetle colonization. Pollinator attraction was either increased or reduced. Plants infested in the flowering stage with P. brassicae or Lipaphis erysimi flowered longer, while infestation by any of the herbivore species tested increased the number of flower visits by pollinators.Our results show that the outcome of herbivore-flower visitor interactions in B. nigra is specific for the combination of herbivore species and plant ontogenetic stage. Consequences of herbivory for flowering traits and reproductive output were strongest when plants were attacked early in life. Such differences in selection pressures imposed by herbivores to specific plant ontogenetic stages may drive the evolution of distinct ontogenetic trajectories in growth-defence-reproduction strategies and include indirect interactions between herbivores and flower visitors. Synthesis. Plant ontogeny can define the direct and indirect consequences of herbivory. Our study shows that the ontogenetic stage of plant individuals determined the effects of herbivory on plant flowering traits, interactions with flower visitors and plant fitness.

Rusman, Q., et al. (2019). "Ecology of Plastic Flowers." Trends in Plant Science **24**(8): 725-740.

Plant phenotypic plasticity in response to herbivore attack includes changes in flower traits. Such herbivore-induced changes in flower traits have consequences for interactions with flower visitors. We synthesize here current knowledge on the specificity of herbivore-induced changes in flower traits, the underlying molecular mechanisms, and the ecological consequences for flower-associated communities. Herbivore-induced changes in flower traits seem to be largely herbivore species-specific. The extensive plasticity observed in flowers influences a highly connected web of interactions within the flowerassociated community. We argue that the adaptive value of herbivore-induced plant responses and flower plasticity can be fully understood only from a community perspective rather than from pairwise interactions.

Rusman, Q., et al. (2018). "Dealing with mutualists and antagonists: Specificity of plant‐mediated interactions between herbivores and flower visitors, and consequences for plant fitness." Functional Ecology **32**(4): 1022-1035.

1. Plants need to deal with antagonists, such as herbivores, while maintaining interactions with mutualists, such as pollinators that help plants to maximize their reproductive output. Although many plant species have inducible defences to save metabolic costs of defence in the absence of herbivores, plant responses induced by herbivore attack can have ecological costs. For example, herbivore-induced responses can affect flower traits and alter interactions with flower visitors. Such plantmediated interactions between herbivores and flower visitors can affect plant reproductive output. Current knowledge on the generality and specificity of plant-mediated herbivore–flower–visitor interactions and its consequences for plant fitness is limited. 2. In this study, we investigated whether a broad range of herbivores feeding on the annual plant Brassica nigra affect interactions with flower visitors, whether the direction of interactions is predicted by the feeding modes (chewing and sap-feeding) and sites (above- and belowground) of the herbivores, and whether it results in fitness consequences for the plant. 3. Our results show that attack of B. nigra by a range of different herbivores influenced plant interactions with mutualist pollinators and an antagonist florivore, the pollen beetle Meligethes aeneus. Pollinator community composition was affected by herbivory, whereas overall pollinator attraction was maintained. Pollinator community composition of uninfested plants differed from that of chewing and root herbivore-infested plants. Main responders in the pollinator communities to changes induced by herbivory were syrphid flies, bumblebees, and solitary bees. Although the preference of pollen beetle adults was not affected by herbivory, beetle larvae performed best on plants infested with the nematode Heterodera schachtii. The changes in pollinator community composition and syrphid fly visitation can explain the observed increase in seed set of root herbivore-infested plants. 4. Interactions of flowering B. nigra plants with mutualist and antagonist insects are well integrated and conflicting interactions do not reduce reproductive output. Our results suggest some degree of specificity in herbivore–flower–visitor interactionswith consequences for plant fitness. Specificity of plant responses were determined at the species level as well as the herbivore functional group level, and differed depending on the flower visitor. Because plant reproduction was affected by indirect plant-mediated interactions, these can potentially result in selection on plant strategies to optimize growth, defence and reproduction.

Rusman, Q., et al. (2019). "Floral plasticity: Herbivore-species-specific-induced changes in flower traits with contrasting effects on pollinator visitation." Plant, Cell & Environment **42**(6): 1882-1896.

Plant phenotypic plasticity in response to antagonists can affect other community members such as mutualists, conferring potential ecological costs associated with inducible plant defence. For flowering plants, induction of defences to deal with herbivores can lead to disruption of plant-pollinator interactions. Current knowledge on the full extent of herbivore-induced changes in flower traits is limited, and we know little about specificity of induction of flower traits and specificity of effect on flower visitors. We exposed flowering Brassica nigra plants to six insect herbivore species and recorded changes in flower traits (flower abundance, morphology, colour, volatile emission, nectar quantity, and pollen quantity and size) and the behaviour of two pollinating insects. Our results show that herbivory can affect multiple flower traits and pollinator behaviour. Most plastic floral traits were flower morphology, colour, the composition of the volatile blend, and nectar production. Herbivore-induced changes in flower traits resulted in positive, negative, or neutral effects on pollinator behaviour. Effects on flower traits and pollinator behaviour were herbivore species-specific. Flowers show extensive plasticity in response to antagonist herbivores, with contrasting effects on mutualist pollinators. Antagonists can potentially act as agents of selection on flower traits and plant reproduction via plant-mediated interactions with mutualists.

Sahli, H. F. and J. K. Conner (2011). "Testing for conflicting and nonadditive selection: floral adaptation to multiple pollinators through male and female fitness." Evolution **65**(5): 1457-1473.

Although conflicting selection from different resources is thought to play a critical role in the evolution of specialized species, the prevalence of conflicting selection in generalists is poorly understood. Plants may experience conflicting selection on floral traits by different pollinators and between genders. Using artificial selection to increase phenotypic variation, we tested for conflicting and nonadditive selection on wild radish (Raphanus raphanistrum) flowers. To do this, we measured selection by each of the major pollinator taxa through both male and female fitness, and tested for a single-generation response to selection by a subset of these pollinators. We found some evidence for conflicting selection on anther exertion--sweat bees exerted stabilizing selection and larger bees selected for increased exertion. Stamen dimorphism was only under selection by honey bees, causing a response to selection in the next generation, and flower size was under similar selection by multiple pollinators. Selection differed significantly between genders for two traits, but there was no evidence for stronger selection through male fitness or for conflicting selection between genders. Our results suggest wild radish flowers can adapt to multiple pollinators, as we found little evidence for conflicting selection and no evidence for nonadditive selection among pollinators.

Santangelo, T. K. A., Johnson M . (2019). "Herbivores and plant defences affect selection on plant reproductive traits more strongly than pollinators." Journal of Evolutionary Biology **32**: 4–18.

Pollinators and herbivores can both affect the evolutionary diversification of plant reproductive traits. However, plant defences frequently alter antagonistic and mutualistic interactions, and therefore, variation in plant defences may alter patterns of herbivore- and pollinator- mediated selection on plant traits. We tested this hypothesis by conducting a common garden field experiment using 50 clonal genotypes of white clover (Trifolium repens) that varied in a Mendelian- inherited chemical antiherbivore defence—the production of hydrogen cyanide (HCN). To evaluate whether plant defences alter herbivore- and/or pollinator- mediated selection, we factorially crossed chemical defence (25 cyanogenic and 25 acyanogenic genotypes), herbivore damage (herbivore suppression) and pollination (hand pollination). We found that herbivores weakened selection for increased inflorescence production, suggesting that large displays are costly in the presence of herbivores. In addition, herbivores weakened selection on flower size but only among acyanogenic plants, suggesting that plant defences reduce the strength of herbivore- mediated selection. Pollinators did not independently affect selection on any trait, although pollinators weakened selection for later flowering among cyanogenic plants. Overall, cyanogenic plant defences consistently increased the strength of positive directional selection on reproductive traits. Herbivores and pollinators both strengthened and weakened the strength of selection on reproductive traits, although herbivores imposed ~2.7× stronger selection than pollinators across all traits. Contrary to the view that pollinators are the most important agents of selection on reproductive traits, our data show that selection on reproductive traits is driven primarily by variation in herbivory and plant defences in this system.

Sargent, R. D., et al. (2007). "Phylogenetic Evidence for a Flower Size and Number Trade-Off." American Journal of Botany **94**(12): 2059-2062.

The size and number of flowers displayed together on an inflorescence (floral display) influences pollinator attraction and pollen transfer and receipt, and is integral to plant reproductive success and fitness. Life history theory predicts that the evolution of floral display is constrained by trade-offs between the size and number of flowers and inflorescences. Indeed, a trade-off between flower size and flower number is a key assumption of models of inflorescence architecture and the evolution of floral display. Surprisingly, however, empirical evidence for the trade-off is limited. In particular, there is a lack of phylogenetic evidence for a trade-off between flower size and number. Analyses of phylogenetic independent contrasts (PICs) of 251 angiosperm species spanning 63 families yielded a significant negative correlation between flower size and flower number. At smaller phylogenetic scales, analyses of individual genera did not always find evidence of a trade-off, a result consistent with previous studies that have examined the trade-off for a single species or genus. Ours is the first study to support an angiosperm-wide trade-off between flower size and number and supports the theory that life history constraints have influenced the evolution of floral display.

Schemske, D. W. and H. D. Bradshaw (1999). "Pollinator preference and the evolution of floral traits in monkeyflowers (Mimulus)." Proceedings of the National Academy of Sciences, USA **96**(21): 11910ⴚ11915.

A paradigm of evolutionary biology is that adaptation and reproductive isolation are caused by a nearly infinite number of mutations of individually small effect. Here, we test this hypothesis by investigating the genetic basis of pollinator discrimination in two closely related species of monkeyflowers that differ in their major pollinators. This system provides a unique opportunity to investigate the genetic architecture of adaptation and speciation because floral traits that confer pollinator specificity also contribute to premating reproductive isolation. We asked: (i) What floral traits cause pollinator discrimination among plant species? and (ii) What is the genetic basis of these traits? We examined these questions by using data obtained from a large-scale field experiment where genetic markers were employed to determine the genetic basis of pollinator visitation. Observations of F2 hybrids produced by crossing bee-pollinated Mimulus lewisii with hummingbird-pollinated Mimulus cardinalis revealed that bees preferred large flowers low in anthocyanin and carotenoid pigments, whereas hummingbirds favored nectar-rich flowers high in anthocyanins. An allele that increases petal carotenoid concentration reduced bee visitation by 80%, whereas an allele that increases nectar production doubled hummingbird visitation. These results suggest that genes of large effect on pollinator preference have contributed to floral evolution and premating reproductive isolation in these monkeyflowers. This work contributes to growing evidence that adaptation and reproductive isolation may often involve major genes.

Schiestl, F. P. (2010). "The evolution of floral scent and insect chemical communication." Ecology Letters **13**(5): 643-656.

Plants have evolved a range of strategies to manipulate the behaviour of their insect partners. One powerful strategy is to produce signals that already have a role in the animals' own communication systems. To investigate to what extent the evolution of floral scents is correlated with chemical communication in insects, I analyse the occurrence, commonness, and evolutionary patterns of the 71 most common 'floral' volatile organic compounds (VOCs) in 96 plant families and 87 insect families. I found an overlap of 87% in VOCs produced by plants and insects. 'Floral' monoterpenes showed strong positive correlation in commonness between plants (both gymnosperms and angiosperms) and herbivores, whereas the commonness of 'floral' aromatics was positively correlated between angiosperms and both pollinators and herbivores. According to a multivariate regression analysis the commonness of 'floral' aromatics was best explained by their commonness in pollinators, whereas monoterpenes were best explained by herbivores. Among pollinator orders, aromatics were significantly more common in Lepidoptera than in Hymenoptera, whereas monoterpenes showed no difference among the two orders. Collectively, these patterns suggest that plants and insects converge in overall patterns of volatile production, both for attraction and defence. Monoterpenes seem to have evolved primarily for defence under selection by herbivores, whereas aromatics evolved signalling functions in angiosperms, primarily for pollinator attraction.

Schiestl, F. P. (2015). "Ecology and evolution of floral volatile-mediated information transfer in plants." New Phytologist **206**(2): 571-577.

Floral volatiles are complex, multi-functional signals that are often used by pollinators in combination with other signals, such as color. Floral visitors use floral scent to estimate the amount of reward present in flowers, to facilitate the identification of a specific host flower or as signals that chemically resemble those important for pollinator insects in other ecological contexts. There is good evidence that floral scent evolves under selection imposed by both mutualists and antagonists. Antagonists may often limit the amount of scent emitted by flowers, thus contributing to spatial population variation, and select for phenotypic plasticity after enemy attack. Floral scent is also an important component of pollinator-mediated reproductive isolation, as it often co-varies with color and morphology in sister species with different pollination systems.

Schiestl, F. P. (2017). "Innate Receiver Bias: Its Role in the Ecology and Evolution of Plant–Animal Interactions." Annual Review of Ecology, Evolution, and Systematics **48**(1): 585-603.

Receiver bias in plant–animal interactions is here defined as “selection mediated by behavioral responses ofanimals, where those responses have evolved in a context outside the interactions.” As a consequence, the responses are not necessarily linked to fitness gains in interacting animals. Thus, receiver bias can help explain seemingly maladaptive patterns of behavior in interacting animals and the evolution of plant traits that trigger such behavior. In this review, I discuss principles of receiver bias, contrast it against mimicry, and outline examples in different plant–animal interactions. The most numerous and best documented examples ofreceiver bias occur within plant–pollinator interactions. I elaborate on the ability ofsome plants to heat up their flowers (i.e., floral thermogenesis) and argue that it likely evolved under receiver bias, especially in pollination systems with oviposition mimicry. Further examples include signals in insect-mediated seed dispersal and plant defense through repellence of aphids. These examples show that receiver bias is widespread in different plant–animal interactions. For a broader understanding of the role of receiver bias in those interactions, we need more data on how animals respond to plant signals, the context and evolutionary history of those behaviors, and the evolutionary patterns of plant signals.

Schiestl, F. P. and S. D. Johnson (2013). "Pollinator-mediated evolution of floral signals." Trends in Ecology & Evolution **28**(5): 307-315.

Because most plants rely on animals for pollination, insights from animal sensory ecology and behavior are essential for understanding the evolution of flowers. In this review, we compare and contrast three main types of pollinator responses to floral signals--receiver bias, 'adaptive' innate preferences, and associative learning--and discuss how they can shape selection on floral signals. We show that pollinator-mediated selection on floral signals can be strong and that the molecular bases of floral signal variation are often surprisingly simple. These new empirical and conceptual insights into pollinator-mediated evolution provide a framework for understanding patterns of both convergent (pollination syndromes) and advergent (floral mimicry) floral signal evolution.

Schiestl, F. P., et al. (2014). "Herbivory and floral signaling: phenotypic plasticity and tradeoffs between reproduction and indirect defense." New Phytologist **203**(1): 257-266.

Plant defense against herbivores may compromise attraction of mutualists, yet information remains limited about the mechanisms underlying such signaling tradeoffs. Here, we investigated the effects of foliar herbivory by two herbivore species on defense compounds, floral signaling, pollinator and parasitoid attraction, and seed production. Herbivory generally reduced the quantity of many floral volatile organic compounds VOCs) in Brassica rapa. By contrast, floral color, flower diameter, and plant height remained unaffected. The decreased amounts of floral volatiles led to reduced attractiveness of flowers to pollinators, but increased the attractiveness of herbivore-infested plants to parasitoids. Plants infested with the native butterfly Pieris brassicae produced more flowers during early flowering, effectively compensating for the lower olfactory attractiveness. Herbivory by the invasive Spodoptera littoralis increased the amounts of glucobrassicanapin, and led to delayed flowering. These plants tended to attract fewer pollinators and to produce fewer seeds. Our study indicates a tradeoff between pollinator attraction and indirect defense (parasitoid attraction), which can be mitigated by reduced floral VOC emission and production of more early flowers. We suggest that this compensatory mechanism is specific to plant-herbivore associations with a coevolutionary history.

Sercu, B. K., et al. (2020). "Induced phenological avoidance: A neglected defense mechanism against seed predation in plants." Journal of Ecology **108**(3).

1. Flowering phenology is an important life-history trait affecting plant reproductive performance and is influenced by various abiotic and biotic factors. Pre-dispersal seed predation and pollination are expected to impose counteracting selection pressure on flowering phenology, with pre-dispersal seed predation expected to favour off-peak flowering and pollination to favour synchronous flowering. 2. Here we studied the effect of pre-dispersal seed predation by the beetle Byturus ochraceus, a specialist seed herbivore, on the flowering phenology of Geum urbanum. This forest understorey plant species is self-pollinating, so that the influence of seed predation can be studied independent from pollination. We measured in detail the timing and predation rate of individual flowers during two consecutive years in more than 60 individuals. We tested the hypotheses that pre-dispersal seed predation exerts selection for within-season compensatory flowering as well as for induced phenological avoidance in the following season. 3. We found no indication for compensatory flowering within a growing season, but plants that experienced predation shifted their flowers to the end of the flowering season the subsequent year. This induced phenological avoidance points to a plastic response to pre-dispersal seed predation that may be adaptive. Importantly, the delay in flower production came at a cost, since flowers later in the season had a reduced seed output, presumably because of increasing light limitation following forest canopy closure. 4. Synthesis. Herbivory by specialist enemies can cause serious fitness decline in hosts. We here show that induced shifts in phenology can form an important defense strategy against pre-dispersal seed predation. The induced mismatches between herbivore and host phenology are anticipated to be adaptive when herbivory is predictable across successive flowering periods.

Silveira, T. A., et al. (2018). "Phloem-feeding herbivory on flowering melon plants enhances attraction of parasitoids by shifting floral to defensive volatiles." Arthropod-Plant Interactions **12**(5): 751-760.

Emission of herbivore-induced plant volatiles (HIPVs) can differ according to the type of herbivory and the plant development stage, ultimately affecting recruitment of the natural enemy. Little is known about plant defenses induced at the flowering stage by phloem-feeding insects. We investigated the olfactory preference of Encarsia desantisi parasitoids and the chemical profile of flowering melon plants induced or not by the phloem-feeding of Bemisia tabaci whiteflies. In addition, we tested whether the parasitoids were attracted to synthetic defensive HIPVs, which mimicked whitefly-infested flowering melons. The parasitoids recognized volatiles from undamaged melons but preferred the scent of host-infested melons in olfactometry assays. Amounts of most individual volatiles did not differ between plant treatments; however, only whitefly-induced melons released methyl salicylate and tetradecane, compounds known to attract parasitoids. Interestingly, grouping volatiles by chemical classes revealed that whitefly-infested melon released larger amounts of monoterpenes and smaller amounts of benzenoids than undamaged melons, which might underlying the parasitoid attraction and indicate a possible trade-off between defensive and reproductive defenses at the melon flowering stage. Additionally, E. desantisi preferred the mix of synthetic and defensive HIPVs over hexane (control), opening a new avenue for further investigations in using olfactory lures for B. tabaci biological control. This study is the first report of induced defenses in melon plants and their mediation in a tritrophic interaction, as well as the first record of E. desantisi behavioral preference for HIPVs.

Smallegange, R. C., et al. (2007). "Flower vs. leaf feeding by Pieris brassicae: glucosinolate-rich flower tissues are preferred and sustain higher growth rate." Journal of Chemical Ecology **33**(10): 1831-1844.

Interactions between butterflies and caterpillars in the genus Pieris and plants in the family Brassicaceae are among the best explored in the field of insect-plant biology. However, we report here for the first time that Pieris brassicae, commonly assumed to be a typical folivore, actually prefers to feed on flowers of three Brassica nigra genotypes rather than on their leaves. First- and second-instar caterpillars were observed to feed primarily on leaves, whereas late second and early third instars migrated via the small leaves of the flower branches to the flower buds and flowers. Once flower feeding began, no further leaf feeding was observed. We investigated growth rates of caterpillars having access exclusively to either leaves of flowering plants or flowers. In addition, we analyzed glucosinolate concentrations in leaves and flowers. Late-second- and early-third-instar P. brassicae caterpillars moved upward into the inflorescences of B. nigra and fed on buds and flowers until the end of the final (fifth) instar, after which they entered into the wandering stage, leaving the plant in search of a pupation site. Flower feeding sustained a significantly higher growth rate than leaf feeding. Flowers contained levels of glucosinolates up to five times higher than those of leaves. Five glucosinolates were identified: the aliphatic sinigrin, the aromatic phenylethylglucosinolate, and three indole glucosinolates: glucobrassicin, 4-methoxyglucobrassicin, and 4-hydroxyglucobrassicin. Tissue type and genotype were the most important factors affecting levels of identified glucosinolates. Sinigrin was by far the most abundant compound in all three genotypes. Sinigrin, 4-hydroxyglucobrassicin, and phenylethylglucosinolate were present at significantly higher levels in flowers than in leaves. In response to caterpillar feeding, sinigrin levels in both leaves and flowers were significantly higher than in undamaged plants, whereas 4-hydroxyglucobrassicin leaf levels were lower. Our results show that feeding on flower tissues, containing higher concentrations of glucosinolates, provides P. brassicae with a nutritional benefit in terms of higher growth rate. This preference appears to be in contrast to published negative effects of volatile glucosinolate breakdown products on the closely related Pieris rapae.

Sobral, M., et al. (2015). "Selective Pressures Explain Differences in Flower Color among Gentiana lutea Populations." PLoS One **10**(7): e0132522.

Flower color variation among plant populations might reflect adaptation to local conditions such as the interacting animal community. In the northwest Iberian Peninsula, flower color of Gentiana lutea varies longitudinally among populations, ranging from orange to yellow. We explored whether flower color is locally adapted and the role of pollinators and seed predators as agents of selection by analyzing the influence of flower color on (i) pollinator visitation rate and (ii) escape from seed predation and (iii) by testing whether differences in pollinator communities correlate with flower color variation across populations. Finally, (iv) we investigated whether variation in selective pressures explains flower color variation among 12 G. lutea populations. Flower color influenced pollinator visits and differences in flower color among populations were related to variation in pollinator communities. Selective pressures on flower color vary among populations and explain part of flower color differences among populations of G. lutea. We conclude that flower color in G. lutea is locally adapted and that pollinators play a role in this adaptation.

Soltis, P. S. and D. E. Soltis (2014). "Flower diversity and angiosperm diversification." Methods in Molecular Biology **1110**: 85-102.

The flower itself, which comprises most of the evolutionary innovations of flowering plants, bears special significance for understanding the origin and diversification of angiosperms. The sudden origin of angiosperms in the fossil record poses unanswered questions on both the origins of flowering plants and their rapid spread and diversification. Central to these questions is the role that the flower, and floral diversity, played. Recent clarifications of angiosperm phylogeny provide the foundation for investigating evolutionary transitions in floral features and the underlying genetic mechanisms of stasis and change. The general features of floral diversity can best be addressed by considering key patterns of variation: an undifferentiated versus a differentiated perianth; elaboration of perianth organs in size and color; merosity of the flower; and phyllotaxy of floral organs. Various models of gene expression now explain the regulation of floral organization and floral organ identity; the best understood are the ABC(E) model and its modifications, but other gene systems are important in specific clades and require further study. Furthermore, the propensity for gene and genome duplications in angiosperms provides abundant raw material for novel floral features--emphasizing the importance of understanding the conservation and diversification of gene lineages and functions in studies of macroevolution.

Strauss, S. Y. and A. A. Agrawal (1999). "The ecology and evolution of plant tolerance to herbivory." Trends in Ecology & Evolution **14**(5): 179-185.

The tolerance of plants to herbivory reflects the degree to which a plant can regrow and reproduce after damage from herbivores. Autoecological factors, as well as the influence of competitors and mutualists, affect the level of plant tolerance. Recent work indicates that there is a heritable basis for tolerance and that it can evolve in natural plant populations. Although tolerance is probably not a strict alternative to plant resistance, there could be inter- and intraspecific tradeoffs between these defensive strategies.

Strauss, S. Y. and J. B. Whittall (2006). Non-pollinator agents of selection on floral traits, Oxford University Press.

Despite the dominating role of pollinators in floral evolution, mounting evidence reveals significant additional, often antagonistic, influences of abiotic and biotic non-pollinator agents. Even when pollinators and other agents impose selection on floral traits in the same direction, the role of other agents is frequently overlooked. Maintenance of genetic variation in floral traits and divergence from trait optima for pollination can result from both indirect selection on correlated traits and direct selection on floral traits. For example, in numerous species, periods of heat or drought favour pink- or purple-flowered individuals over white-flowered ones, because associated anthocyanins in vegetative tissues enhance stress tolerance. Conflicting selection on floral traits may also occur directly when floral antagonists and mutualists share the same preferences. We review the evidence for influences of abiotic and biotic non–pollinator agents of selection on several floral traits: petal colour, flower and display size, flower shape, nectar composition, flowering phenology, and breeding system. Despite growing evidence of the importance of non-pollinator selection, few studies have explored the relative strength of selection from pollinators versus other sources. In several cases, pollinators are not the strongest current source of selection on floral traits, despite perhaps being the driving factor shaping floral traits historically. Future studies will benefit from a synthetic approach that recognizes the entire ecological context of floral adaptation and combines field experiments with genetic studies to determine the relative roles of pollinators and non-pollinator agents in floral evolution. The study of floral evolution will be enhanced by approaches that incorporate a broader context that includes both abiotic and biotic agents of selection.

Takeno, K. (2016). "Stress-induced flowering: the third category of flowering response." Journal of Experimental Botany **67**(17): 4925-4934.

The switch from vegetative growth to reproductive growth, i.e. flowering, is the critical event in a plant's life. Flowering is regulated either autonomously or by environmental factors; photoperiodic flowering, which is regulated by the duration of the day and night periods, and vernalization, which is regulated by low temperature, have been well studied. Additionally, it has become clear that stress also regulates flowering. Diverse stress factors can induce or accelerate flowering, or inhibit or delay it, in a wide range of plant species. This article focuses on the positive regulation of flowering via stress, i.e. the induction or acceleration of flowering in response to stress that is known as stress-induced flowering - a new category of flowering response. This review aims to clarify the concept of stress-induced flowering and to summarize the full range of characteristics of stress-induced flowering from a predominately physiological perspective.

Teixido, A. L., et al. (2016). "Size Matters: Understanding the Conflict Faced by Large Flowers in Mediterranean Environments." The Botanical Review **82**(2): 204-228.

Flower size is a key trait in the reproductive ecology of animal-pollinated plants. However, pollinator-mediated selection does not always modulate this trait and environmental conditions and/or antagonist interactions may favor smaller flowers. We evaluate the occurrence of a large-flowered family in a hot and dry Mediterranean environment, mediated by a cost-benefit balance and a male–female conflict. Large flowers have sizeable benefits in terms of pollination and reproductive success and pollinators mediate selection through male function, but female fitness is contextdependent. High floral production and maintenance costs and florivore incidence in large flowers limit female function, which counteracts pollinator-mediated selection. Large flowers are highly costly in the Mediterranean and flower size is mediated by a sexual conflict between the benefits of male function and the costs of the female one. However, a short floral longevity, occasional pollen limitation and selection through maleness keep the existence of large flowers in these environments.

Teixido, A. L., et al. (2011). "Flower size and longevity influence florivory in the large-flowered shrub Cistus ladanifer." Acta Oecologica **37**(5): 418-421.

Plants with larger and longer-lived flowers receive more pollinator visits and increase reproductive success, though may also suffer more from antagonistic interactions with animals. Florivores can reduce fruit and seed production, so selection on flower size, floral longevity and/or number offlowers may thus be determined by the relative effects of both pollinators and florivores. In this study flowers of Cistus ladanifer, a large-flowered Mediterranean shrub, were monitored to evaluate the effects of flower size, floral longevity and number of flowers on levels of florivory in four populations. Number of flowers was variable but did not differ among populations. Both flower size and floral longevity of C. ladanifer showed broad variation and significantly differed among populations. Overall, 7% of flowers suffered attack by florivores, which were mainly ants picking the stamens and beetles consuming petals and pollen. Within-populations, larger and longer-lived flowers tended to be affected by florivores more frequently. The low overall incidence offlorivores and its lack of between-population variation suggest that florivory may not influence intraspecific variation of these floral traits. However, moderate florivory levels on the largest and longest-lived flowers open the possibility of exerting selection towards smaller and shorter-lived flowers in some of the populations studied.

Thackeray, S. J., et al. (2016). "Phenological sensitivity to climate across taxa and trophic levels." Nature **535**(7611): 241-245.

Differences in phenological responses to climate change among species can desynchronise ecological interactions and thereby threaten ecosystem function. To assess these threats, we must quantify the relative impact of climate change on species at different trophic levels. Here, we apply a Climate Sensitivity Profile approach to 10,003 terrestrial and aquatic phenological data sets, spatially matched to temperature and precipitation data, to quantify variation in climate sensitivity. The direction, magnitude and timing of climate sensitivity varied markedly among organisms within taxonomic and trophic groups. Despite this variability, we detected systematic variation in the direction and magnitude of phenological climate sensitivity. Secondary consumers showed consistently lower climate sensitivity than other groups. We used mid-century climate change projections to estimate that the timing of phenological events could change more for primary consumers than for species in other trophic levels (6.2 versus 2.5-2.9 days earlier on average), with substantial taxonomic variation (1.1-14.8 days earlier on average).

Theis, N. and L. S. Adler (2012). "Advertising to the enemy: enhanced floral fragrance increases beetle attraction and reduces plant reproduction." Ecology **93**(2): 430–435.

Many organisms face challenges in avoiding predation while searching for mates. For plants, emitting floral fragrances to advertise reproductive structures could increase the attraction of detrimental insects along with pollinators. Very few studies have experimentally evaluated the costs and benefits of fragrance emission with explicit consideration of how plant fitness is affected by both pollinators and florivores. To determine the reproductive consequences of increasing the apparency of reproductive parts, we manipulated fragrance, pollination, and florivores in the wild Texas gourd, Cucurbita pepo var. texana. With enhanced fragrance we found an increase in the attraction of florivores, rather than pollinators, and a decrease in seed production. This study is the first to demonstrate that enhanced floral fragrance can increase the attraction of detrimental florivores and decrease plant reproduction, suggesting that florivory as well as pollination has shaped the evolution of floral scent.

Thompson, J. D. (2001). "How do visitation patterns vary among pollinators in relation to floral display and floral design in a generalist pollination system?" Oecologia **126**(3): 386-394.

Diverse pollinator assemblages may impose complex selection and thus limit specialisation to particular pollinators. Previous work has concentrated on how visitation rates of different pollinators vary in space and time and how pollinators may vary in efficiency. In this study I quantify variation in visitation rates and foraging behaviour of different insect types (1) in space and time and (2) in relation to variation in floral design (flower size and form) and floral display (number of open flowers) for the distylous clonal shrub Jasminum fruticans. Mean visitation rate showed a significant interaction between insect type and population for seven populations in one year, and between insect types and years for a single population over 3 years. There was also a significant interaction between insect type and population for the proportion of flowers visited. In general the number of visits was positively related to the number of open flowers in a patch, but analyses by insect type showed that this was only true for bee flies and butterflies. Short-tongued bees showed a positive relationship between visitation rate and the number of open flowers on the focal stem, and hawkmoths and butterflies made more visits to plants with larger flowers. Hawkmoths were the only insect type to show a positive relation between the number of flowers visited per foraging bout and flower size. The significant differences between different insect types in patterns of variation in visitation rates in response to floral design and display may act to diversify selection on floral traits, and thereby constrain specialisation of the plant to particular pollinators.

Trager, M. D., et al. (2010). "Benefits for plants in ant-plant protective mutualisms: a meta-analysis." PLoS One **5**(12): e14308.

Costs and benefits for partners in mutualistic interactions can vary greatly, but surprisingly little is known about the factors that drive this variation across systems. We conducted a meta-analysis of ant-plant protective mutualisms to quantify the effects of ant defenders on plant reproductive output, to evaluate if reproductive effects were predicted from reductions in herbivory and to identify characteristics of the plants, ants and environment that explained variation in ant protection. We also compared our approach with two other recent meta-analyses on ant-plant mutualisms, emphasizing differences in our methodology (using a weighted linear mixed effects model) and our focus on plant reproduction rather than herbivore damage. Based on 59 ant and plant species pairs, ant presence increased plant reproductive output by 49% and reduced herbivory by 62%. The effects on herbivory and reproduction within systems were positively correlated, but the slope of this relationship (0.75) indicated that tolerance to foliar herbivory may be a common plant response to absence of ant guards. Furthermore, the relationship between foliar damage and reproduction varied substantially among systems, suggesting that herbivore damage is not a reliable surrogate for fitness consequences of ant protection. Studies that experimentally excluded ants reported a smaller effect of ant protection on plant reproduction than studies that relied upon natural variation in ant presence, suggesting that study methods can affect results in these systems. Of the ecological variables included in our analysis, only plant life history (i.e., annual or perennial) explained variation in the protective benefit of mutualistic ants: presence of ants benefitted reproduction of perennials significantly more than that of annuals. These results contrast with other quantitative reviews of these relationships that did not include plant life history as an explanatory factor and raise several questions to guide future research on ant-plant protection mutualisms.

Troth, A., et al. (2018). "Selective trade-offs maintain alleles underpinning complex trait variation in plants." Science **361**(6401): 475-478.

To understand evolutionary factors that maintain complex trait variation, we sequenced genomes from a single population of the plant Mimulus guttatus, identifying hundreds of nucleotide variants associated with morphological and life history traits. Alleles that delayed flowering also increased size at reproduction, which suggests pervasive antagonistic pleiotropy in this annual plant. The “large and slow” alleles, which were less common in small, rapidly flowering populations, became more abundant in populations with greater plant size. Furthermore, natural selection within the field population favored alternative alleles from year to year. Our results suggest that environmental fluctuations and selective trade-offs maintain polygenic trait variation within populations and also contribute to the geographic divergence in this wildflower species.

van der Kooi, C. J., et al. (2021). "Mutualisms and (A)symmetry in Plant-Pollinator Interactions." Curr Biol **31**(2): R91-R99.

The majority of flowering plants relies on animal pollinators for sexual reproduction and many animal pollinators rely on floral resources. However, interests of plants and pollinators are often not the same, resulting in an asymmetric relationship that ranges from mutualistic to parasitic interactions. Our understanding of the processes that underlie this asymmetry remains fragmentary. In this Review, we bring together evidence from evolutionary biology, plant chemistry, biomechanics, sensory ecology and behaviour to illustrate that the degree of symmetry often depends on the perspective taken. We also highlight variation in (a)symmetry within and between plant and pollinator species as well as between geographic locations. Through taking different perspectives from the plant and pollinator sides we provide new ground for studies on the maintenance and evolution of animal pollination and on the (a)symmetry in plant-pollinator interactions.

Van Kleunen, M., et al. (2007). "The role of beetle marks and flower colour on visitation by monkey beetles (hopliini) in the greater cape floral region, South Africa." Ann Bot **100**(7): 1483-1489.

BACKGROUND AND AIMS: A deviation from the classical beetle pollination syndrome of dull-coloured flowers with an unpleasant scent is found in the Greater Cape Floral Region of South Africa. Here, monkey beetles (Scarabaeidae) visit brightly coloured, odourless flowers with conspicuous dark spots and centres (beetle marks). The role of flower colour and markings in attracting monkey beetles is still poorly understood. METHODS: Artificial model flowers with different marking patterns were used to test the effect of beetle marks on visitation by monkey beetles. To test whether monkey beetles are conditioned to the colour of the local matrix species, model flowers of different colours were placed in populations of three differently coloured species of Iridaceae. KEY RESULTS: Among all three matrix species the presence of dark markings of some kind (either centres or spots) increased visitation rates but the different matrix species differed in whether the effect was due to a dark centre or to dark spots. Monkey beetles were not conditioned for the colour of the matrix species: model colour was not significant in the Hesperantha vaginata and in the Romulea monadelpha matrices, whereas yellow model flowers were preferred over orange ones in the orange-flowered Sparaxis elegans matrix. CONCLUSIONS: This study is the first to demonstrate that beetle marks attract pollinating monkey beetles in the Greater Cape Floral Region. In contrast to plants with the classical beetle pollination syndrome that use floral scent as the most important attractant of pollinating beetles, plants with the monkey beetle pollination syndrome rely on visual signals, and, in some areas at least, monkey beetles favour flowers with dark beetle markings over unmarked flowers.

Veiga, T., et al. (2015). "Are pollinators and seed predators selective agents on flower color in Gentiana lutea?" Evolutionary Ecology **29**(3): 451-464.

Animals which interact with plants often cause selective pressures on plant traits. Flower color variation within a species might be shaped by the action of animals feeding on the plant species. Pollinators might exert natural selection on color if flower color is related to their foraging efficiency. For example, some pollinator species might require more time to detect particular colors. If that is the case, flower color might have evolved as a pollination exploitation barrier—ensuring that flowers are more visited by the most efficient pollinators. In addition, non-pollinator agents such as predispersal seed predators may select on flower color, if color indicates food resources (seeds) or if color is related to deterrent compounds. We address selection on flower color in a population of Gentiana lutea where color varies among individuals from yellow to orange. We hypothesize that opposed selection from mutualists (pollinators) and antagonists (predispersal seed predators) maintains flower color variation in this population. By means of path analysis we addressed the role of both interactors in flower color selection. We found that selection acts on flower color, mediated by both pollinators and seed predators. Both agents favored yellow-flowered individuals, thus selection by pollinators and seed predators does not maintain flower color variation in this population.

Venail, J., et al. (2010). "Speciation genes in the genus Petunia." Philosophical Transactions of the Royal Society B: Biological Sciences **365**(1539): 461-468.

A major innovation in angiosperms is the recruitment of animal pollinators as a means to enhance the efficiency and specificity of pollen transfer. The implementation of this reproductive strategy involved the rapid and presumably coordinated evolution of multiple floral traits. A major question concerns the molecular identity of the genetic polymorphisms that specify the phenotypic differences between distinct pollination syndromes. Here, we report on our work with Petunia, an attractive model system for quantitative plant genetics and genomics. From interspecific crosses, we obtained F2 plants that differed in the length of the floral tube or the size of the limb. We used these plants to study the behaviour of the hawkmoth pollinator, Manduca sexta. Plants with larger limbs were preferentially visited, consistent with the notion that flower size affects visibility under low light conditions. The moths also displayed an innate preference for shorter tubes. However, in those cases that flowers with long tubes were chosen, the animals fed for equal time. Thus, the perception of tube length may help the moths, early on, to avoid those plants that are more difficult to handle.

Vereecken, N. J., et al. (2010). "Hybrid floral scent novelty drives pollinator shift in sexually deceptive orchids." BMC Evolutionary Biology **10**: 103.

BACKGROUND: Sexually deceptive orchids of the genus Ophrys attract their pollinators, male insects, on a highly specific basis through the emission of odour blends that mimic the female sex pheromone of the targeted species. In this study, we have investigated a contact site between Ophrys arachnitiformis and O. lupercalis, two sympatric orchid species that are usually reproductively isolated via the exploitation of different pollinator "niches", but occasionally hybridise despite their apparent combination of ethological and mechanical isolation barriers. In particular, we have investigated the extent to which these Ophrys hybrids generate "emergent" combinations (i.e. novel and unpredictable from the parents' phenotypes) of floral traits, and how these phenotypic novelties, particularly the odour blends emitted by the flower, could facilitate the invasion of a novel pollinator "niche" and induce the rapid formation of reproductive isolation, a prerequisite for adaptive evolutionary divergence. RESULTS: Our chemical analyses of floral scents show that the Ophrys F1 hybrids investigated here produce more compounds, significantly different ratios (% of odour compounds in the total blend), as well as new compounds in their floral odour compared to their progenitors. When tested for their attractiveness to the pollinator of each parent orchid species, we found that floral scent extracts of the hybrids triggered less inspecting flights and contacts by the male bees with the scented dummy than those of the parental orchid species. However, a series of additional behavioural bioassays revealed that the novel floral scent of the hybrids was significantly more attractive than either of the two parents to a pollinator species not initially involved in the pollination of any of the parent Ophrys species. CONCLUSIONS: Collectively, our results illustrate that the process of hybridisation can lead to the generation of evolutionary novelties, and that novel combinations of floral traits can drive pollinator shifts and rapid reproductive isolation in highly specific plant-pollinator interactions.

Villamil, N., et al. (2019). "Testing the Distraction Hypothesis: Do extrafloral nectaries reduce ant-pollinator conflict?" J Ecol **107**(3): 1377-1391.

Ant guards protect plants from herbivores, but can also hinder pollination by damaging reproductive structures and/or repelling pollinators. Natural selection should favour the evolution of plant traits that deter ants from visiting flowers during anthesis, without waiving their defensive services. The Distraction Hypothesis posits that rewarding ants with extrafloral nectar could reduce their visitation of flowers, reducing ant-pollinator conflict while retaining protection of other structures.We characterised the proportion of flowers occupied by ants and the number of ants per flower in a Mexican ant-plant, Turnera velutina. We clogged extrafloral nectaries on field plants and observed the effects on patrolling ants, pollinators and ants inside flowers, and quantified the effects on plant fitness. Based on the Distraction Hypothesis, we predicted that preventing extrafloral nectar secretion should result in fewer ants active at extrafloral nectaries, more ants inside flowers and a higher proportion of flowers occupied by ants, leading to ant-pollinator conflict, with reduced pollinator visitation and reduced plant fitness.Overall ant activity inside flowers was low. Preventing extrafloral nectar secretion through clogging reduced the number of ants patrolling extrafloral nectaries, significantly increased the proportion of flowers occupied by ants from 6.1% to 9.7%, and reduced plant reproductive output through a 12% increase in the probability of fruit abortion. No change in the numbers of ants or pollinators inside flowers was observed. This is the first support for the Distraction Hypothesis obtained under field conditions, showing ecological and plant fitness benefits of the distracting function of extrafloral nectar during anthesis. Synthesis. Our study provides the first field experimental support for the Distraction Hypothesis, suggesting that extrafloral nectaries located close to flowers may bribe ants away from reproductive structures during the crucial pollination period, reducing the probability of ant occupation of flowers, reducing ant-pollinator conflict and increasing plant reproductive success.

Von Arx, M., et al. (2012). "Floral humidity as a reliable sensory cue for profitability assessment by nectar-foraging hawkmoths." Proceedings of the National Academy of Sciences, USA **109**(24): 9471-9476.

Most research on plant–pollinator communication has focused on

sensory and behavioral responses to relatively static cues. Floral rewards such as nectar, however, are dynamic, and foraging animals will increase their energetic profit if they can make use of floral cues that more accurately indicate nectar availability. Here we document such a cue—transient humidity gradients—using the night blooming flowers of Oenothera cespitosa (Onagraceae). The headspace of newly opened flowers reaches levels of about 4% above ambient relative humidity due to additive evapotranspirational water loss through petals and water-saturated air from the nectar tube. Floral humidity plumes differ from ambient levels only during the first 30 min after anthesis (before nectar is depleted in wild populations), whereas other floral traits (scent, shape, and color) persist for 12–24 h. Manipulative experiments indicated that floral humidity gradients are mechanistically linked to nectar volume and therefore contain information about energy rewards to floral visitors. Behavioral assays with Hyles lineata (Sphingidae) and artificial flowers with appropriate humidity gradients suggest that these hawkmoth pollinators distinguish between subtle differences in relative humidity when other floral cues are held constant. Moths consistently approached and probed flowers with elevated humidity over those with ambient humidity levels. Because floral humidity gradients are largely produced by the evaporation of nectar itself, they represent condition-informative cues that facilitate remote sensing of floral profitability by discriminating foragers. In a xeric environment, this level of honest communication should be adaptive when plant reproductive success is pollinator limited, due to intense competition for the attention of a specialized pollinator.

Yang, L. H. and V. H. Rudolf (2010). "Phenology, ontogeny and the effects of climate change on the timing of species interactions." Ecology Letters **13**(1): 1-10.

Climate change is altering the phenology of many species and the timing of their interactions with other species, but the impacts of these phenological shifts on species interactions remain unclear. Classical approaches to the study of phenology have typically documented changes in the timing of single life-history events, while phenological shifts affect many interactions over entire life histories. In this study, we suggest an approach that integrates the phenology and ontogeny of species interactions with a fitness landscape to provide a common mechanistic framework for investigating phenological shifts. We suggest that this ontogeny-phenology landscape provides a flexible method to document changes in the relative phenologies of interacting species, examine the causes of these phenological shifts, and estimate their consequences for interacting species.
